# Lung cancer deficient in the tumor suppressor GATA4 is sensitive to TGFBR1 inhibition

**DOI:** 10.1038/s41467-019-09295-7

**Published:** 2019-04-10

**Authors:** Lei Gao, Yong Hu, Yahui Tian, Zhenzhen Fan, Kun Wang, Hongdan Li, Qian Zhou, Guandi Zeng, Xin Hu, Lei Yu, Shiyu Zhou, Xinyuan Tong, Hsinyi Huang, Haiquan Chen, Qingsong Liu, Wanting Liu, Gong Zhang, Musheng Zeng, Guangbiao Zhou, Qingyu He, Hongbin Ji, Liang Chen

**Affiliations:** 1 Key Laboratory of Functional Protein Research of Guangdong Higher Education, Institute of Life and Health Engineering, College of Life Science and Technology, Jinan University, 510632 Guangzhou, China; 2 College of Life Sciences, Beijing Normal University, 100875 Beijing, China; 3 College of Biological Sciences, China Agricultural University, 100094 Beijing, China; 4 Key Laboratory of Molecular Imaging, Institute of Automation, Chinese Academy of Sciences, 100190 Beijing, China; 5 The University of Texas Health Science Center at Houston (UTHealth), 2450 Holcombe Blvd., Suite 1, Houston, TX 77021 USA; 6 Beijing Tongren Hospital, Capital Medical University, 100730 Beijing, China; 7 State Key Laboratory of Cell Biology, Shanghai Institutes for Biological Sciences, Chinese Academy of Sciences, 200031 Shanghai, China; 8 CAS Center for Excellence in Molecular Cell Science, Shanghai Institutes for Biological Sciences, Chinese Academy of Sciences, 200031 Shanghai, China; 9 Innovation Center for Cell Signaling Network, Institute of Biochemistry and Cell Biology, Shanghai Institutes for Biological Sciences, Chinese Academy of Sciences, 200031 Shanghai, China; 10 University of Chinese Academy of Sciences, Beijing, China; 11 Department of Thoracic Surgery, Fudan University Shanghai Cancer Center, 200032 Shanghai, China; 12 High Magnetic Field Laboratory, Chinese Academy of Sciences, 230031 Hefei, Anhui China; 13 Department of Experimental Research, Sun Yat-sen University Cancer Center, Guangzhou, China; 14 State Key Laboratory of Molecular Oncology, National Cancer Center/Cancer Hospital, Chinese Academy of Medical Sciences and Peking Union Medical College, 100021 Beijing, China; 15 School of Life Science and Technology, Shanghai Tech University, 200120 Shanghai, China

**Keywords:** Targeted therapies, Non-small-cell lung cancer, Cancer models

## Abstract

Lung cancer is the leading cause of cancer-related deaths worldwide. Tumor suppressor genes remain to be systemically identified for lung cancer. Through the genome-wide screening of tumor-suppressive transcription factors, we demonstrate here that GATA4 functions as an essential tumor suppressor in lung cancer in vitro and in vivo. Ectopic GATA4 expression results in lung cancer cell senescence. Mechanistically, GATA4 upregulates multiple miRNAs targeting *TGFB2* mRNA and causes ensuing WNT7B downregulation and eventually triggers cell senescence. Decreased GATA4 level in clinical specimens negatively correlates with WNT7B or TGF-β2 level and is significantly associated with poor prognosis. TGFBR1 inhibitors show synergy with existing therapeutics in treating GATA4-deficient lung cancers in genetically engineered mouse model as well as patient-derived xenograft (PDX) mouse models. Collectively, our work demonstrates that GATA4 functions as a tumor suppressor in lung cancer and targeting the TGF-β signaling provides a potential way for the treatment of GATA4-deficient lung cancer.

## Abstract (web summary)

The tumor suppressor GATA4 is frequently epigenetically silenced in lung cancer. In this study, Gao et al. demonstrate that GATA4 regulates the expression of TGFBR2 and that TGFRB1 inhibitors can synergise with chemotherapeutics to inhibit the growth of GATA4-deficient tumors in mice.

## Introduction

Non-small cell lung cancer (NSCLC), the leading cause of cancer-related deaths, is responsible for estimated 1.6 million deaths as of the year 2012^1,2^. Lung adenocarcinoma is the most common type of NSCLC^3^, highlighting the urgent need for novel therapeutic approaches.

Tumor suppressor genes (TSGs) inhibit tumor formation and metastasis mainly through the induction of cell-cycle arrest, apoptosis, and/or senescence^4^. They achieve these biological impacts via regulating diverse cellular activities, including DNA damage responses, tumor angiogenesis, protein ubiquitination and degradation, mitogenic signaling, cell specification, differentiation, and migration^5^. Moreover, inactivation of TSG modulates tumor cells’ response to current therapies^6,7^.

Transcription factors (TFs), especially master TFs, play dominant roles in maintaining the phenotype of a particular tissue type by interacting with the super enhancers^8^. Not surprisingly, TFs frequently function as TSGs^9–12^. Despite of the importance of TFs in tumorigenesis and their impact on the response of tumor cells to treatment, a systemic assay of TSG TFs remains to be determined in lung cancer.

GATA4 belongs to the zinc finger transcription factor family which consists of six members from GATA 1 to GATA 6. The structure of GATA4 features family-specific two N-terminal transcription activation domains (TAD), two central zinc finger domains (ZF), a nuclear localizing signal (NLS) immediately C-terminal to ZF2, and a C-terminal region (CTR)^13^. GATA4 binds to the consensus sequence, A/TGATAA/G^14^, in a highly dynamic manner to regulate numerous target gene expression during the process of organogenesis^15^ and in response to environmental cues^16,17^. GATA4 is therefore considered as a pioneer modifier that opens up a closed chromatin to facilitate binding of TFs including itself to the target sites^18^. Moreover, GATA4 activity is subjected to the regulation by various types of post-translational modifications, including phosphorylation^13,19^, acetylation^20,21^, methylation^22^, and SUMOylation^23^. Not surprisingly, GATA4 is recognized as the critical controller for cell fate.

GATA4 plays a pivotal role during lung development. Missense mutation of *GATA4* (V238G) causes abnormal lung structure and embryonic lethality in mice^24^. Clinical studies reported frequent hypermethylation of the *GATA4* promoter in human lung cancer samples but not in paired normal lungs^25–27^. Despite of the fact that GATA4 is widely epigenetically silenced in lung cancer, the impacts of GATA4 silencing on tumorigenesis and corresponding cancer therapeutic strategies remain largely unexplored.

Here, we have performed a genome-wide screening of TFs to identify potential TSGs in lung cancer. We find that GATA4 is an essential TSG and further demonstrate that the hyperactivated TGF-β-TGFBRs-SMAD-Wnt signaling axis serves as potential target for treating GATA4-deficient lung cancer.

## Results

### GATA4 is an essential tumor suppressor in lung cancer

**Fig. 1:**
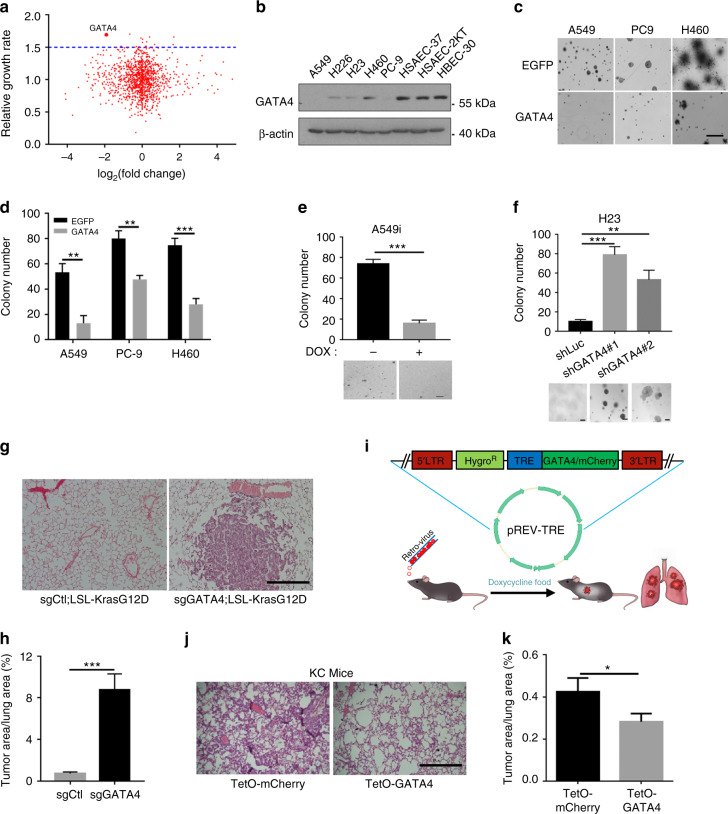
GATA4 is an important tumor suppressor in lung cancer. **a** Scatterplot of relative growth rate against relative expression level. 10,000 H23 cells per well seeded in 96-well plate; siRNA transfected into individual wells. CCK8 value of a well 3 days post siRNA transfection was plotted against relative expression value of the corresponding gene in tumor (one replica). **b** Western blot analysis of GATA4 expression in cancerous and normal lung cell lines. **c** Lung cancer cells infected with lentivirus for ectopic expression of GATA4. Soft-agar colony forming ability was compared between lung cancer cell lines (A549, PC-9, and H460) ectopically expressing GATA4 and EGFP. Scale = 500 μm. **d** Statistics of soft-agar colony result shown in **c** (*n* = 3 per group). **e** Colony number of 10,000 A549i cells in the absence or presence of Dox checked 14 days after seeding in soft agar plate. Upper panel: statistics of the soft-agar colonies. Lower panel: representative pictures of A549i cells treated with or without 2 μg/mL of Dox (*n* = 3 per group, scale = 100 μm) (A549i is an engineered A549 clone harboring Dox inducible GATA4 expression gene element). **f** Soft-agar colony forming ability of stable H23 with GATA4 knockdown by shRNAs or control shRNA. Upper panel: statistics of soft-agar colony result of H23 knockdown with control or GATA4 targeting shRNAs. Lower panel: Representative pictures (*n* = 3 per group, scale = 50 μm). **g** Lsl-KRAS^G12D^ infected with recombinant lentivirus coexpressing Cre and CRISPR/CAS9 through nasal instillation. Lung tumor formation in KRAS^G12D^/GATA4-/- mice compared to KRAS^G12D^/TdTomato-/- mice 10 weeks post-infection. Scale = 200 μm. **h** Statistics of percentage of tumor area in the lung represented in **g** (n = 3 per group). **i** Schematics of intranasal instillation of retrovirus for forced expressing GATA4 in Dox inducible Tet-KRAS^G12C^/CC10rtTA lung cancer mouse model (referred to as KC). **j** Pathology of lung tumor formation in KC mice infected with control (mCherry) or GATA4 expressing virus. Scale = 200 μm. **k** Statistics of tumor area per lung area (n = 3 per group). Bars are represented as mean ± SEM of the indicated number (*n*) of repeats. *P < 0.05, **P < 0.01, and ***P < 0.001 by Student’s *t*-test

To systematically investigate the potential role of TFs in lung cancer, we individually transfected H23 cells, a lung cancer cell line harboring Kras^G12C^ mutation, with 1530 siRNA sets (each set containing four different siRNAs towards single genes) targeting TFs on a genome-wide scale. Through this screening measured by cell growth assay, we identified 23 siRNA sets which significantly promoted H23 cell growth (cutoff = 1.5) (Supplementary Figure 1A, Supplementary Data 1). Interestingly, RNA-Seq data analyses showed that these genes were downregulated in human lung cancer (Supplementary Data 2). We then plotted the cell growth rates against relative gene expression in clinical samples and identified five candidates with most dramatic effects (Fig. 1a; Supplementary Data 3), among which GATA4 stood out as the top hit. To further validate our screening results, we individually knockdown these five genes in another lung cancer cell line H460 and found that knockdown of GATA4, BTBD11 or EOMES significantly enhanced the colony formation in soft agar (Supplementary Figure 1B).

Although GATA4 inactivation has been frequently reported in human lung cancer^25–27^, its impact upon lung tumorigenesis and therapeutics remain largely unknown. We therefore focused on GATA4.Western blot analysis showed that GATA4 expression in human lung cancer cell lines including NCI-H226, NCI-H23, NCI-H460, PC9, and A549 cells was consistently lower than normal lung epithelial cell lines including HSEAC37, HSEAC2KT, and HBEC30 (Fig. 1b). Ectopic GATA4 expression significantly inhibited soft-agar colony formation in A549, PC-9, and H460 cells (Fig. 1c, d, Supplementary Figure 1C and 1D). We further established A549 stable cell line with doxycycline (Dox)-inducible GATA4 (referred to as A549i, Supplementary Figure 1E and 1F). We found that Dox treatment significantly inhibited soft-agar colony formation and decreased cell growth of A549i cells (Fig. 1e; Supplementary Figure 1G). Conversely, GATA4 knockdown dramatically enhanced the colony formation capabilities of H23 and H226 cell in soft agar (Fig. 1f, Supplementary Figure 1H, 1I, 1J and 1K). These data clearly supported the tumor suppressive function of GATA4 in vitro.

We went on to confirm tumor suppressor function of GATA4 in vivo using transgenic lung cancer mouse model. Previous study has established the simultaneous knockout of a target gene and activation of mutant KRAS in the lung epithelia in lsl-Kras^G12D^ transgenic mice using recombinant lentivirus coexpressing Cre and CRISPR/CAS9^28^. Following this protocol, we intranasally delivered lentiviruses targeting either TdTomato (serving as negative control) or GATA4 into lsl-Kras^G12D^ mice. In stark contrast to control group, we detected notable lung tumor nodules in 3 out of 4 mice post 10 weeks of lenti-sgGATA4 nasal inhalation (referred to as KG mice for **K**ras^G12D^/**G**ATA4-/-) (Fig. 1g, h, Supplementary Figure 1L), further supporting the tumor suppressive function of GATA4 in vivo.

We further constructed a retrovirus for Dox-inducible expression of GATA4 (mCherry serves as the negative control) in the lung compartment of TetO-Kras^G12C^/CC10rtTA bitransgenic mice (referred to as KC) (Supplementary Figure 1M and 1N). KC mice developed lung cancers with diffused bronchioloalveolar carcinoma (BAC) features after fed with a Dox diet for three weeks, whereas remained tumor-free and healthy when fed with normal diet^29^ (Supplementary Figure 1O). Retrovirus encoding TetO-GATA4 was delivered into the lung epithelial compartments through nasal instillation for Dox inducible expression as reported earlier^30^ (Fig. 1i). Reverse-transcription PCR analyses confirmed the ectopic GATA4 expression in lung tissues post 3 days of Dox treatment (Supplementary Figure 1P). As expected, the KC mice infected with control virus developed lung adenocarcinoma after 3 weeks of treatment with Dox (Fig. 1j). In contrast, retroviral expression of GATA4 significantly inhibited tumor formation (Fig. 1j, k). Similar result was obtained in TetO-EGFR T790M/Ex19del/CC10rtTA mice^31^ (Supplementary Figure 1Q and 1R).

### Ectopic expression of GATA4 induces lung cancer cell senescence

**Fig. 2:**
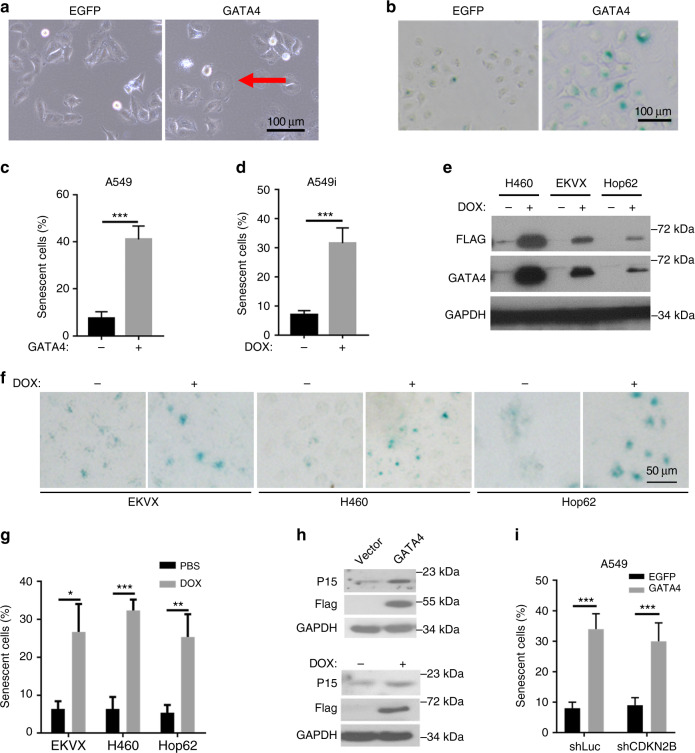
Ectopic GATA4 expression induces lung cancer cell senescence. **a** Microphotograph of A549 cells infected lentivirus overexpressing GATA4. Red arrow-head highlighted the senescent cells. Scale = 100 μm. **b** β-galactosidase staining of A549 cells infected with GATA4 or EGFP expressing lentivirus. Representative pictures were shown. Scale = 100 μm. **c**, **d** Statistics of β-Galactosidase staining of A549 cells ectopically expressing GATA4 through lentivirus infection (**c**) or Doxycycline treatment of A549i cells (**d**) (*n* = 3 per group). **e** Western blot analysis of Doxycycline-inducible GATA4 expression in stable cell lines of H460, EKVX, and Hop62. **f** β-Galactosidase staining of indicated cell lines before and after GATA4 expression. **g** Statistics of **f** (*n* = 3 per group). **h** Western blot analysis of p15INK4b expression in GATA4-expressing A549 cells. **i** β-galactosidase staining of GATA4-expressing A549 knockdown with control or CDKN2B-targeting shRNAs (*n* = 3 per group). Bars are represented as mean ± SEM of the indicated number (*n*) of repeats. *P < 0.05, **P < 0.01, and ***P < 0.001 by Student’s *t*-test

We next investigated how GATA4 influenced lung cancer cell growth. When GATA4 was overexpressed, A549 cells exhibited enlarged and flattened morphology, a typical feature of cell senescence (Fig. 2a). Indeed, cell senescence was confirmed through senescence-associated β-galactosidase staining (Fig. 2b, c). This senescent phenotype was also observed in Dox-treated A549i cells (Fig. 2d). Consistently, we found that significantly higher portion of cells were arrested at G_0/1_ when expressing GATA4 (Supplementary Figure 2A).

To confirm whether GATA4-induced senescence was common in lung cancer cells, we generated lung cancer cell lines (H460, EKVX, and HOP62) for Dox-inducible expression of GATA4 (Fig. 2e). We found that GATA4 expression significantly induced senescence (Fig. 2f, g).

Western blot analysis revealed that ectopic GATA4 expression led to the inhibition of pro-growth signals including phospho-ERK and phospho-AKT, but upregulation of PTEN, an anti-growth signal (Supplementary Figure 2B). Cellular senescence is a stress response that accompanies a stable exit from the cell cycle; it is commonly induced by cyclin-dependent kinase (CDK) inhibitors (CKI). Western blot analysis showed no significant change of p53, p21, p27, p14ARF, or p16INK4a level before and after GATA4 expression in A549 cells (Supplementary Figure 2C and 2D). Instead, p15INK4b, another CKI for eliciting senescence, showed notable increase after GATA4 expression (Fig. 2h). However, knockdown of p15INK4b expression with shRNAs failed to rescue cell from GATA4-induced senescence (Fig. 2i; Supplementary Figure 2E), suggesting that GATA4 induced senescence in lung cancer cells independently of canonical pathway.

### GATA4 induces cell senescence through downregulation of Wnt-7b signaling

As typical executors of senescence are not implicated in GATA4-induced senescence, we went on to determine the underlying signaling events. We ruled out two classical GATA4 transcriptional targets, NKX2.5 and SMARCD3^32^, as the effectors of GATA4-induced senescence (Supplementary Figure 3A-3F).

Recently, Elledge and colleagues reported that GATA4 elicited cellular senescence through NF-κB signaling pathway and featured senescence-associated secretory phenotype (SASP) in IMR90 fibroblast^33^. However, we did not detect obvious upregulation of SASP cytokines in lung cancer cells ectopically expressing GATA4 (Supplementary Figure 3G). Moreover, inhibition of NF-κB activity either through targeting *RELA* with shRNAs or treatment with inhibitor did not result in lung cancer cell senescence (Supplementary Figure 3H, 3I, 3J, 3K, and 3L), suggesting that NF-κB pathway is not involved in this process.

We then performed ChIP-seq analysis and found no typical senescence-related genes (Supplementary Data 4, 5 and 6). We further did RNA-seq and found that 302 genes were significantly upregulated (>2-fold change, *p* < 0.05, Supplementary Data 7) and 530 genes were downregulated (<0.5-fold change, *p* < 0.05, Supplementary Data 8) in cells with GATA4 expression induction. No specific pathway known to directly regulate senescence was identified in KEGG pathway analysis (Supplementary Data 9 and 10) or Gene Ontology enrichment analysis (Supplementary Data 11 and 12).

**Fig. 3:**
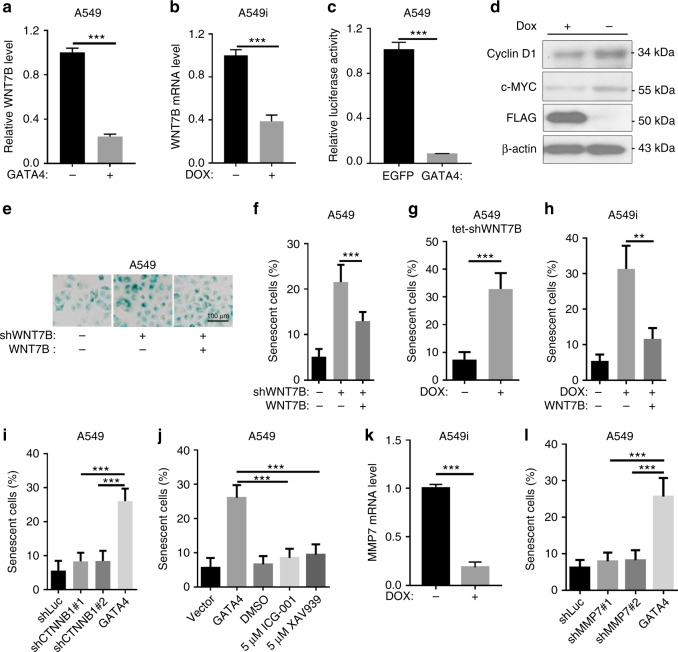
GATA4 induces lung cancer cell senescence by downregulating WNT7B. **a** and **b** WNT7B expression level in A549 cells ectopically expressing GATA4 through lentivirus infection (**a**) or Doxycycline treatment of A549i cells (**b**) (*n* = 3 per group). **c** TOP-FLASH analysis of GATA4 expressing A549 cells (*n* = 6 per group). **d** Western blot analysis of c-Myc and cyclin D1 in A549i cells in the absence or presence of 2 μg/mL of Dox. **e** β-Galactosidase staining of A549 cells expressing *WNT7B* targeting shRNA (middle panel) and rescued by shRNA-resistant *WNT7B* (right panel) (scale bar 100 μm). **f** Statistics of **e** (*n* = 3 per group). **g** β-Galactosidase staining of A549 expressing Dox-inducible shRNA targeting *WNT7B* (A549 tet-shWNT7B) in the absence or presence of 2 μg/mL of Dox (*n* = 3 per group). **h** β-Galactosidase staining of A549i in the absence or presence of 2 μg/mL of Dox and rescued by Wnt-7b expression (*n* = 3 per group). **i** Impact of shRNA targeting *CTNNB1* mRNA on induction of senescence in A549 cells (*n* = 3 per group). **j** β-Galactosidase staining of A549 cells treated with ICG-001 and XAV939 at indicated concentration (*n* = 3 per group). **k** qRT-PCR analysis of *MMP7* expression of in A549i cells in the absence or presence of 2 μg/mL of Dox (*n* = 3 per group). **l** β-Galactosidase staining of A549 cells knockdown with shRNA targeting *MMP7* mRNA (*n* = 3 per group). Bars are represented as mean ± SEM of the indicated number (*n*) of repeats. *P < 0.05, **P < 0.01, and ***P < 0.001 by Student’s *t*-test

Earlier reports demonstrated that the downregulation of Wnt signaling triggered senescence^34,35^. In our RNA-seq, we noticed that *WNT7B* expression level were downregulated in A549 cells ectopically expressing GATA4 (Supplementary Data 8). We suspected that GATA4-mediated downregulation of *WNT7B* may result in cell senescence. Real-time PCR analysis confirmed downregulation of *WNT7B* mRNA level in A549 cells with GATA4 expression (Fig. 3a, b). We found that GATA4 expression led to the downregulation of Wnt signaling, indicated by the decrease of β-Catenin transcriptional activity (Fig. 3c). Western analysis found that GATA4 expression led to the downregulation of Wnt pathway target genes including *c-Myc* and *CCND1* (Fig. 3d). Importantly, we found that knockdown of *WNT7B* was able to induce senescence in A549 cells (Fig. 3e), and this senescence was rescued by ectopic expression of shRNA-resistant *WNT7B* (Fig. 3e, f, Supplementary Figure 3M). We also generated A549 cells with Dox-inducible expression of shWnt7b (referred to as Tet-shWnt7b). Downregulation of *WNT7B* through Dox treatment in A549-Tet-shWnt7b resulted in senescence (Fig. 3g, Supplementary Figure 3N). Critically, GATA4-induced senescence can be rescued by *WNT7B* overexpression (Fig. 3h). These data identified Wnt-7b as the critical mediator of GATA4-induced senescence.

Wnt ligands were known to elicit a canonical signaling pathway (the β-catenin-dependent pathway), as well as non-canonical signaling pathways (β-catenin-independent); many Wnt ligands were found to elicit non-canonical pathways^36^. We asked whether the GATA4-induced senescence resulted from the downregulation of *WNT7B* was mediated by the canonical β-Catenin-dependent pathway. Knockdown of *CTNNB1* did not result in senescence (Fig. 3i; Supplementary Figure 3O). Likewise, treatments with β-Catenin inhibitors, XAV939^37^ and ICG-001^38^, respectively, did not lead to increased senescence (Fig. 3j; Supplementary Figure 3P), suggesting that canonical Wnt signaling is not responsible for GATA4-induced lung cancer cell senescence.

Downregulation of MMP7 expression is known to effectively induce cell senescence^39,40^. MMP7 is a transcriptional target of β-Catenin. Given that canonical Wnt signaling is not responsible for GATA4-induced senescence, MMP7 should not be involved in mediating GATA4-induced senescence. In our RNA-seq data, we found MMP7 expression was downregulated by GATA4 (Supplementary Data 8). We validated this downregulation in Dox-treated A549i cells through qRT-PCR (Fig. 3k). Importantly, knockdown of the *MMP7* did not increase senescence in A549 cells (Fig. 3l; Supplementary Figure 3Q). These results demonstrated that GATA4-induced senescence caused by downregulation of *WNT7B* is not mediated through the canonical Wnt signaling pathway.

Adams and colleagues have reported the mechanism underlying senescence elicited by downregulation of Wnt signaling: GSK3β downstream of WNT7B phosphorylates HIRA to drive translocation of HIRA to PML body to form complex with ASF1a, and thus initiating senescence process^34^. Since GATA4 induces lung cancer cell senescence through downregulating WNT7B, we checked PML-HIRA colocalization signal triggered by ectopic expression of GATA4. We detected robust PML-HIRA colocalization signal in A549 cells with WNT7B knockdown by shRNA in comparison to intact A549 cells (Supplementary Figure 3R). Interestingly, we found that GATA4 expression resulted in similar level of PML-HIRA colocalization to that found in WNT7B knockdown (Supplementary Figure 3R and 3S), suggesting that mechanism revealed by Adams and colleagues may apply to GATA4 induced senescence in lung cancer cells.

### TGF-β2 mediates GATA4-induced senescence upstream of Wnt-7b

**Fig. 4:**
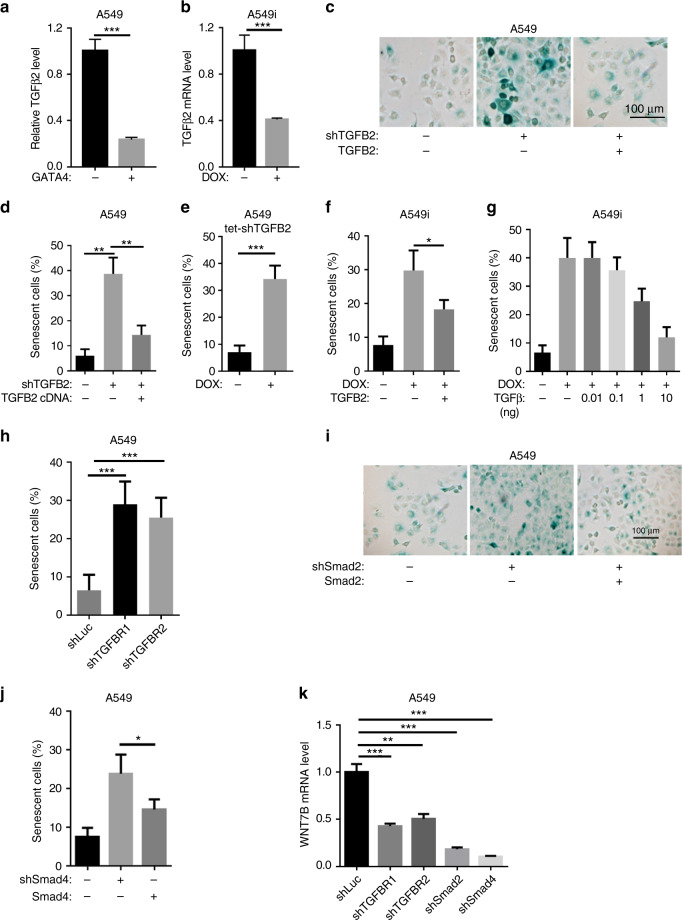
TGF-β2 mediates GATA4-induced senescence upstream of Wnt-7b. **a** and **b** qRT-PCR analysis of *TGFB2* expression in GATA4 overexpressing A549 cells through lentivirus infection (**a**) or Doxycycline treatment of A549i cells (**b**) (*n* = 3 per group). **c** β-Galactosidase staining of A549 cells with *TGFB2* knockdown (middle panel) and rescued by shRNA-resistant *TGFB2*. **d** Statistics of **c** (*n* = 3 per group). **e** β-Galactosidase staining of A549 expressing Dox-inducible shRNA targeting *TGFB2* (*n* = 3 per group). **f** β-Galactosidase staining of A549i in the absence or presence of 2 μg/mL of Dox and rescued by *TGFB2* expression (*n* = 3 per group). **g** β-Galactosidase staining of A549i cells in the absence or presence of 2 μg/mL of Dox and rescued with recombinant TGF-β protein at indicated concentration (*n* = 3 per group). **h** β-Galactosidase signal in A549 cells knockdown of *TGFBR1* and *TGFBR2* (*n* = 3 per group). **i** β-Galactosidase signal of A549 cells with *SMAD2* knockdown (middle panel) and rescued by shRNA-resistant *SMAD2* (right panel). **j** β-Galactosidase staining of A549 cells with *SMAD4* knockdown (middle panel) and rescued by shRNA-resistant *SMAD4* (right panel) (*n* = 3 per group). **k** *WNT7B* expression level in A549 cells with knockdown of *TGFBR1*, *TGFBR2*, *SMAD2*, or *SMAD4*, respectively (*n* = 3 per group). Bars are represented as mean ± SEM of the indicated number (*n*) of repeats. *P < 0.05, **P < 0.01, and ***P < 0.001 by Student’s *t*-test

We carefully analyzed ChIP-seq data and excluded the possibility of direct binding of GATA4 to *WNT7B* promoter (Supplementary Data 4). We then asked how GATA4 inhibited *WNT7B* expression. Our RNA-seq data analyses showed that TGF-β2 expression was downregulated in A549 cells expressing GATA4 (Fig. 4a, b). The TGF-β signaling pathway has been reported to be engaged in cross-talk with and upregulating the Wnt signaling^41,42^. We went on to test whether TGF-β2 is a critical mediator of GATA4-induced senescence. Interestingly, knockdown of *TGFB2* with shRNAs induced senescence (Fig. 4c). This senescence is effectively rescued by ectopic overexpression of shRNA-resistant *TGFB2* (Fig. 4c, d, Supplementary Figure 4A). This is also confirmed in A549 cells harboring Dox-inducible shRNA-targeting *TGFB2* (Fig. 4e, Supplementary Figure 4B). Importantly, we found that overexpression of *TGFB2* was able to rescue GATA4-induced senescence (Fig. 4f). We also supplemented Dox-treated A549i cells with recombinant TGF-β protein and found that TGF-β protein rescued GATA4-induced senescence of A549 cells in a dose-dependent manner (Fig. 4g), supporting that TGF-β protein itself prevented GATA4-induced senescence in lung cancer cells.

TGF-β2 binds to TGFBR2, which then forms a complex with TGFΒR1, and the complex then phosphorylates R-SMADs, enabling the formation of a complex with SMAD4 that can enter the nucleus to transcribe target genes^43^. We found that knockdown of either *TGFBR1* or *TGFBR2* promoted senescence in A549 cells (Fig. 4h; Supplementary Figure 4C). We also systemically tested the role of canonical SMADs including SMAD2, SMAD3, and SMAD4 in GATA4-induced senescence. We found that knockdown of either *SMAD2* or *SMAD4* increased senescence (Fig. 4i, j; Supplementary Figure 4D).

We went on to test whether TGF-β2 signaling controlled Wnt-7b expression. Interestingly, we found that knockdown of *TGFΒR1*, *TGFΒR2*, *SMAD2*, or *SMAD4* resulted in downregulation of *WNT7B* mRNA levels (Fig. 4k). Together, these data showed that TGF-β2 functioned upstream of Wnt-7b in mediating GATA4-induced senescence of lung cancer cells.

Of note, we found that GATA4 knockdown or overexpression did not significantly impact epithelial–mesenchymal transition of lung cancer cells in vitro and in vivo (supplementary Figure 4E, 4F, 4G, 4H, and 4I), consistent with earlier reports that TGFBR-SMAD signaling axis affect the senescence^44^.

### GATA4 downregulates the *TGFB2* mRNA level through miRNAs

**Fig. 5:**
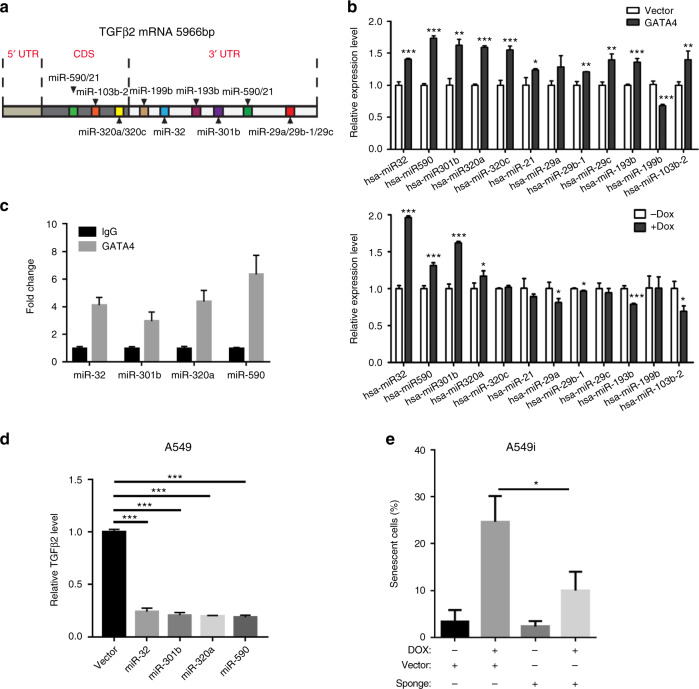
GATA4 downregulates the TGFB2 mRNA level through multiple miRNAs. **a** Schematics of targeting sites on *TGFB2* mRNA recognized by 14 miRNAs. **b** Relative expression level of miRNAs before and after GATA4 overexpression in A549i (through 2 μg/mL of Dox treatment) and A549 cells (through lentivirus infection) (*n* = 3 per group). **c** qPCR analysis of promoter fragment of designated gene from DNA samples prepared from control IgG or anti-FLAG (for pulldown GATA4) DNA samples. Quantity of GATA4 bond DNA was normalized by the value in control IgG-treated samples (*n* = 3 per group). **d** *TGFB2* mRNA level in A549 cells overexpressing miR-32, miR-301b, miR-320A, or miR-590, respectively (*n* = 3 per group). **e** β-Galactosidase signal in GATA4 overexpressing A549 cells in the absence or presence of microRNA sponge (*n* = 3 per group). Bars are represented as mean ± SEM of the indicated number (*n*) of repeats. *P < 0.05, **P < 0.01, and ***P < 0.001 by Student’s *t*-test

We did not detect GATA4 binding to the *TGFB2* promoter region in ChIP-seq analysis (Supplementary Data 4). In our ChIP-seq data we found promoter segments of 14 miRNA with potential to target *TGFB2* mRNA (Fig. 5a). The detailed predicted targeting properties of the mature miRNAs targeting *TGFB2* mRNA were listed in Supplementary Data 13. Four miRNAs (miRNA-32, miRNA-301b, miR-320a, and miR-590) were consistently upregulated in A549 cells expressing GATA4 (Fig. 5b). ChIP-PCR experiment showed the enrichment for the promoter regions of these four miRNA genes in the GATA4 immunoprecipitated DNA from GATA4 expressing A549 cells (Fig. 5c). Importantly, overexpression of each of these four miRNAs individually caused downregulation of the *TGFB2* mRNA level (Fig. 5d). Conversely, we blocked the function of these four miRNAs with a microRNA sponge construct^45^, and found this sponge construct efficiently rescued GATA4-induced senescence in A549 cells (Fig. 5e; Supplementary Figure 5A). These data clearly showed that miRNAs (miRNA-32, miRNA-301b, miR-320a, and miR-590) upregulated by GATA4 played an important role in mediating GATA4-induced senescence.

### GATA4-TGF-β2-Wnt-7b signaling axis is clinically relevant

**Fig. 6:**
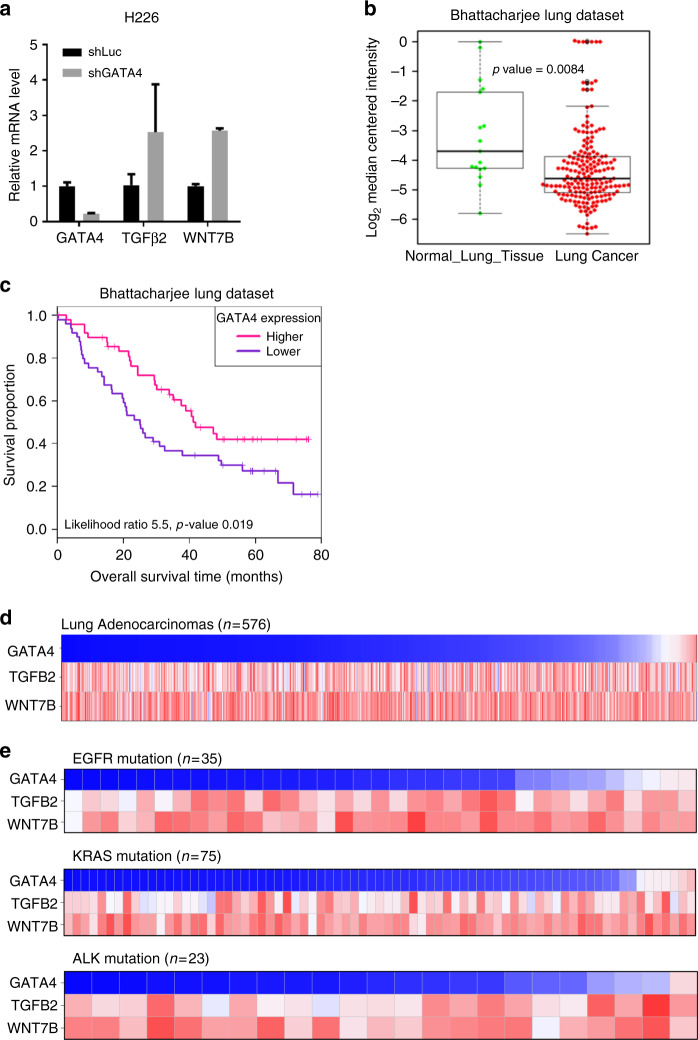
The GATA4-TGF-β2-Wnt-7b signaling axis is clinically relevant. **a** qRT-PCR analysis of *TGFB2* and *WNT7B* expression level in H226 cell line with GATA4 knockdown (*n* = 3 per group). **b** GATA4 expression level in lung cancer samples and normal lung tissues. **c** Kaplan–Meier survival curve of GATA4-high and GATA4-low lung cancer patients (Cox log-rank test, *p* = 0.019). **d** Reverse correlation of expression level of GATA4 versus TGF-β2 and Wnt-7b in clinical lung cancer samples downloaded from TCGA. **e** Expression pattern of GATA4 versus TGF-β2 and Wnt-7b in driver mutation positive samples. EGFR mutation positive: Upper panel; Kras mutation positive: middle panel; EML4-ALK mutation positive: lower panel

Our above data have shown that GATA4 expression downregulated *TGFB2* and the ensuing downstream *WNT7B* signaling in A549 lung cancer cell line. This was confirmed in H460 cells (Supplementary Figure 6A). Conversely, knockdown of GATA4 expression level upregulated *TGFB2* and *WNT7B* transcription in lung cancer cell line H226 (Fig. 6a), supporting that GATA4-TGF-β2-Wnt-7b signaling axis is common to lung cancer cells. We also found significant negative correlation of expression level between *GATA4* and *TGFB2* and between *GATA4* and *WNT7B* among non-manipulated lung cancer cell lines and normal lung epithelial cell lines (Supplementary Figure 6B).

In order to validate the clinical relevance of our findings, we searched database from Oncomine and found that the GATA4 mRNA level was significantly lower in lung cancer samples than paired normal tissues (Fig. 6b), consistent with earlier reports that the promoter region of GATA4 was methylated in 67% of lung cancer samples^25^. Moreover, a higher expression level of GATA4 was associated with longer overall survival in comparison to those with lower expression (Fig. 6c).

Our model predicts that GATA4 expression level is inversely correlated with those of TGFB2 and WNT7B in lung cancer. In order to find supporting evidence in clinical samples for our model, we searched the expression data for GATA4, TGFB2, and WNT7B in lung adenocarcinoma patients from The Cancer Genome Atlas (TCGA). We were able to download normalized gene expression profile from https://confluence.broadinstitute.org/display/GDAC/Download (lung adenocarcinoma, *n* = 576) and used log_2_ value to represent gene expression in each sample. In this system, an expression value of 0 means no expression; a value of 2 as very low expression; and 20 as very high expression.

With this dataset, we were able to check the relationship of expression between GATA4, TGFB2, and WNT7B. To our surprise, majority of adenocarcinoma samples presented low GATA4 expression and high *TGFB2* and *WNT7B* expression (Fig. 6d). Due to unbalanced sample distribution (not enough GATA4-high lung cancer samples), we were not able to calculate the significance in correlation test. However, this actually fitted our conclusion more favorably: GATA4 is inactive and at the same time WNT7B and TGFB2 is hyperactive in lung cancer cells.

Mutant EGFR, Kras, and EML4-ALK are critical driver oncogenes in lung cancers. We further checked pattern of expression of GATA4 versus TGFB2 and WNT7B in patients positive for mutations in these genes. We found that the pattern was highly similar in sub-cohorts of patients positive for mutation in EGFR, Kras and EML4-ALK respectively (Fig. 6e). Of note, we found very similar patterns in lung squamous cell carcinomas (Supplementary Figure 6C).

Taken together, these data suggest that GATA4 is frequently downregulated in lung cancers and the TGFB2-WNT7B signaling in these samples are activated.

### TGFBR1 is a potential target for GATA4-deficient lung cancer

Kras mutation positive lung cancer are resistant to therapies currently available in clinic. We went on to test our findings on this type of lung cancer. Our data has shown that TGFB2-WNT7 signaling axis is hyperactivated in GATA4-deficient lung cancer cells. We asked whether this axis can be employed for treating GATA4-low lung cancers. Potent inhibitors targeting TGFBRs are now under clinical trial for cancer therapy^46^. Among TGFBR inhibitors commercially available, SB525334 is most potent in inhibiting TGFBR-SMAD2 signaling and cell proliferation (Supplementary Figure 7A, 7B and 7C). Moreover, SB525334 at achievable serum concentration led to significant senescence^47^ (Supplementary Figure 7D and 7E).

**Fig. 7:**
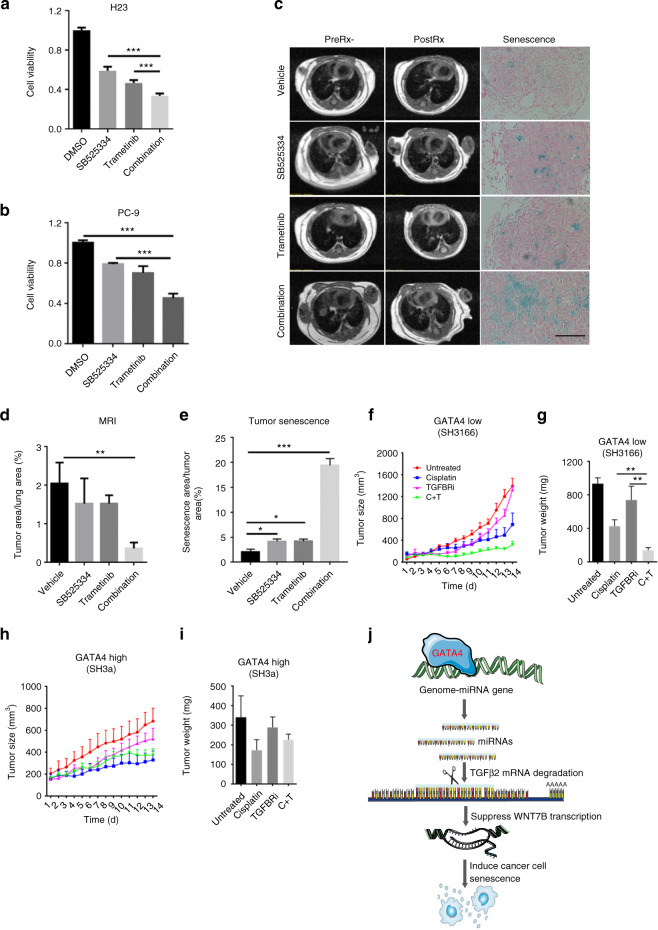
TGFBR1 is a potential target for GATA4-deficient lung cancer. **a** and **b** 5000 cells per well seeded in 96-well plates. Cells were treated with SB525334 (2 μM) and/or trametinib (2 μM). CCK8 value were checked 3 days after drug treatment. **a** For H23 and **b** for PC9 (*n* = 3 per group). **c** SB525334 (10 mg/kg/day) synergizes with trametinib (10 mg/kg/day) to shrink lung tumor in KRAS^G12D^/GATA4-/- mice. MRI image of lung of pre-treatment (left panel) and post-treatment (middle panel) of lung cancer bearing mice (PreRx and PostRx); β-Galactosidase staining of lung section of post-treatment mice (right panel). Scale = 100 μm. **d** Statistics of lung tumor burdens recorded in MRI (*n* = 4 per group). **e** Statistics of senescence in tumor shown (**c**) (*n* = 4 per group). **f**, **g**, **h** and **i** The tumor sizes and weights of PDX tumors treated with TGFBR inhibitor (SB525334, 10 mg/kg/day), Cisplatin (5 mg/kg, once a week), or combination. SH3166: GATA4-low PDX; SH3a: GATA4-high PDX (*n* = 6 per group). **j** Model of how GATA4 regulate TGF-β2 and Wnt-7b signaling and cellular senescence. See text for details. Bars are represented as mean ± SEM of the indicated number (*n*) of repeats. *P < 0.05, **P < 0.01, and ***P < 0.001 by Student’s *t*-test

We have previously shown that blocking MEK1/2 is partially effective in shrinking mutant Kras-driven lung cancer^48^. Interestingly, we found that SB525334 synergized with an FDA approved MEK1/2 inhibitor, Trametinib, to control H23 cell growth (Fig. 7a). Similar synergy was observed in PC-9 cells with EGFR mutation and low GATA4 expression (Fig. 7b, expression level of GATA4 in Fig. 1b). Although SB525334 showed no synergy with Trametinib in eliciting apoptosis, it did synergize with Trametinib in inducing senescence (Supplementary Figure 7F and 7G).

We went on to treat GATA4-deficient lung cancer through targeting TGFBR1 using transgenic mouse models. We generated a cohort of lung cancer-bearing mice through intranasal delivery of Lenti-sgGATA4 into lsl-Kras^G12D^ mice (referred to as KG mice, mentioned in Fig. 1g) and randomized them for treatments with SB525334, Trametinib, and SB525334/Trametinib combination for 2 weeks. We found that SB525334 treatment alone showed no significant treatment effect. However, Trametinib exhibited no significant treatment effect on KG mice, suggesting that GATA4 deficiency blunted therapeutic effect of MEK1/2 inhibition. Importantly, we found dramatic tumor shrinkage in mice treated with combined SB525334 and Trametinib treatment (Fig. 7c, d). Interestingly, we found that while either SB525334 or Trametinib elicited low level senescence in tumor tissues, combinational therapy elicited strong senescence signal (Fig. 7c–e).

To recapitulate the clinical setting, we tested treatment effect of TGFBR1 inhibitor on PDX mouse models. We collected surgically resected lung tumor nodules and inoculated them on nude mice. Western analysis showed both high and low GATA4 expression, respectively, in our samples (Supplementary Figure 7H). We found that Kras was wildtype in both GATA4-high (SH3a) and GATA4-low cancers (SH3166) (Supplementary Figure 7I and 7J). Consistent with our model, we found that SH3a had lower TGFB2 and Wnt7B expression level while SH3166 has higher TGFB2 and Wnt7B (Supplementary Figure 7K). As we did not expect Kras wildtype lung cancers responsive to Trametinib treatment, we chose to test whether SB525334 can synergize with cisplatin, the standard chemotherapeutics currently used in lung cancer clinic. We then grew these tumors in nude mice and treated them with vehicle, cisplatin, SB525334, or combination of cisplatin/SB525334. We found that cisplatin was partially effective in shrinking both GATA4-high and GATA4-low lung cancer PDX tumors, while SB525334 alone was not effective in either group (Fig. 7f–h). Importantly, while SB525334 did not obviously synergize with cisplatin in GATA4-high PDX samples (Fig. 7h, i), GATA4-low samples were highly sensitive to combinational treatment (Fig. 7f, g).

Taken together our data, we showed that *GATA4* is frequently inactivated in lung cancer cells. *GATA4* expression leads to upregulation of miRNAs (miRNA-32, miRNA-301b, miR-320a, and miR-590) targeting *TGFB2*, thus downregulating *TGFB2* and ensuing downstream *WNT7B* signaling pathway to trigger cell senescence. *GATA4* inactive lung cancers tend to have hyperactive *TGB2*-*WNT7B* signaling axis. Thereby, effective inhibitors targeting this signaling axis could potentially be employed for treating *GATA4*-deficient lung cancer patients. We have summarized our findings in Fig. 7j.

## Discussion

We here performed a genome-wide screening of TSG TFs in lung cancer and identified GATA4 as an important tumor suppressor. Ectopic GATA4 expression results in lung cancer cell senescence. Mechanistically, we showed that GATA4 upregulated multiple miRNAs to downregulate the level of *TGFB2* mRNA. TGF-β2 signals through TGFBR1, TGFBR2, SMAD2, and SMAD4 to upregulate *WNT7B* mRNA expression in lung cancer cells. By downregulating *TGFB2* level via miRNAs, GATA4 decreases expression of *WNT7B* to induce senescence in lung cancer cells. Tumor suppressive function of GATA4 was further confirmed in transgenic mouse models for lung cancer in vivo. Our findings were clinically relevant. Moreover, we showed that TGFBR1 inhibition could suppress the malignant progression of GATA4-deficient lung cancer.

The GATA zinc finger transcription factor family consists of six members (GATA1–6). Each member has two ZF and binds to the 5′-(A/T)GATA(A/G)-3′ DNA consensus sequence in genomic DNA^49^. Members of this family are involved in many biological activities, and dysfunctions related to these proteins have been found in human diseases^50^. GATA4 plays a critical role in both cardiac^49^ and lung development^24,51^. Mice with a heterozygous deletion mutation (exon 2 deletion) of *GATA4* show pulmonary defects including dilated distal airways and patches of thickened mesenchyme^51^. Frequent hypermethylation of the *GATA4* promoter in majority of lung cancer samples but not in normal human lung tissues has been reported^25–27^. However, the impact of *GATA4* silencing upon lung tumorigenesis and patients’ response to therapies remains largely unexploited. Our current work shed light on these important questions.

Stephen Elledge and colleagues have recently shown that GATA4 was stabilized by ATM and ATR to activate NF-κB and induce fibroblast senescence^33^. However, those critical features of senescence in that report were not detectable in our system. We reason this could be due to different tissue types and/or stress-specific conditions.

We further show that loss-of-function of GATA4 blunts cancer cells’ response to treatment of MEK_1/2_ inhibitors. It could be attributable to hyperactive TGFβ signaling in GATA4-deficient lung cancer. TGFβ signaling has been shown to confer drug resistance in lung cancer cells through activating MEK-ERK pathway^52^ or cancer stem cell property^53^. Both mechanisms as well as the activation of other potential oncogenes could blunt treatment effects of MEK_1/2_ inhibitors.

As GATA4 inactivation was reported in more than half of all clinical lung cancer cases, special attention should be paid to the status of GATA4 function for precision medicine for lung cancer patients. Phosphorylated TGFBRs, SMAD2, or SMAD4 could serve as biomarkers for the pathway activation. However, monitoring protein phosphorylation could be challenging in routine clinical practice, like immunohistochemistry (IHC), due to the time-lag in tumor sample collection during surgery and therein dephosphorylation of proteins. Moreover, IHC is less quantitative in nature, rendering it difficult to set the valuable standard. Other alternatives could be through monitoring mRNA expression of TGFβ or WNT7B.

TGF-β-signaling pathway plays a critical role in inducing regulatory T cells^54–57^. Senescent cancer cell recruits several types of immune effectors to the tumor region^58,59^. Inhibition of TGF-β-signaling pathway is, therefore, expected to enhance anti-tumor effect by activation of immune system^60–62^. Currently, plethora of inhibitors targeting the TGF-β-signaling pathway are in preclinical or clinical development^46^. Therapeutic intervention of TGF-β signaling should be considered as a choice for combination with current modalities^63^.

The TGFBR1 inhibitor, SB525334, efficiently inhibited lung cancer cell growth in vitro^47^. However, the in vivo effect was largely limited in transgenic lung cancer mouse model. We reason that several factors might be responsible for this discrepancy. First, lung epithelial cells must overcome tumor suppressing obstacles through mutation or silencing expression of tumor suppressor genes before they are transformed. As a result, the tumor nodules in autochthonous lung cancer mouse model are a complex mixture of highly heterogenous population of tumor cells in terms of gene mutation. Imaginably, there exist quite some clones that are de novo resistant to TGFBR1 inhibitor treatment. This could explain that while senescence was positive in a portion of cells in tumor nodules, the tumor shrinkage was limited. However, we cannot exclude the possibility that the relative insensitivity of in vivo tumor cells to SB525334 treatment was due to the difficulty of drug to reach tumor nodules. It has been reported that alteration of the function of vascular system and thereby the osmotic pressure within tumor nodules renders anticancer drug difficult to reach tumor tissues^64^, like that reported for mitomycin C^65^.

## Methods

### Animal care and use

All mice were housed in a pathogen-free environment in Jinan University and Institute of Biochemistry and Cell Biology, Shanghai Institutes for Biological Sciences, Chinese Academy of Sciences, respectively. All experimental protocols were approved by the Institutional Committee for Animal Care and Use at Jinan University, respectively, and Shanghai Institutes for Biological Sciences, respectively. All animal work was performed in accordance with the approved protocol. PDX treatment was carried out as we reported earlier^66^. The CC10-rtTA; TRE-Kras^G12C^ mice were sacrificed after 1 month of Dox diet feeding, while the CC10-rtTA; TRE-EGFR-TD mice were sacrificed after 3 months of feeding when the mice showed obvious panting phenotype. For IHC, mouse lung was inflated with 10% neutral buffered formalin solution (Sigma, St. Louis, MO, USA) and incubated overnight. Paraffin embedded lung tissue was cut into 5 μm slices for immunohistochemical staining.

For the single-dose virus delivery experiment, all the littermates of double transgenic mice were randomly divided into two groups of 3–4 mice. GATA4 or mCherry retroviruses were delivered to mouse lung via nostril inhalation. After 1 week of recovery, all the experimental mice were fed with Dox containing food until the day of sacrifice. Mouse lungs were collected for hematoxylin and eosin (H&E) staining. For quantification of tumor burden in the CC10-rtTA; TetO- Kras^G12C^ mice, we calculated the total size (mm^2^) of all the tumor regions in H&E sections under a microscope. For the CC10-rtTA; TetO-EGFR-TD mice, we counted the number of visible tumor nodules in the whole lung.

### Ethics statement

The protocol for human research was approved by Fudan University Shanghai Cancer Center. Written informed consent was obtained from every patient who donated tissues. All work was performed in accordance with the approved protocol.

### siRNA screen assay

Dharmacon siRNA reagent (#G-005805-01) was used. Reverse transfection method was used to deliver the siRNA to H23 cell^67^. Briefly, siRNA and lipid (Lipofectamine® 2000 Reagent) were mixed and added to 96-well plates. Then, H23 cells were added to the plates (10,000 cells per well). Cell proliferation rate was measured on day 3 by CCK8 assay. During screening, siRNAs targeting EGFP (5′-CGTGATCTTCACCGACAAGAT-3′) are included in the upper-most and lower-most rows of each plate, which served as negative control. The screening was conducted for one replica.

### Cell culture and cell engineering

A549, NCI-H460, NCI-H226, NCI-H23, EKVX, Hop62 were purchased from ATCC as part of NCI-60 (American Typical Culture Collection, Manassas, VA, USA). Beas-2b, PC-9, HEK293T, and Phoenix cells were kindly provided by Dr. Kwok-Kin Wong (The Helen and Martin Kimmel Center for Stem Cell Biology, NYU). Human bronchial epithelial cells (HBEC) and human small airway epithelial cells (HSAEC) cell lines were kindly gifted by Dr. John Minna from the University of Texas Southwestern Medical Center. Beas-2b, HEK293T, and Phoenix cells were cultured in DMEM plus 10% fetal bovine serum (FBS, Gibco, Life Technologies, Carlsbad, CA, USA). HBEC and HSAEC cells were cultured in SAGM medium (cc-3118, Lonza, Allendale, NJ, USA), and the rest of the NSCLC cell lines were cultured in RPMI-1640 with 10% FBS. To generate the Dox-inducible A549 (A549i) cell line, A549 cells were infected simultaneously with retrovirus encoding TetO-GATA4 (packaged from pREV-TRE-GATA4 vector) and retrovirus encoding EF1α-rtTA-iresGFP (packaged from pWPI-rtTA-iresGFP), then selected with 200 µg/mL Hygromycin (10687010, Invitrogen) for 2 weeks and FACS sorted for GFP-positive cells. For transient GATA4 expression, cell lines were infected with virus packaged from pWPI-GATA4 or control virus packaged from control construct pWPI-EGFP. For dox-induced knockdown of TGFβ2 or Wnt7b, pLKO-tet-puro vector harboring shRNA sequence targeting TGFβ2 or Wnt7b was packaged and infected A549 cells, then selected with 1 μg/mL puromycin (J593, Amresco) for 1 week. The lung cancer cell lines were authenticated by China Center for Type Culture Collection. All cell lines were maintained in mycoplasma-free environment by adding MYCO-3 (A5240,0020, AppliChem GmbH) and verified through PCR analysis (F: 5′-GGGAGCAAACAGGATTAGATACCCT-3′

R: 5′-TGCACCATCTGTCACTCTGTTAACCTC-3′).

To establish TGFβ2-expressing A549 stable cell line, HA-tagged TGFβ2-expressing DNA cassette CAG-TGFβ2-IRES-Neo was also electroporated (140 V, 25 ms) into A549 cell and selected with 700 µg/mL G418 (E859, Amresco) for 2 weeks. For shRNA knockdown, cells were infected with shRNA lentivirus (pLKO.1), selected with 2–3 µg/mL puromycin for 7–10 days. All cells were cultured in a 37 ^o^C humidified atmosphere containing 5% CO_2_.

### Plasmids

pCMV6-Entry-GATA4 (Myc-FLAG tagged) was purchased from Origene (RC210945, Rockville, MD, USA) and subcloned into lentivirial pWPI and retro-virial pREV-TRE vector, respectively. The pWPI plasmid was kindly gifted by Dr. Feng Shao at the National Institute of Biological Sciences (NIBS, Beijing, China). CAG-IRES-Neo plasmid was purchased from Addgene (deposited by Shinya Yamanaka, Addgene plasmid#13461) and the pREV-TRE retroviral plasmid (PT3215-5) and pTeton advanced plasmid (PT3899-5) were purchased from Clontech (Mountain View, CA, USA). Human TGFβ2 and CTNNB1 cDNA were purchased from NIBS Resource Center and then subcloned into CAG-IRES-Neo vector, respectively. Top-Flash plasmid was kindly gifted by Dr. Wei Wu in Tsinghua University. pSIN-DEST51-d2EGFP was kindly gifted by Dr. Yangming Wang in Peking University for construct microRNA sponge. All the following shRNAs were from the Biological Resource Center at NIBS, purchased from the Sigma Mission shRNA Library:

shGATA4#1(TRCN0000020424), shGATA4#2(TRCN0000329713),

shTGFB2(TRCN0000196326), shWnt7b(TRCN0000061877),

shTGFBR1(TRCN0000195626), shTGFBR2(TRCN0000040010),

shSMAD2(TRCN0000010477), shSMAD4(TRCN0000010323),

shBTBD11(TRCN0000136604), shDLX4(TRCN0000013776),

shEOMES (TRCN0000013175), shCDYL (TRCN0000276335),

shNkx2.5#1(TRCN0000013733), shNkx2.5#2(TRCN0000013734),

shSmarcd3#1(TRNC0000147806), shSmarcd3#2(TRCN0000179523),

shRelA#83 (TRCN0000014683), shRelA#86(TRCN0000014686),

shMMP7#1(TRCN0000304140), shMMP7#2(TRCN0000051843),

shCTNNB1#1(TRCN0000314991), shCTNNB1#2(TRCN0000314920),

shCDKN2B#1(TRCN0000038155), shCDKN2B#2(TRCN0000038156)

shCDKN2B#3(TRCN0000038157) and Luciferase shRNA (shLuc, SHC007) as a control shRNA.

### siRNA screen assay

Dharmacon siRNA reagent (#G-005805-01) was delivered into H23 cell through Reverse transfection method^67^. Briefly, siRNA and lipid (Lipofectamine® 2000 Reagent, 11668019) were mixed and added to 96-well plates. Then, H23 cells were added to the plates (10,000 cells per well). Cell proliferation rate was measured on day 3 by CCK8 assay (CK04-20, Dojindo Molecular Technologies, Inc.). During screening, siRNAs targeting EGFP (5′- CGTGATCTTCACCGACAAGAT-3′) are included in the upper-most and lower-most rows of each plate, which served as negative control. The screening was conducted once.

### Cell proliferation assay and Cell Titer-Glo assay

For cell proliferation assays, 50,000 A549i cells were seeded in each well of 12-well plates and cultured overnight. Dox was then added to a final concentration of 2 µg/mL. The cells were counted every other day.

For the Cell Titer-Glo assays, 5000 cells were seeded into each well of 96-well plates and cultured overnight, and the cells were cultured for another 48 h after adding Dox. Luminescence was measured using Cell Titer-Glo reagent (G5421, Promega, Madison, WI, USA) according to the manufacturer’s instructions. Doxycycline hyclate (D9891, Sigma, St. Louis, MO, USA) was dissolved in ddH_2_O (2 mg/mL) and stored at −80 °C.

### Soft-agar colony formation assay

Colony formation assays were performed in soft agar (0.6% lower gel and 0.35% upper gel) in six-well plates. A549 (10,000 cells), PC-9 (10,000 cells), H460 (10,000 cells), Beas-2b (10,000 Cells), NCI-H226 (10,000 cells), or NCI-H23 (10,000 cells) cells were seeded into each well of six-well plates. 2 µg/mL Dox was initially mixed with the upper gel, and 0.5 mL 1 × fluid media containing Dox was added to the surface of the upper gel every week. After 3–4 weeks, colonies were stained with 0.05% crystal violet. Colonies with the diameter over 200 µm were counted. The results are presented as mean values of triplicates.

### Virus packaging and concentration

Lentiviral plasmids were co-transfected with helping plasmids, psPAX2 and PMD2.G (deposited by Didier Trono, Addgene plasmid #12260 and #12259) into HEK293T cells using transfection reagent VigoFect (Vigorous Biotechnology, Beijing, China). The cells were washed with fresh medium 6–8 h post transfection and cultured in fresh media. After 48 h of culture, virus containing supernatant was collected. To package retrovirus, viral plasmid and helping plasmid pCL-Eco (deposited by Inder Verma, Addgene plasmid#12371) were co-transfected into Phoenix cells, and virus-containing medium was collected as described above. All the retroviruses used in the mouse experiments were condensed by the following procedure: virus containing medium was centrifuged at 27,500 rpm for 2 h. The supernatant was carefully removed and the virus particles were resuspended with 100 μL Opti-medium. Shaking gently at 4 ℃ overnight, the virus was prepared for use.

### RNA extraction and reverse-transcription PCR

Total RNA was extracted using Trizol reagent (15596-018, Invitrogen). 2 μg total RNA was reverse-transcribed to cDNA using the Takara M-MLV Reverse Transcriptase Kit (D2639B, Takara, Dalian, China). Human GAPDH gene was used as an internal control. Real-Time PCR was done by using an ABI 7500 Fast Real-Time PCR machine (Applied Biosystems, Life Technologies) and SYBR Premix Ex Taq II reagent (DRR820A, Takara). The following primers were used in the experiments:

TGFβ2-Forward: 5′-CTGTCTACCTGCAGCACACT-3′,

TGFβ2-Reverse: 5′- TGGGACTGTCTGGAGCACAA -3′,

Wnt7b-Forward: 5′- CGCAGCTATCAGAAGCCCAT-3′,

Wnt7b-Reverse: 5′- CAGGTGTTGCACTTGACGA-3′,

TGFBR1-Forward: 5′- CACAGAGTGGGAACAAAAAGGT-3′,

TGFBR1-Reverse: 5′- CCAATGGAACATCGTCGAGCA-3′,

TGFBR2-Forward: 5′- GTAGCTCTGATGAGTGCAATGAC-3′,

TGFBR2-Reverse: 5′- CAGATATGGCAACTCCCAGTG-3′,

SMAD4-Forward: 5′- ACGAACGAGTTGTATCACCTGG-3′,

SMAD4-Reverse: 5′- TGCACGATTACTTGGTGGATG-3′,

MMP7-Forward: 5′- ATGTGGAGTGCCAGATGTTGC-3′,

MMP7-Reverse: 5′- AGCAGTTCCCCATACAACTTTC-3′,

Gapdh-Forward:5′-GAAGGTGAAGGTCGGAGTC-3′,

Gapdh-Reverse:5′-GAAGATGGTGATGGGATTTC-3′

### Protein extraction and immunoblotting

Whole cell lysates were extracted by using the lysis buffer: 50 mM Tris pH 7.4, 150 mM NaCl, 1 mM EDTA, 1% Triton, and 10% glycerol along with protease and phosphatase inhibitor cocktail (4693132001& 4906837001, Roche, Basel, Switzerland) protein concentrations were determined by the Bradford assay. Soluble proteins (30–40 μg) were subjected to SDS–polyacrylamide gel electrophoresis. Separated proteins were electrophoretically transferred onto polyvinylidene difluoride (PVDF) membranes (Millipore, Billerica, MA, USA) and immunoblotted with anti-GATA4 (ab134057, 1:2000, Epitomics, Burlingame, CA, USA), -FLAG (F1804, 1:2000, Sigma), -CyclinD1 (2978S, 1:2000, CST, Danvers, MA, USA), -KRAS (12063-1-AP, 1:1000, Proteintech, Chicago, IL, USA), -Phos-ERK1/2 (4376S, 1:2000, CST), -Phos-AKT1 (3787S, 1:2000, CST), -ERK1/2 (4695S, 1:2000, CST), -AKT1 (2973S, 1:2000, CST), -PTEN (9188s, 1:1000 CST), -c-MYC (M4439, 1:2000, Sigma), -p21(2947S, 1:1000, CST), -p27(3686S, 1:1000, CST), -p53(2524S, 1:1000, CST), -p14/ARF(2407, 1:1000, CST), -p16/Ink4a (3562-1, 1:1000, Epitomics), -p15 (YT3492, 1:1000, ImmunoWay, Newark, DE, USA) or β-actin (A5316, 1:2000, Sigma) antibody. Immunoreactive proteins were visualized using ECL Western Blotting Substrate (PREGENE, Beijing, China) and X-ray films.

### ARMS-PCR

Genomic DNAs were extracted from SH3166 and SH3a. ARMS-PCR was conducted on DNA samples with the primers listed below: G12A: ATA TAA ACT TGT GGT AGT TGG AGC TTC; G12C: AAT ATA AAC TTG TGG TAG TTG GAG CCT; G12D: ATA TAA ACT TGT GGT AGT TGG AGC GGA; G12R: AAT ATA AAC TTG TGG TAG TTG GAG CTC; G12S: AAT ATA AAC TTG TGG TAG TTG GAG CG A: G12V: ATA TAA ACT TGT GGT AGT TGG AGC AGT; Reverse: ATG CAC AGA GAG TGA ACA TCA TGG AC; WT: AAT ATA AAC TTG TGG TAG TTG GAG CTG.

### Senescence associated β-Galactosidase (SA-β-Gal) staining

SA-β-Gal staining was performed using the Cell Signaling Senescence β-Galactosidase Staining Kit (CST #9860). Briefly, 20,0000–30,0000 cells were seeded in each well of six-well plates and cultured until the time of staining. For Dox treatment, a final concentration of 2 μg/mL was used and Dox containing medium was changed every other day. Wnt pathway inhibitors, ICG-001 (S2662), XAV939 (S2619), were purchased from Selleckchem (Houston, TX, USA) and were all dissolved in DMSO. Chemicals were added to the A549 cells (DMSO < 0.1%) for 4 days treatment and then underwent SA-β-Gal staining to see their effect in senescence.

For quantification of SA-β-Gal-positive cells elicited by GATA4 expression, blue positive cells in at least six randomly selected fields at ×200 magnification under an inverted microscope were counted. For shRNA knockdown cells, we calculated the percentage of SA-β-Gal cells in the three picked observed fields. The β-Gal staining for each group was experimentally repeated three times.

For SA-β-Gal staining of lungs tissues, kras^G12D^;GATA4-/- mice treated with inhibitors as indicated were fixed, washed with PBS/NP-40, and incubated in staining solution for β-galactosidase. Stained lungs were fixed 4% paraformaldehyde and embedded in paraffin. Section of 5 μm were stained with fast nuclear red. Senescence signaling was counted on cells stained blue.

### Top-Flash assay

500 ng Top-Flash plasmid mixed with 200 ng CAG-CTNNB1-IRES-Neo plasmid and 500 ng pCMV6-GATA4 plasmid or 500 ng Empty Vector was transfected to A549 cell seeded in 12-well plate via VigoFect reagent. Both the treatment had three replicates. After 48 h culture, A549 cells were trypsinized and counted. 100 μL culture medium within 20,000 A549 cells was mixed with 100 μL Firefly Luciferase reagent (E1910, Promega, Madison, WI, USA) to measure the value of luminescence. For evaluation the effect of Wnt pathway inhibitors in Top-Flash assay, three inhibitors were added to the A549 cells post 24 h transfection for another 48 h incubation until the day of measurement.

### microRNA assay

Total RNA of A549 cell was extracted by Trizol as described previously. 1 μg total RNA was reverse transcribed microRNAs via one step polyA-tailed assay in the following 25 μL mixture: 25 mM dNTP (2.5 μL), 100 mM ATP (2.5 μL), 2.5 mM MnCl_2_ (2.5 μL), 10 × *E. coli* PolyA Polymerase buffer (2.5 μL), QmiR-RT primer (5′- GCGAGCACAGAATTAATACGACTCACTATAGGTTTTTTTTTTTTTTTTTTVN-3′,500 ng), RNase Inhibitor (0.5 μL), M-MLV Reverse Transcriptase (1 μL) (Takara) and *E. coli* PolyA Polymerase (1 μL) (NEB, Ipswich, MA, USA). The reaction was run at 37 ℃ for 90 min and inactivated at 95 ℃ for 10 min. The following microRNAs primers were used in the Real-Time PCR and human U6 was an internal control.

qmiR-Reverse: 5′-GCGAGCACAGAATTAATACGAC-3′

hsa-miR-590-Forward: 5′-CGGCGGTAATTTTATGTATAAGCTAG-3′

hsa-miR-320c-1-Forward: 5′-GCGAAAAGCTGGGTTGAGAGGG-3′

hsa-miR-320a-Forward: 5′-TCGGAAAAGCTGGGTTGAGAGGGC-3′

hsa-miR-32-Forward: 5′-CGGCG TATTGCACATTACTAAGT-3′

hsa-miR-301b-Forward: 5′-CGGCGGCAGTGCAATGATATTGTC-3′

hsa-miR-29c-Forward: 5′-CGCCGTAGCACCATTTGAAATCGG-3′

hsa-miR-29b-1-Forward: 5′-CGGCGGTAGCACCATTTGAAATC-3′

hsa-miR-29a-Forward: 5′-TCGGTAGCACCATCTGAAATCGG-3′

hsa-miR-21-Forward: 5′-GCCGCTAGCTTATCAGACTGATG-3′

hsa-miR-199b-Forward: 5′-TCGGCCCAGTGTTTAGACTATCTG-3′

hsa-miR-193b-Forward: 5′-CGGGGTTTTGAGGGCGAGATGA-3′

hsa-miR-103b-2-Forward: 5′-TCATAGCCCTGTACAATGCTGC-3′

U6-Forward: 5′-CTCGCTTCGGCAGCACA-3′

U6-Reverse: 5′-AACGCTTCACGAATTTGCGT-3′

Four microRNA precursors were subcloned into the pLenti6 lenti-vrial vector (kindly gifted by Dr. Zhiqian Zhang at Beijing Cancer Hospital) and 10 μg/mL blasticidin (15205, Invitrogen) was used to select microRNA-expressing A549 cells. The following pre-sequences of four microRNAs were used in the experiment.

hsa-miR-590: 5′-TAGCCAGTCAGAAATGAGCTTATTCATAAAAGTGCAGTATGGTGAAGTCAATCTGTAATTTTATGTATAAGCTAGTCTCTGATTGAAACATGCAGCA-3′

hsa-miR-320a: 5′-GCTTCGCTCCCCTCCGCCTTCTCTTCCCGGTTCTTCCCGGAGTCGGGAAAAGCTGGGTTGAGAGGGCGAAAAAGGATAGGT-3′

hsa-miR-32: 5′-GGAGATATTGCACATTACTAAGTTGCATGTTGTCACGGCCTCAATGCAATTTAGTGTGTGTGATATTTTC-3′

hsa-miR-301b: 5′-GCCGCAGGTGCTCTGACGAGGTTGCACTACTGTGCTCTGAGAAGCAGTGCAATGATATTGTCAAAGCATCTGGGACCA-3′

To block mature microRNAs, miRNA sponges targeting hsa-miR-590, hsa-miR-320a, hsa-miR-32 and hsa-miR-301b was inserted into pSIN-DEST51-d2EGFP. The reverse and forward DNA (detailed below) was annealed:

miRNA-Sponge-F: TCGCCCTCTCGGTCAGCTTTTCCAGTGCAACTTAGCGCGTGCAATACCAGCTGCACTTTTCCCATAAGCTCCCAGGCTTTGACAAGAGATTGCACTGCCAGATCG

miRNA-Sponge-R: CTGGCAGTGCAATCTCTTGTCAAAGCCTGGGAGCTTATGGGAAAAGTGCAGCTGGTATTGCACGCGCTAAGTTGCACTGGAAAAGCTGACCGAGAGGGCGACGAT

### Chromatin immunoprecipitation (ChIP)-Seq

ChIP was performed following lab protocol. Briefly, A549i cells were seeded in eight 150 mm plates, Dox was added to a final concentration of 2 µg/mL when the cells reached a confluence of 30–40%. After 4 days of culture, the cells were crosslinked with 1% formaldehyde (final concentration) and sonicated using the following parameters: 5 s on, 10 s off, 15 cycles at the 25% set power (VCX500, SONICS, CT, USA). The total cell lysate was divided into two aliquots, one was mixed with 40 μL of 1 mg/mL GATA4 antibody (6H10, Thermo Fisher Scientific, Waltham, MA, USA) and 40 μL Protein A/G Agarose beads (20423, Pierce, Thermo Scientific), and the other was mixed with 40 μL 1 mg/mL normal mouse IgG (NI03, Sigma) and 40 μL Agarose beads. After overnight incubation, the beads were washed with ChIP washing buffer. Chromatin was eluted and the crosslink was reversed and DNA was extracted with phenol/chloroform. Finally, the eluted DNA was resolved in ddH_2_O and processed to NIBS Sequencing Center for further high through-put seq. The result can be downloaded at http://www.ncbi.nlm.nih.gov/geo/query/acc.cgi?acc = GSE85003

### Mouse treatment

GATA4 gRNAs were designed, validated, and inserted in pSECC vector (generously provided by F.J. Sanchez-Rivera and T. Jacks, Koch Institute for Integrative Cancer Research at MIT). Lentivirus virus was packaged from pSECC-sgGATA4-infected 293T cells, validated through cell infection and administered nasally into lsl-Kras^G12D^ mice. After tumor burden was confirmed by MRI imaging 12 weeks after virus infection, mice were treated with vehicle solution, SB525334 (S1476, Selleckchem) 10 mg/kg/day, Trametinib (a gift from Qingsong Liu at High Magnetic Field Laboratory, Chinese Academy of Sciences) 10 mg/kg/day and the combination of two drugs. Lung tumor was documented again 2 weeks after drug dosing. The ratio of tumor size to chest size was calculated to quantify tumor burden. The tumor burden is calculated by area of tumors per area of lung.

For PDX assay, SH3166 or SH3a PDX was embedded to nude mice. Mice were treated with TGFBR inhibitor (SB525334, 10 mg/kg/day), Cisplatin (S1166, Selleckchem) (5 mg/kg, once a week) or combination as the size of xenograft between 100 and 200 mm^3^. Two weeks after treatment, mice sacrificed. The tumor volume is calculated by the formula: volume = (width)^2^*length*0.5.

### RNA-sequencing

We used four tumor/paratumoral tissue pairs of lung cancer patients: a 57-year-old female, a 60-year-old female, a 51-year-old male, and a 47-year-old male adenocarcinoma patient, respectively. Tumor and adjacent tissues were ground into powder in liquid nitrogen; total RNA was harvested using RNeasy Mini Kit (74104, QIAGEN); then total RNA samples were treated with DNase I and the mRNA is enriched by using the oligo(dT) magnetic beads.

RNA libraries were prepared for sequencing using standard Illumina protocols. Illumina Casava1.8 software used for base calling. Sequenced clean reads were mapped to Homo sapiens (hg19) whole genome using tophat v1.4.1 with parameters -i 10 -I 80000 --solexa1.3-quals --min-coverage-intron 20 --max-coverage-intron 11000 --min-segment-intron 10 --max-segment-intron 81000 --segment-length 20.

Gene annotation and calculation of FPKM values was carried out using Cufflinks (v2.0.2) with the provision of a GTF annotation file (hg19). Gene expression differences were assessed by Cuffdiff use upper-quartile normalization, with false discovery rate correction for multiple testing. The sequencing result can be downloaded at https://www.ncbi.nlm.nih.gov/geo/query/acc.cgi?acc=GSE84852.

### Statistic analysis

All the data were presented as mean values ± standard error of the mean. Differences between experimental groups were compared with unpaired two-tailed *t*-test. Statistical analyses were performed with GraphPad Prism 5.0, *p* < 0.05 was deemed to be statistically significant.

### Reporting summary

Further information on experimental design is available in the Nature Research Reporting Summary linked to this article.

## Supplementary information

Supplementary Information

Description of Additional Supplementary Files

Supplementary Data 1

Supplementary Data 2

Supplementary Data 3

Supplementary Data 4

Supplementary Data 5

Supplementary Data 6

Supplementary Data 7

Supplementary Data 8

Supplementary Data 9

Supplementary Data 10

Supplementary Data 11

Supplementary Data 12

Supplementary Data 13

Reporting Summary

## Source data

Source Data

## Data Availability

All the data referenced in the manuscript can be downloaded from websites indicated in the Methods section. Chromatin immunoprecipitation (ChIP)-Seq data for GATA4-binding sites in lung cancer can be downloaded from http://www.ncbi.nlm.nih.gov/geo/query/acc.cgi?acc=GSE85003. RNA-seq data for tumor and paratumoral tissues data can be downloaded at https://www.ncbi.nlm.nih.gov/geo/query/acc.cgi?acc=GSE84852.
