# Author Correction: Lung cancer deficient in the tumor suppressor GATA4 is sensitive to TGFBR1 inhibition

**DOI:** 10.1038/s41467-025-63723-5

**Published:** 2025-09-18

**Authors:** Lei Gao, Yong Hu, Yahui Tian, Zhenzhen Fan, Kun Wang, Hongdan Li, Qian Zhou, Guandi Zeng, Xin Hu, Lei Yu, Shiyu Zhou, Xinyuan Tong, Hsinyi Huang, Haiquan Chen, Qingsong Liu, Wanting Liu, Gong Zhang, Musheng Zeng, Guangbiao Zhou, Qingyu He, Hongbin Ji, Liang Chen

**Affiliations:** 1 Key Laboratory of Functional Protein Research of Guangdong Higher Education, Institute of Life and Health Engineering, College of Life Science and Technology, Jinan University, 510632 Guangzhou, China; 2 College of Life Sciences, Beijing Normal University, 100875 Beijing, China; 3 College of Biological Sciences, China Agricultural University, 100094 Beijing, China; 4 Key Laboratory of Molecular Imaging, Institute of Automation, Chinese Academy of Sciences, 100190 Beijing, China; 5 The University of Texas Health Science Center at Houston (UTHealth), 2450 Holcombe Blvd., Suite 1, Houston, TX 77021 USA; 6 Beijing Tongren Hospital, Capital Medical University, 100730 Beijing, China; 7 State Key Laboratory of Cell Biology, Shanghai Institutes for Biological Sciences, Chinese Academy of Sciences, 200031 Shanghai, China; 8 CAS Center for Excellence in Molecular Cell Science, Shanghai Institutes for Biological Sciences, Chinese Academy of Sciences, 200031 Shanghai, China; 9 Innovation Center for Cell Signaling Network, Institute of Biochemistry and Cell Biology, Shanghai Institutes for Biological Sciences, Chinese Academy of Sciences, 200031 Shanghai, China; 10 University of Chinese Academy of Sciences, Beijing, China; 11 Department of Thoracic Surgery, Fudan University Shanghai Cancer Center, 200032 Shanghai, China; 12 High Magnetic Field Laboratory, Chinese Academy of Sciences, 230031 Hefei, Anhui China; 13 Department of Experimental Research, Sun Yat-sen University Cancer Center, Guangzhou, China; 14 State Key Laboratory of Molecular Oncology, National Cancer Center/Cancer Hospital, Chinese Academy of Medical Sciences and Peking Union Medical College, 100021 Beijing, China; 15 School of Life Science and Technology, Shanghai Tech University, 200120 Shanghai, China

Correction to: *Nature Communications* 10.1038/s41467-019-09295-7, published online 10 April 2019

In the version of the article initially published there were errors in Fig. 4i. The two images of A549 (shSmad2: – Smad2: –, shSmad2: + Smad2: +) in Fig. 4i were incorrect duplicates from the images of A549 (shTGFB2:+ TGFB2:+, shTGFB2:– TGFB2: –), respectively, in Fig. 4c. Figure 1 below serves to update Fig. 4 in the article. The raw data for Fig. 4c and 4i are included as Supplementary Information alongside this amendment.

**Fig. 1:**
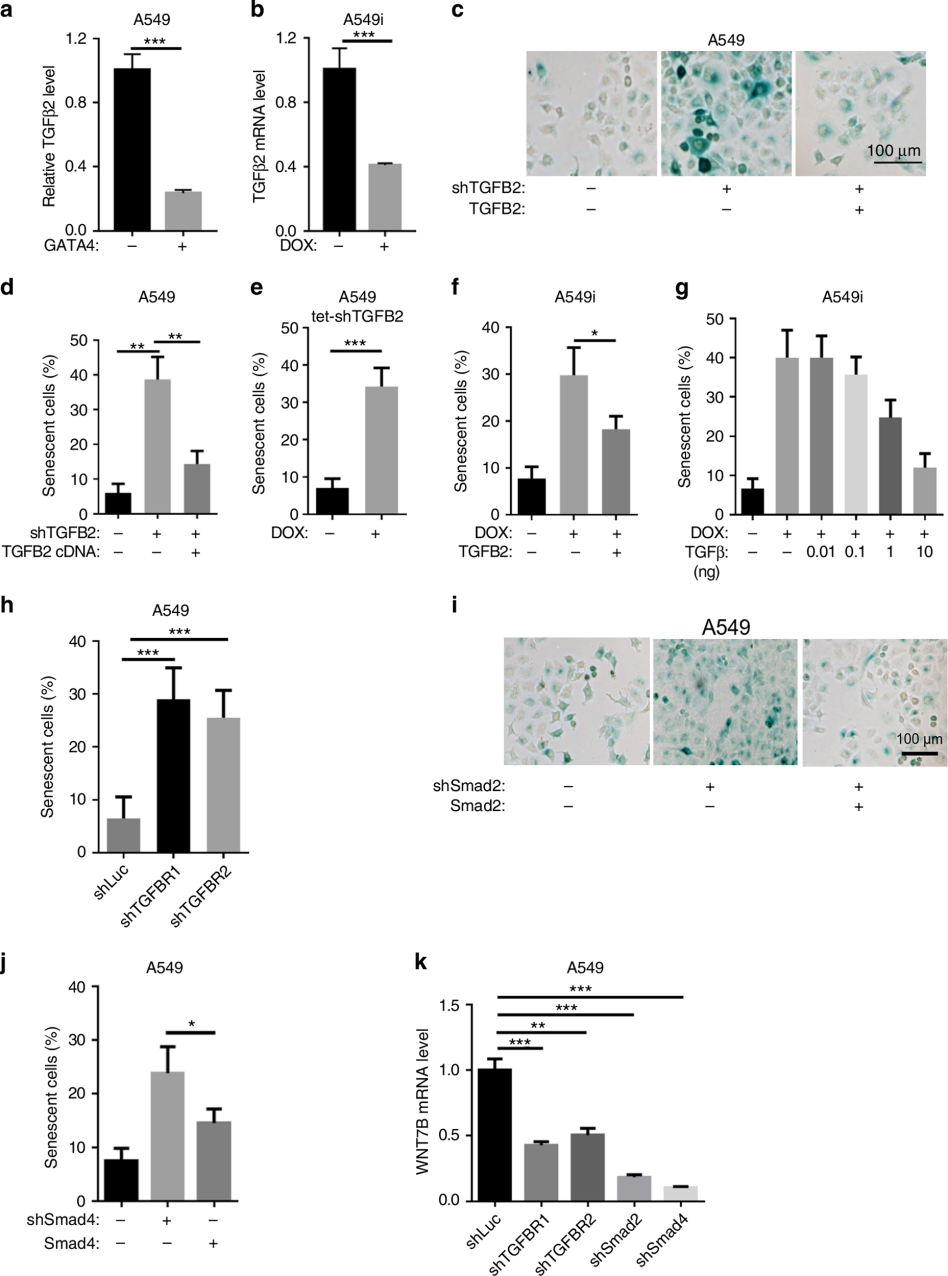
Revised Fig. 4.

## Supplementary information

Figure 4c,i raw data

