# Supplementary material for: Lung cancer deficient in the tumor suppressor GATA4 is sensitive to TGFBR1 inhibition
Source: Nat Commun. 2019 Apr 10;10:1665. doi: 10.1038/s41467-019-09295-7 (PMC6458308; doi:10.1038/s41467-019-09295-7)
Supplement: Supplementary file 1 — Supplementary Information [file 41467_2019_9295_MOESM1_ESM.pdf]

Supplementary Information

Lung Cancer Deficient in the Tumor Suppressor GATA4 are Sensitive To TGFBR1 Inhibition

Lei Gao[1,2*], Yong Hu[1*], Yahui Tian[1*], Zhenzhen Fan[1,3 *], Kun Wang[4*], Hongdan Li[1], Qian Zhou[1], Guandi Zeng[1], Xin Hu[5], Lei Yu[6], Shiyu Zhou[7,8,9], Xinyuan Tong[7,8,9,10], Hsinyi Huang[7,8,9], Haiquan Chen[11], Qingsong liu[12], Wanting Liu[1], Gong Zhang[1], Musheng Zeng[13], Guangbiao Zhou[14], Qingyu He[1#], Hongbin Ji[7,8,9,15#], Liang Chen[1#]

[1] Key Laboratory of Functional Protein Research of Guangdong Higher Education, Institute of Life and Health Engineering, College of Life Science and Technology, Jinan University, Guangzhou 510632, China.

[2] College of Life Sciences, Beijing Normal University, Beijing, 100875, China.

[3] College of Biological Sciences, China Agricultural University, Beijing 100094, China

[4] Key Laboratory of Molecular Imaging, Institute of Automation, Chinese Academy of Sciences, Beijing, 100190, China

[5] The University of Texas Health Science Center at Houston (UTHealth), 2450 Holcombe Blvd., Suite 1, Houston, TX 77021

[6] Beijing Tongren Hospital, Capital Medical University, Beijing 100730, China

[7] State Key Laboratory of Cell Biology, [8] CAS Center for Excellence in Molecular Cell Science, [9]Innovation Center for Cell Signaling Network, Institute of Biochemistry and Cell Biology, Shanghai Institutes for Biological Sciences, Chinese Academy of Sciences, Shanghai 200031, China.

[10] University of Chinese Academy of sciences, Beijing, China.

[11] Department of Thoracic Surgery, Fudan University Shanghai Cancer Center, Shanghai 200032, China.

[12] High Magnetic Field Laboratory, Chinese Academy of Sciences, Hefei 230031, Anhui, P. R. China

[13] Department of Experimental Research, Sun Yat-sen University Cancer Center, Guangzhou, China

[14] State Key Laboratory of Molecular Oncology, National Cancer Center/Cancer Hospital, Chinese Academy of Medical Sciences and Peking Union Medical College, Beijing 100021, China

[15]School of Life Science and Technology, Shanghai Tech University, Shanghai, 200120, China.

* These authors contributed equally to this work.

#Corresponding authors:

Liang Chen,

Key Laboratory of Functional Protein Research of Guangdong Higher Education, Institute of Life and Health Engineering, College of Life Science and Technology, Jinan University, Guangzhou 510632, China.

E-mail: chenliang@jnu.edu.cn

Phone: 86-20-8522-3139

Fax: 86-20-8522-7039

Hongbin Ji,

State Key Laboratory of Cell Biology, CAS Center for Excellence in Molecular Cell Science,

Innovation Center for Cell Signaling Network, Institute of Biochemistry and Cell Biology, Shanghai Institutes for Biological Sciences, Chinese Academy of Sciences, Shanghai 200031, China

E-mail: hbji@sibcb.ac.cn

Qingyu He

Key Laboratory of Functional Protein Research of Guangdong Higher Education, Institute of Life and Health Engineering, College of Life Science and Technology, Jinan University, Guangzhou 510632, China.

E-mail: tqyhe@email.jnu.edu.cn

Authors declare no conflict of interest.

Running title: lung tumor suppressor impact on cancer treatment

Supplementary Figure 1

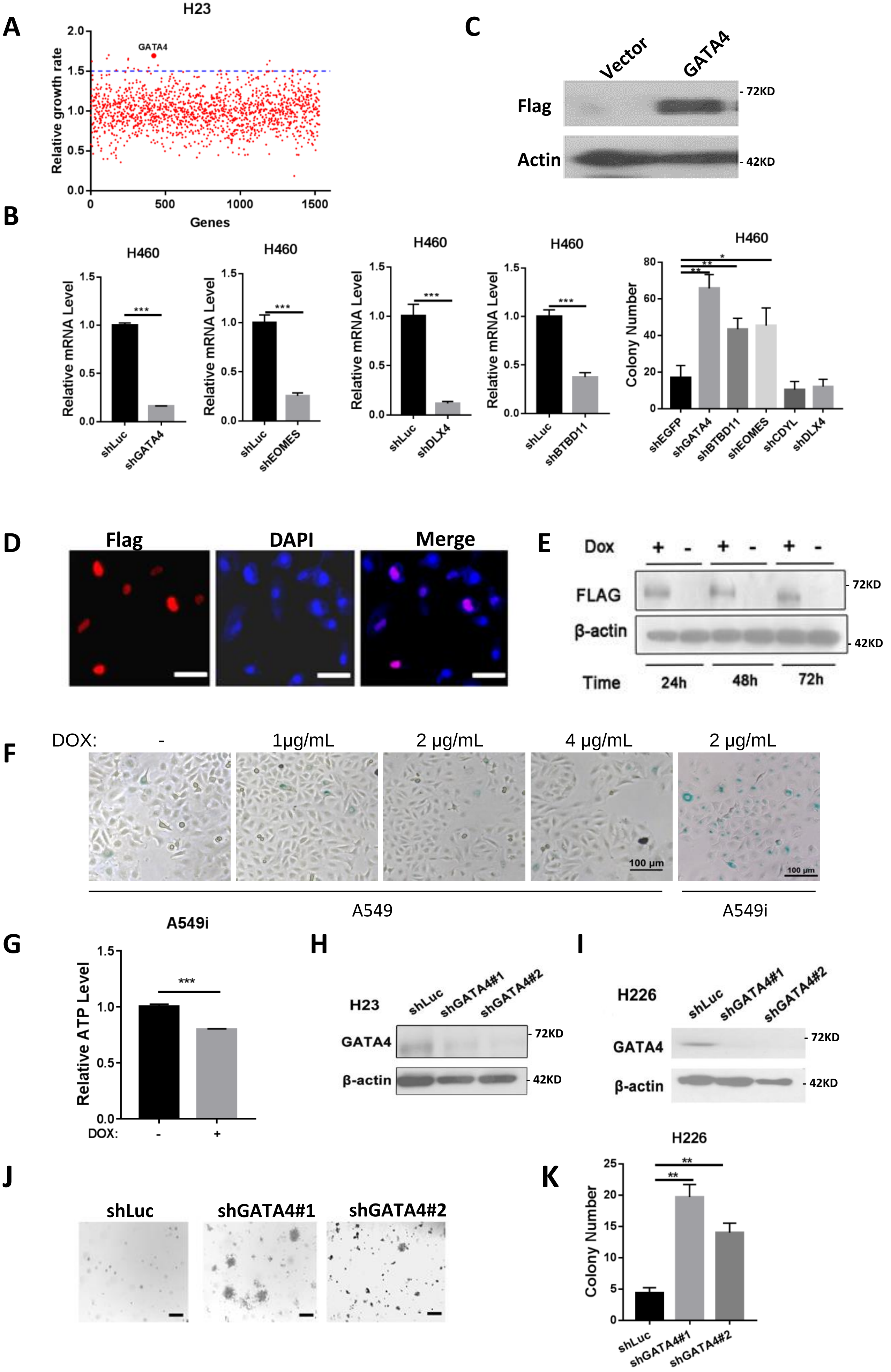

L

GATA4 gRNA1	Gata4_281_0	TAGAGGCCACAGGCATTGCA CAG
GATA4 gRNA2	Gata4_282_0	AGAGGCCACAGGCATTGCAC AGG
GATA4 gRNA3	Gata4_280_0	CATCTTGTGATAGAGGCCAC AGG

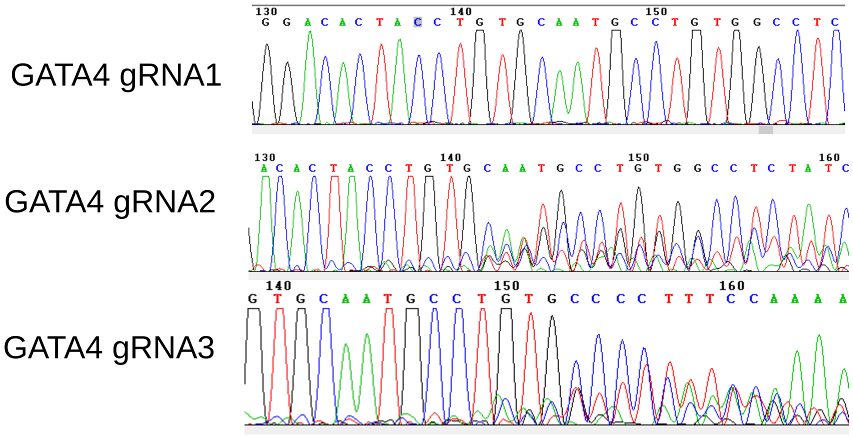

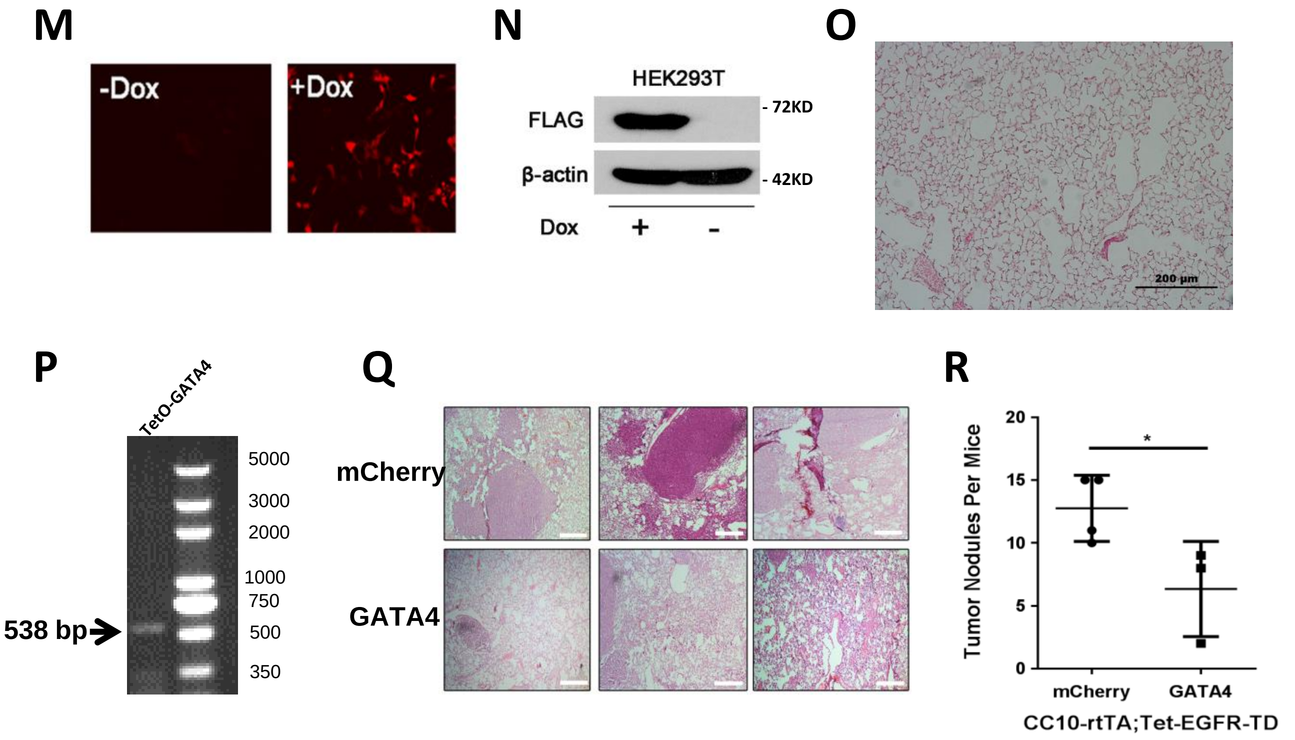

Figure legends for supplementary figures:

Supplementary Figure 1. GATA4 is a functional tumor suppressor in lung cancer.

A. Relative growth rate determined through CCK8 assay on siRNA set transfected H23 cells normalized by control siRNA (targeting EGFP) transfected H23 cells. **B.** H460 cells infected shRNA lentivirus targeted GATA4, BTBD11, EOMES, CDYL or DLX4 were selected and soft agar colony formation was performed. Left panel: shRNA knockdown efficiency. Right panel: Soft-agar colony number of H460 cell with indicated gene knockdown (n=3 per group). **C.** A549 cell infected with lentivirus for expressing FLAG tagged GATA4 and selected with 1 μg per ml of puromycin for 4 days. Western blot analysis with anti-FLAG antibody were shown. **D.** A549 cells infected with lentivirus for expressing FLAG tagged GATA4. Cells were stained with PE-conjugated anti-FLAG antibody and DAPI. Confocal microscopy used to show nuclear localization of GATA4 proteins. **E.** Stable cell line harboring Dox inducible FLAG tagged GATA4 (A549i) were treated with or without 2 μg per ml of Dox for 24, 48, and 72 hours. Western blot analysis with anti-FLAG antibody revealed inducible expression of GATA4 in A549i. **F.** A549 cells treated with Dox (0, 1, 2, 4 μg per mL) for 48 hours and β-galactosidase staining results are shown. Results indicate Dox is well tolerated by lung cancer cells up to 4 μg per mL. Senescence is not affected in lung cancer cells by Dox treated at indicated doses. A549i served as a positive control for senescence staining. **G.** 5,000 A549i cells seeded in 96-well plate and culture in media with or without 2 μg per ml of Dox for 72 hours. ATP contents in these cells were measured with Cell Titer-Glo Cell Titer-Glo assay (n=6 per group). **H.** H23 cells infected with lentivirus encoding shRNA targeting GATA4 or luciferase (serving as negative control). Cells selected with 1 μg per ml of puromycin for 4 days. Western blotting analysis of survived cells showing shRNA

knockdown effect on GATA4 expression. **I.** H226 cells infected with lentivirus encoding shRNA targeting GATA4 or luciferase (serving as negative control). Cells selected with 1 μg per ml of puromycin for 4 days. Western blotting analysis of survived cells showing shRNA knockdown effect on GATA4 expression. **J.** 10,000 of the above H226 cells (**I**) were seeded in soft-agar plate for 14 days. Colony numbers were counted. **K.** Statistics of result represented in **J** (n=3 per group). **L.** The efficiency of GATA4 sgRNA. Upper panel: sgRNA used for targeting GATA4. Numbering of sgRNA, targeting position, and sequence information is shown in the table. Lower panel: DNA sequencing chromatogram showing In/Del mutations detected in GATA4 genomic DNA in sgRNA transfected cells. **M.** Fluorescent microphotography of 2 μg per ml Dox untreated or treated 293T cells transfected with construct encoding TetO-mCherry segment and CMV-rtTA. **N.** Western blot analysis of doxycycline treated 293T cells transfected with construct encoding TetO-GATA4(FLAG) and CMV-rtTA. Western analysis with Anti-FLAG antibody were shown. **O.** H & E staining of TetO-Kras G12C/CC10rtTA bitransgenic mice fed on normal diet at age of 4 month. **P.** RT-PCR analysis of lung of 3-day doxycycline diet treated CC10rtTA mice intranasally infected with lentivirus encoding TetO-GATA4. **Q & R.** Lung cancer tumor burden in CC10rtTA/TetO-EGFR-T790M/Del19 infected with retrovirus encoding TetO-mCherry or TetO-GATA4. Pathological examination of lung compartment of 3 mice each were shown in **Q**. Statistics of number of tumor nodules were shown in **R** (n=3 per group). Bars are represented as mean ± SEM of the indicated number (n) of repeats. $*P < 0.05$, $**P < 0.01$, and $***P < 0.001$ by Students' t-test.

Supplementary Figure 2

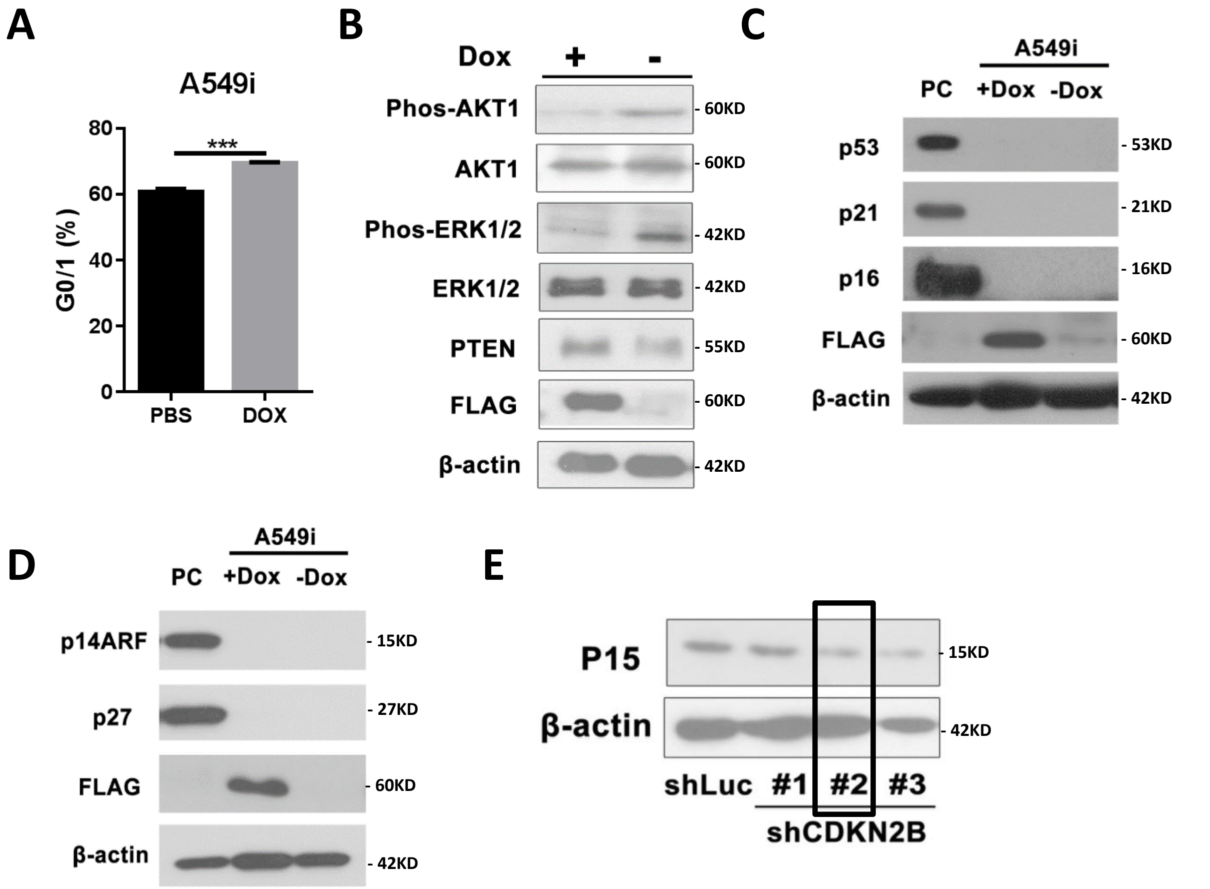

Supplementary Figure 2. GATA4 induced senescence independent of canonical senescent pathway.

A. A549i cells treated with or without DOX for 48 hours and PI staining for cell cycle analysis (n=3 per group). **B.** Western blot analysis of signaling protein for pro- and anti-growth in A549i cells treated with or without 2 μg per ml of Dox for 72 hours. **C&D.** Western blot analysis of Cyclin-dependent kinase inhibitors in A549i cell lines treated with or without 2 μg per ml of Dox for 72 hours. Positive control (PC) is 293T cells transiently transfected with plasmid encoding the corresponding gene. **E.** shRNA knockdown of p15INK4b expression in A549 cells.

Supplementary Figure 3

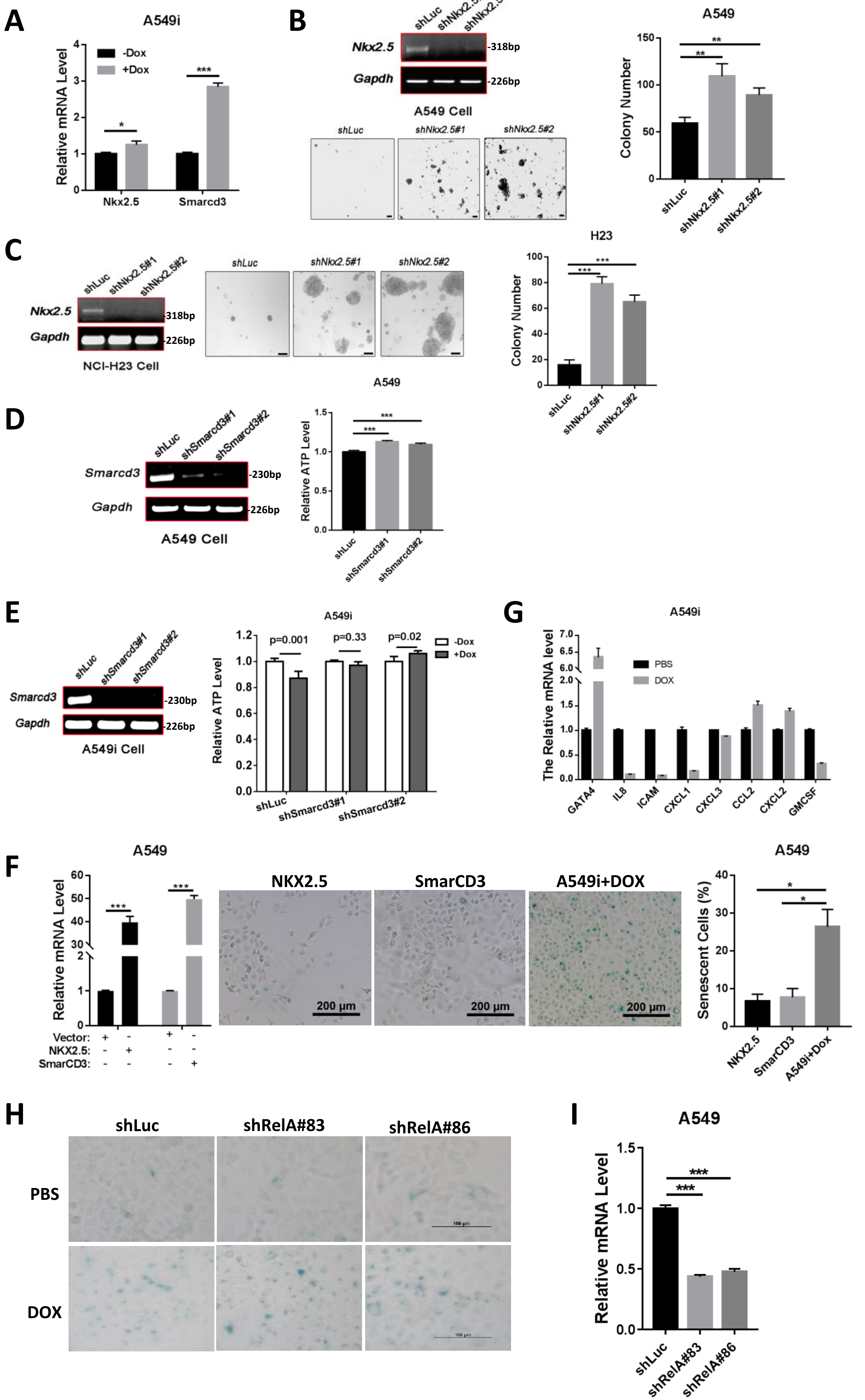

Supplementary Figure 3

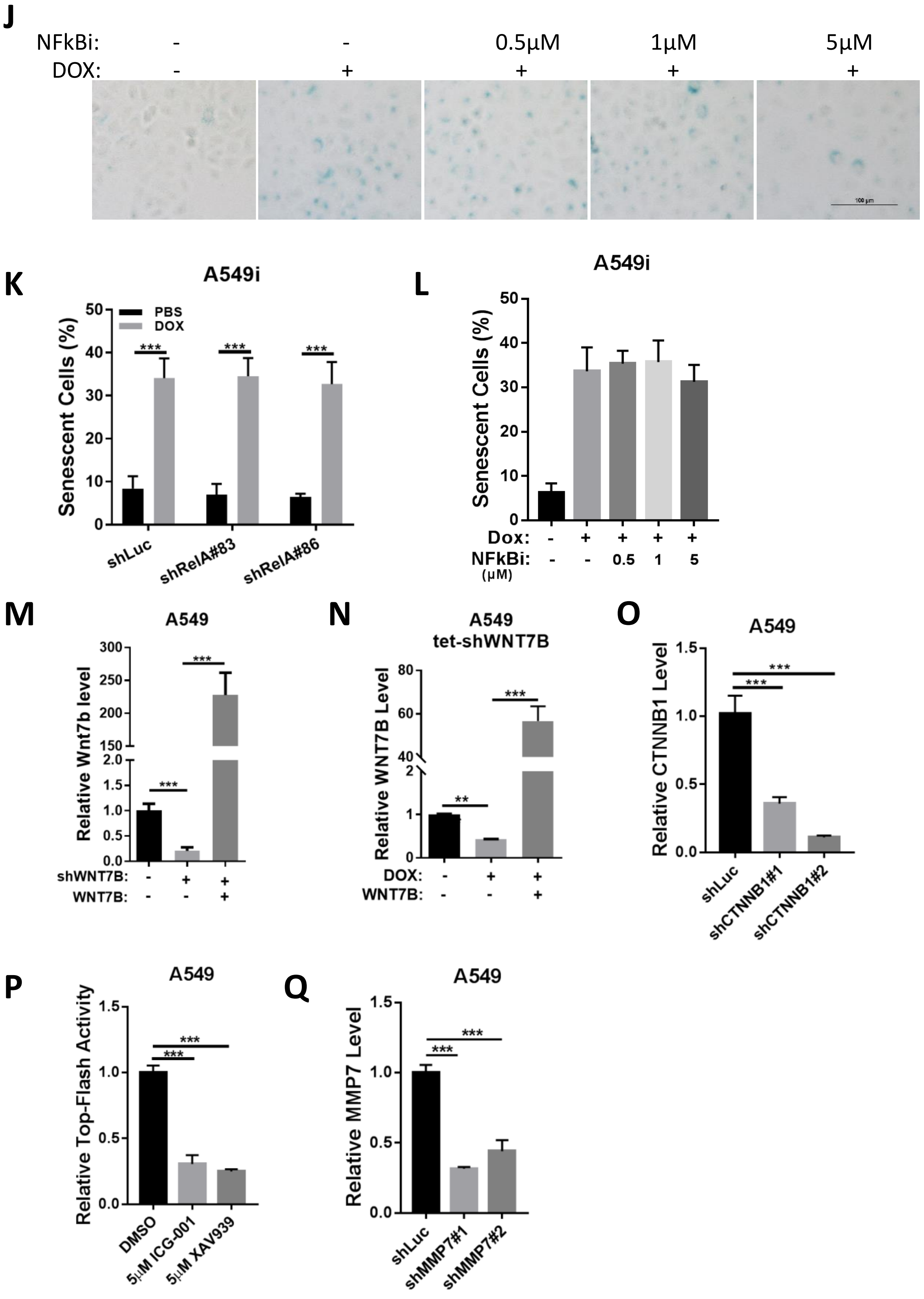

Supplementary Figure 3

R

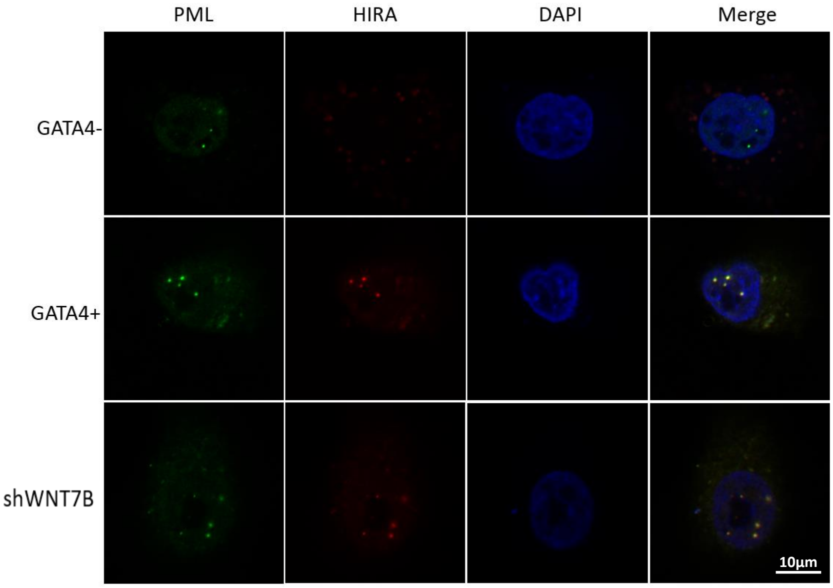

S

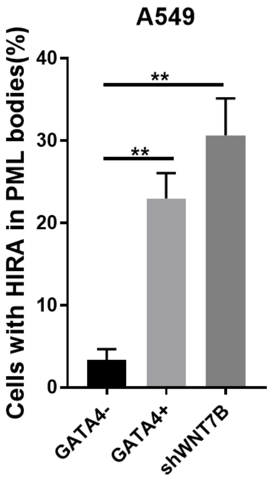

Supplementary Figure 3

A. qRT-PCR analysis of Nkx2.5 and Smarcd3 expression in A549i cell lines treated with or without 2 μg per ml of Dox for 72 hours (n=3 per group). **B.** Colony forming ability of A549 cell line infected with shRNA against NKX2.5 mRNA. Upper left panel: qRT-PCR analysis of NKX2.5 mRNA level in A549 cell line infected with lentivirus encoding shRNA targeting NKX2.5 mRNA. Lower left panel: Soft-agar colony formation ability of A549 cells with knockdown of NKX2.5. Right panel: statistics of the result represented in lower left panel (n=3 per group). **C.** Colony forming ability of H23 cell line infected with lentivirus encoding shRNA targeting NKX2.5 mRNA. Left panel: qRT-PCR analysis of NXK2.5 mRNA level in H23 cell line knockdown with shRNA targeting NKX2.5 mRNA. Middle panel: Soft-agar colony formation ability of H23 cells with knockdown of NKX2.5. Right panel: statistics of the result represented in middle panel (n=3 per group). **D.** Colony forming ability of A549 cell line infected with lentivirus encoding shRNA targeting SMARCD3 mRNA. Left panel: qRT-PCR analysis of SMARCD3 mRNA level in A549 cells. Right panel: Cell Titer-Glo assay of A549 cells with knockdown of SMARCD3 (n=3 per group). **E.** SMARCD3 knockdown partially rescues cell growth inhibition caused by ectopic expression of GATA4. Left panel: qRT-PCR analysis of SMARCD3 mRNA level in A549i cell line infected with shRNA targeting SMARCD3 mRNA. Right panel: viable cells measured by ATP level in A549i cells with SMARCD3 knockdown in the presence or absence of 2 μg per ml of Dox for 72 hours (n=6 per group). **F.** NKX2.5 or SMARCD3 overexpression didn’t result in lung cancer cell senescence. A549 cells infected with Lentivirus overexpressing NKX2.5 or SmarCD3 respectively and selected with 1μg per ml of puromycin for one week. A549 cells overexpression NKX2.5 or SMARCD3 stained negative of β-Galactosidase

activity. Dox treated A549i served as positive control. Left panel: qRT-PCR quantification of NKX2.5 and SMARCD3 expression in A549. Middle panel: β-galactosidase staining of A549 overexpressing NKX2.5 and SMARCD3 respectively. Right panel: statistics of senescent signal (n=3 per group). **G.** The relative mRNA level of SASP genes in A549i cells treated with or without 2 μg per ml of Dox for 72 hours (n=3 per group). **H.** β-galactosidase staining of A549i with the knockdown of RelA in the presence or absence of 2 μg per ml of Dox. **I.** Knockdown efficiency of shRNA targeting RelA mRNA (n=3 per group). **J.** β-galactosidase staining of A549i cells treated with NF-κB inhibitor BMS-345541 at indicated concentration in the presence or absence of 2 μg per ml of Dox. **K.** Statistics of **H** (n=3 per group). **L.** Statistics of **J** (n=3 per group). **M.** Knockdown and rescue efficiency of shRNA targeting WNT7B mRNA (n=3 per group). **N.** Knockdown and rescue efficiency of WNT7B expression in A549 cells harboring DOX-inducible shRNA targeting WNT7B (n=3 per group). **O.** Knockdown efficiency of shRNA targeting CTNNB1 mRNA (n=3 per group). **P.** TOP-FLASH result of A549 cells treated with β-Catenin inhibitors, ICG-001 and XAV939 (n=3 per group). **Q.** Knockdown efficiency of shRNA targeting MMP7 mRNA in A549 cells (n=3 per group). **R.** A549 cells were transfected with pRosa-HIRA and GATA4 or Wnt7b shRNA. Three days later, cells were fixed, tritonX-100 treated, and stained with anti-PML (sc-377390, Santa Cruz, mouse anti-human PML antibody) and anti-HIRA (ab216718, Abcam; Alexa Fluor 594 conjugated rabbit anti-human HIRA antibody) antibodies. FITC conjugated goat anti-mouse IgG secondary antibody was applied to visualize PML. Colocalization signal were captured with confocal microphotography (scale bar 10 μm). **S.** Statistics of **R** (n=3 per group). Bars are represented as mean ± SEM of the indicated number (n) of repeats. $^{*}P < 0.05$, $^{**}P < 0.01$, and $^{***}P < 0.001$ by Students' t-test.

Supplementary Figure 4

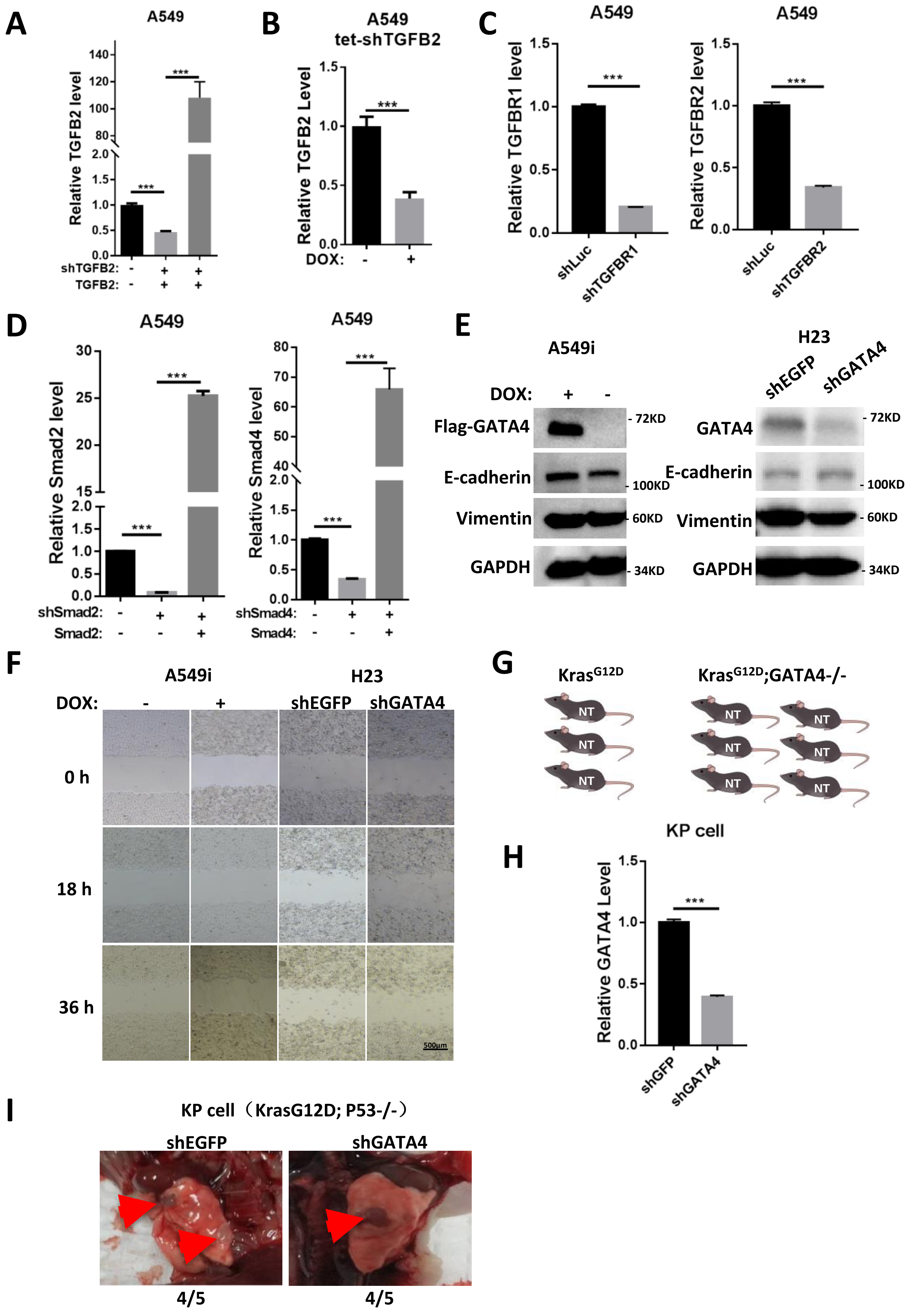

Supplementary Figure 4

A. qRT-PCR quantification of TGFB2 mRNA expression in A549 cells, A549 cells infected with lentivirus encoding shRNAs targeting TGFB2, and A549 with gene knockdown and infected with lentivirus overexpressing shRNA-resistant TGFB2 cDNA. Result showing high knockdown and rescue efficiency of shRNA targeting TGFβ2. **B.** Knockdown efficiency of DOX-inducible shRNA targeting TGFβ2 in A549 cells. **C.** Knockdown efficiency of TGFBR1 and TGFBR2 in A549 cells. **D.** qRT-PCR quantification of TGFB2 mRNA expression in A549 cells, A549 cells infected with lentivirus encoding shRNAs targeting indicated genes, and A549 with gene knockdown and infected with lentivirus overexpressing shRNA-resistant cDNA. Result showing high knockdown and rescue efficiency of shRNA targeting Smad2 and Smad4 respectively. **E.** Western blot analysis of expression level of E-cadherin and Vimentin in A549 expressing GATA4 or H23 cells with GATA4 knockdown. **F.** Wound healing analysis of in A549 expressing GATA4 or H23 cells with GATA4 knockdown (scale bar 500 μm). **G.** No metastatic tumor nodules found in important organs of mice 12 months after Cre virus treatment for $Kras^{G12D}$ (n = 3) and $Kras^{G12D}$;GATA4-/- (n = 6) genotypes. **H.** KP (KrasG12D;P53-/-) cells were infected by GATA4 shRNA virus and selected by puromycin for one week. Total mRNA was extracted and GATA4 expression level was validated. **I.** 2×10^6 KP cell with shRNA targeting EGFP or GATA4 were injected subcutaneously into the lower flank of the mice. 2 months later, mice were sacrificed and 4 out of 5 mice in each group were verified to have lung tumor metastasis. Red arrowhead highlighting the metastatic tumor nodules. Bars are represented as mean ± SEM of 3 repeats. **P* < 0.05, ***P* < 0.01, and ****P* < 0.001 by Students' t-test.

A

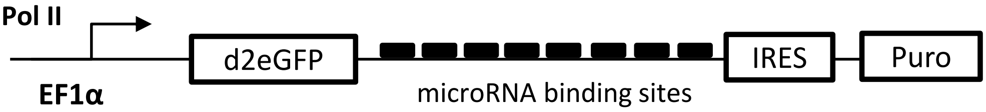


```
                     UUGG
3'-AGCGGGAGAG              GUCGAAAA-5'   hsa-miR-320a
Sponge 5'-TCGCCCTCTC  GGT  CAGCTTTT-3'

                     AUUA
3'-ACGUUGAAUC              CACGUUAU-5'   hsa-miR-32-5p
Sponge 5'-TGCAACTTAG  CGC  GTGCAATA-3'

                     UACU
3'-GACGUGAAAA              UAUUCGAG-5'   hsa-miR-590-5p
Sponge 5'-CTGCACTTTT  CCC  ATAAGCTC-3'

                     AUAG
3'-CGAAACUGUU              UAACGUGAC-5'  hsa-miR-301b-3p
Sponge 5'-GCTTTGACAA  GAG  ATTGCACTG-3'
```

Supplementary Figure 5A. Schematics of miRNA sponge. Sequence of absorbing repeats is shown for each miRNA. The sponge construct contains 2 absorbing repeats for each of miRNA (miRNA-32, miRNA-301b, miR-320a, and miR-590). Bars are represented as mean ± SEM of 3 repeats. *$P < 0.05$, **$P < 0.01$, and ***$P < 0.001$ by Students' t-test.

Supplementary Figure 6

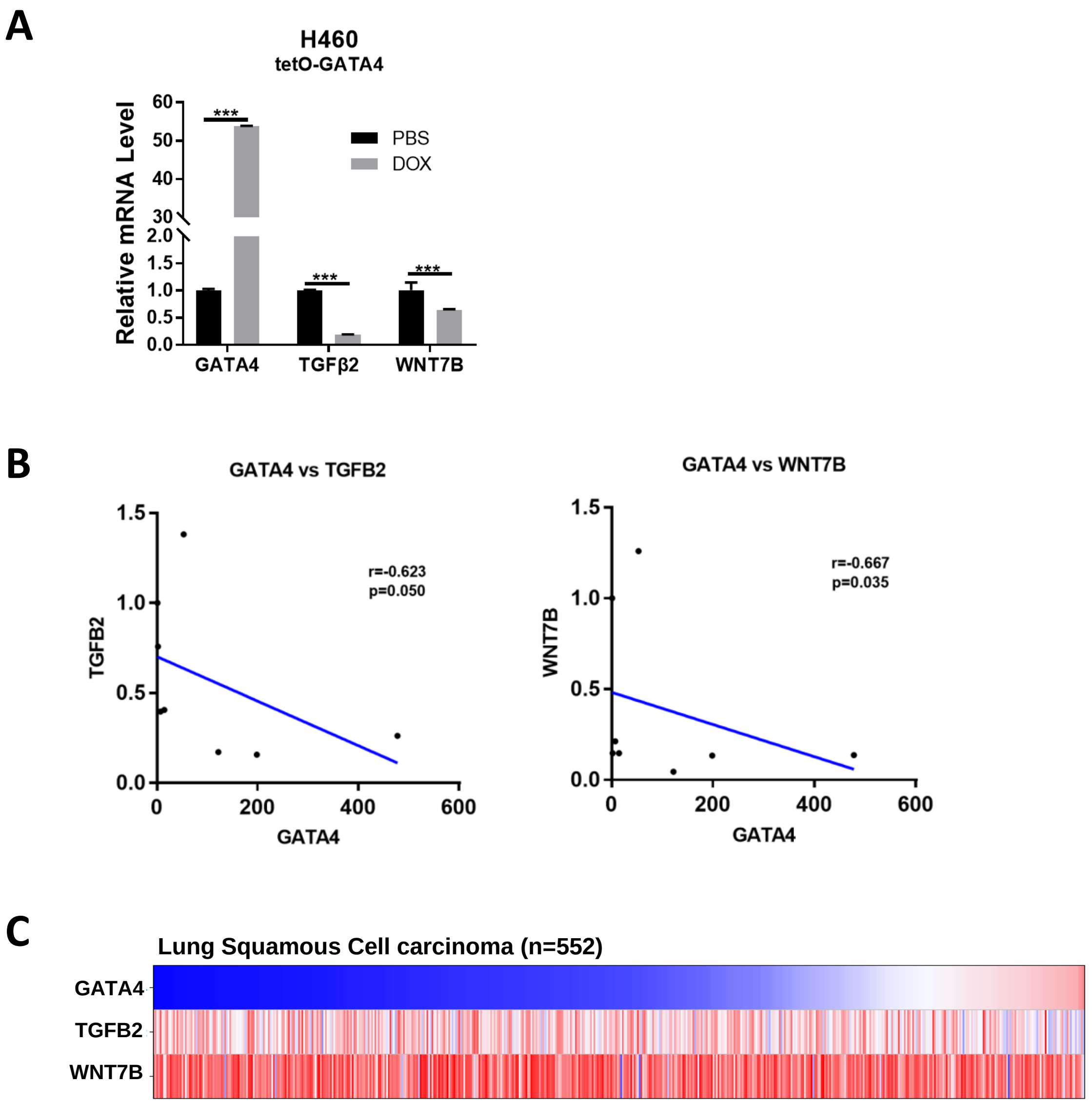

Supplementary Figure 6A. Stable H460 cell lines harboring TetO-GATA4 cells were treated with PBS or DOX for 48 hours. mRNA was extracted to quantify the expression level of GATA4, TGFβ2 and Wnt7b (n = 3 per group). **B.** GATA4 gene expression correlates negatively with TGFB2 and WNT7B in normal lung epithelial cell line and lung adenocarcinoma cell lines (n = 8). **C.** Expression pattern of GATA4 versus TGF-β2 and Wnt-7b in lung squamous carcinoma patients. Normalized expression data of GATA4, TGFB2 and WNT7B in lung squamous cell carcinoma patients were downloaded from https://confluence.broadinstitute.org/display/GDAC/Download (n = 552) and used log2 value to represent gene expression in each sample.

Supplementary Figure 7

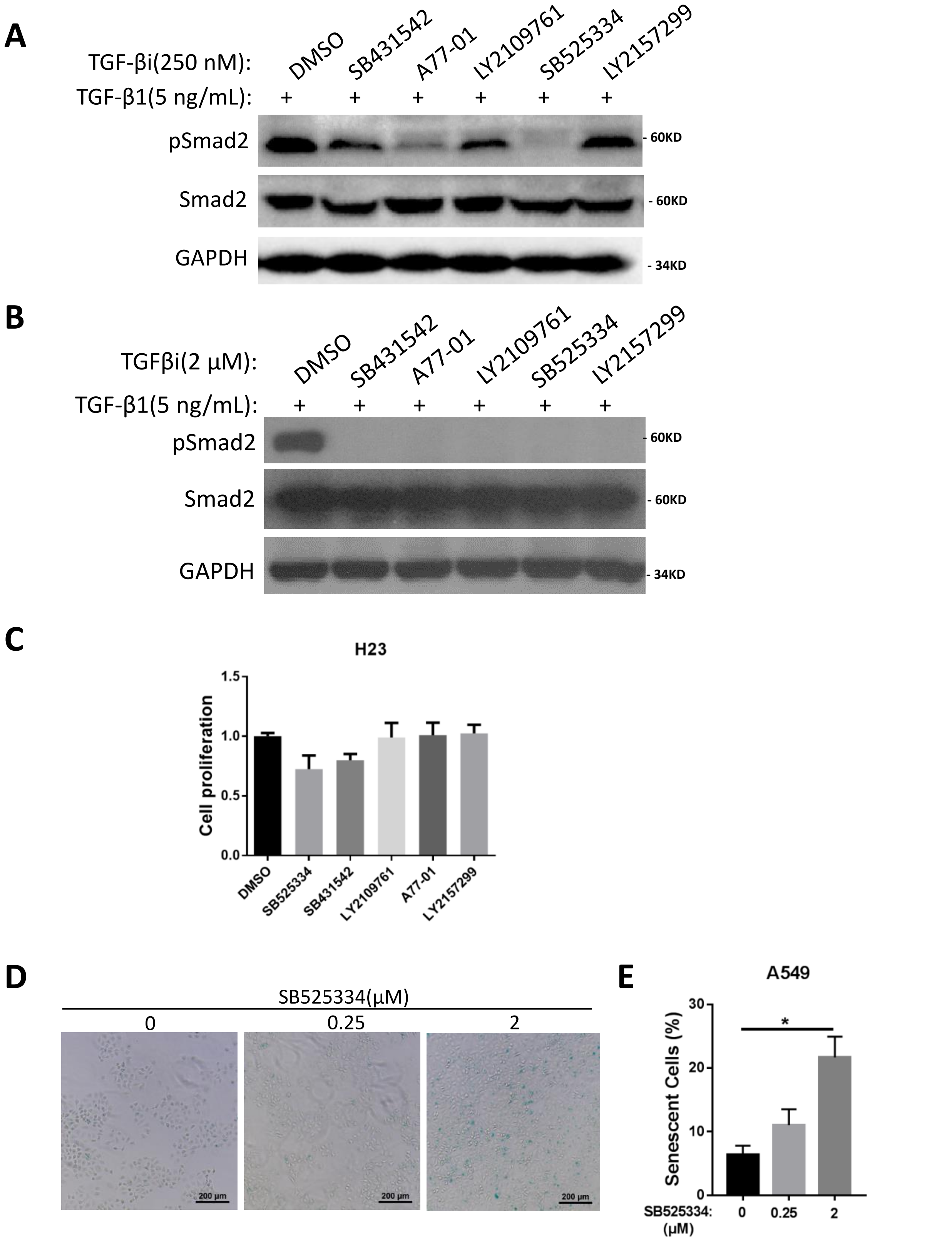

F

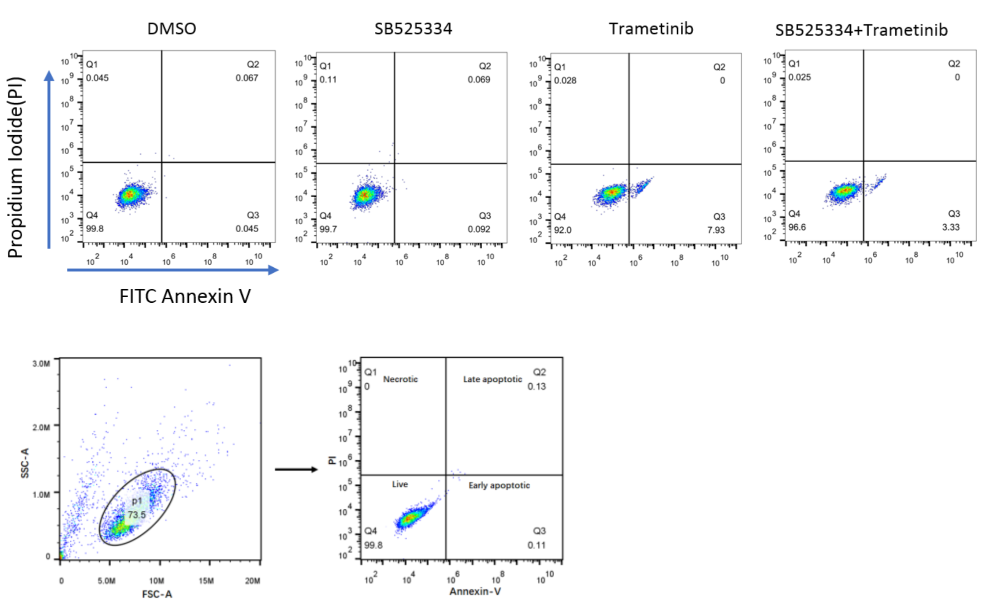
DMSO
SB525334
Trametinib
SB525334+Trametinib
Propidium Iodide(PI)
FITC Annexin V
Q1
Q2
Q3
Q4
0.045
0.067
99.8
0.045
0.11
0.069
99.7
0.092
0.028
0
92.0
7.93
0.025
0
96.6
3.33
SSC-A
FSC-A
P1
73.5
PI
Annexin-V
Necrotic
Late apoptotic
Live
Early apoptotic
0
0.13
99.8
0.11

G

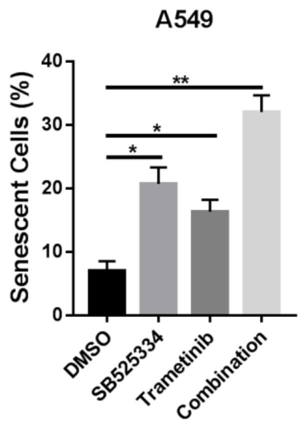
A549
Senescent Cells (%)
**
*
*
DMSO
SB525334
Trametinib
Combination

H

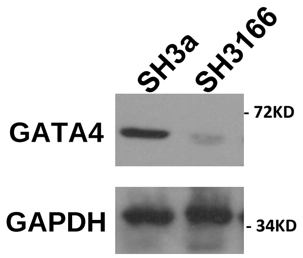
SH3a
SH3166
GATA4
GAPDH
- 72KD
- 34KD

I

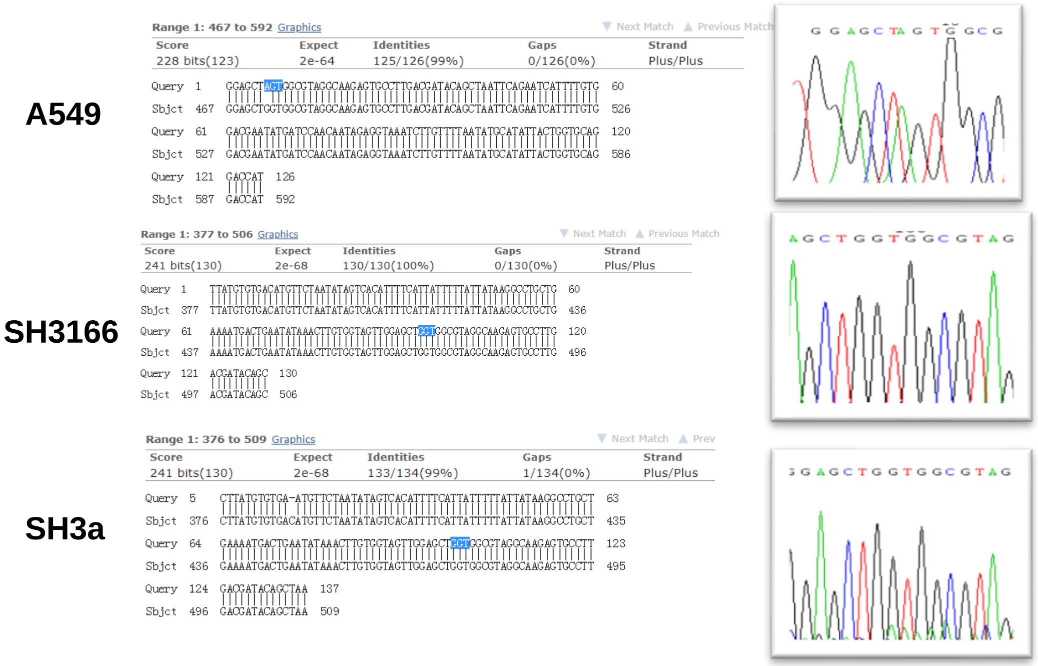
A549
Range 1: 467 to 592 Graphics
Score
Expect
Identities
Gaps
Strand
228 bits(123)
2e-64
125/126(99%)
0/126(0%)
Plus/Plus
SH3166
Range 1: 377 to 506 Graphics
241 bits(130)
2e-68
130/130(100%)
0/130(0%)
Plus/Plus
SH3a
Range 1: 376 to 509 Graphics
241 bits(130)
2e-68
133/134(99%)
1/134(0%)
Plus/Plus

J

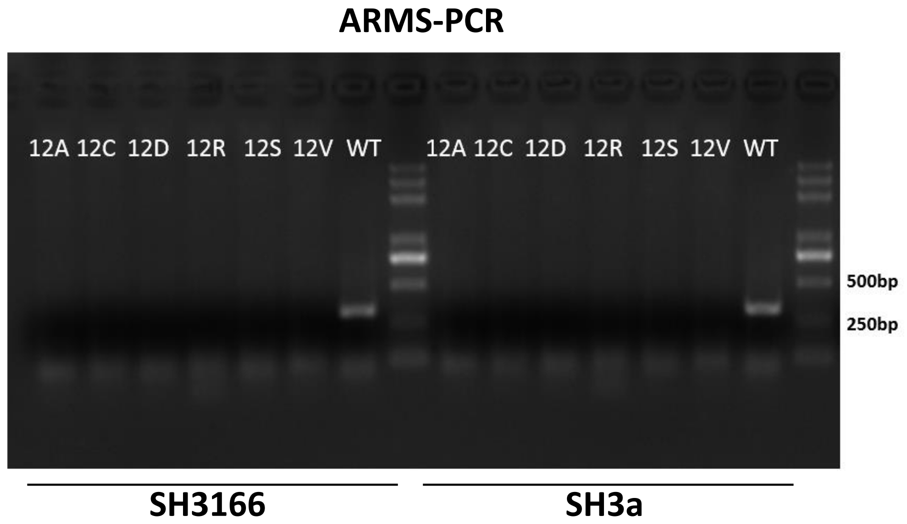
ARMS-PCR
12A
12C
12D
12R
12S
12V
WT
12A
12C
12D
12R
12S
12V
WT
500bp
250bp
SH3166
SH3a

K

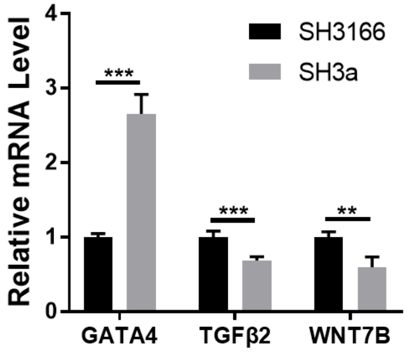
Relative mRNA Level
0
1
2
3
4
SH3166
SH3a
***
***
**
GATA4
TGFβ2
WNT7B

Supplementary Figure 7A&B. A549 cells were treated with TGF-β1(5ng per mL) and TGFBR inhibitors at indicated concentration for 2 hours. Protein was extracted for detecting phosphorylation of SMAD2. 250 nM in **A** and 2 μM in **B**. **C.** CCK8 assay determining growth rate of H23 cells treated with indicated inhibitors at 250 nM. **D** (n = 3 per group). A549 cells were treated with SB525334 for 2 days and β-galactosidase staining conducted to verify cell senescence. **E.** Statistics of **D** (n = 3 per group). **F.** A549 cells treated with SB525334 (2μM), Trametinib (2μM), and the combination for 48 hours. Annexin V and PI staining for apoptosis analysis. Results of FACS analysis were shown (upper panel). Gating strategy is shown in lower panel. **G.** A549 cells treated with SB525334 (2μM), Trametinib (2μM), and the combination for 48 hours. β-galactosidase staining conducted to verify cell senescence (n = 3 per group). **H.** Western analysis of expression level of GATA4 in SH3a and SH3166 PDX samples. **I.** Genomic DNA was extracted from A549, SH3166 and SH3a, and performed PCR with primers: KrasF: tggtcctgcaccagtaatat and KrasR: aaggtactggtggagtattt. The PCR products were subjected to Sanger sequencing**. J.** ARMS-PCR was performed to detect the mutation of SH3a and SH3166. ARMS-PCR was performed using primers as follows: G12A: ATATAAACTTGTGGTAGTTGGAGCTTC; G12C: AATATAAACTTGTGGTAG TTGGAGCCT; G12D: ATATAAACTTGTGGTAGTTGGAGCGGA; G12R: AAT ATAAACTTGTGGTAGTTGGAGCTC; G12S: AATATAAACTTGTGGTAGTTG GAGCGA: G12V: ATATAAACTTGTGGTAGTTGGAGCAGT; Reverse: ATGCAC AGAGAGTGAACATCATGGAC; WT: AATATAAACTTGTGGTAGTTGGAGCTG. **K.** mRNA extracted from SH3166 and SH3a. qPCR was performed to detect the expression level of GATA4, TGFb2 and Wnt7b (n = 3 per group). Bars are represented as mean ± SEM of the indicated

number (n) of repeats. *P < 0.05, **P < 0.01, and ***P < 0.001 by Students’ t-test.

Supplementary methods:

2.1 Cell Culture and Generation of Engineered Cell Lines

A549, NCI-H460, NCI-H226, NCI-H23, EKVX, Hop62, Beas-2b, PC-9, HEK293T and Phoenix cells were purchased from ATCC (American Typical Culture Collection, Manassas, VA, USA), while HBEC (human bronchial epithelial cells) and HSAEC (human small airway epithelial cells) cell lines were kindly gifted by Dr. John Minna from the University of Texas Southwestern Medical Center. Beas-2b, HEK293T and Phoenix cells were cultured in DMEM plus 10% fetal bovine serum (FBS, Gibco, Life Technologies, Carlsbad, CA, USA). HBEC and HSAEC cells were cultured in SAGM medium (Lonza, Allendale, NJ, USA), and the rest of the NSCLC cell lines were cultured in RPMI-1640 with 10% FBS. To generate the doxycycline (Dox)-inducible A549 (A549i) cell line, A549 cells were infected simultaneously with retrovirus encoding TetO-GATA4 (packaged from pREV-TRE-GATA4 vector) and retrovirus encoding EF1α-rtTA-iresGFP (packaged from pWPI-rtTA-iresGFP), then selected with 200 μg per mL Hygromycin for 2 weeks and FACS sorted for GFP positive cells. For transient GATA4 expression, cell lines were infected with virus packaged from pWPI-GATA4 or control virus packaged from control construct pWPI-EGFP. For dox-induced knockdown of TGFβ2 and Wnt7b, pLKO-tet-puro vector harboring shRNA sequence targeting TGFβ2 or Wnt7b was packaged and infected A549 cells, then selected with 1ug per mL puromycin for 1 week. The lung cancer cell lines were authenticated by China Center for Type Culture Collection. All cell lines were maintained in mycoplasma-free environment by adding MYCO-3 (A5240,0020, AppliChem GmbH) and verified through PCR analysis (F: 5’-GGGAGCAAACAGGATTAGATACCCT-3’

R: 5’-TGCACCATCTGTCACTCTGTTAACCTC-3’).

To establish TGFβ2 expressing A549 stable cell line, HA-tagged TGFβ2 expressing DNA cassette CAG- TGFβ2-IRES-Neo was also electroporated (140 V, 25 ms) into A549 cell and selected with 700 µg per mL G418 for 2 weeks. For shRNA knockdown, cells were infected with shRNA lentivirus (pLKO.1), selected with 2~3 µg per mL puromycin for 7~10 days. All cells were cultured in a 37°C humidified atmosphere containing 5% CO2.

2.2 Plasmids

pCMV6-Entry-GATA4 (Myc-FLAG tagged) was purchased from Origene (Rockville, MD, USA) and subcloned into lentivirial pWPI and retro-virial pREV-TRE vector respectively. The pWPI plasmid was a gift from Dr. Feng Shao at the National Institute of Biological Sciences (NIBS, Beijing, China). CAG-IRES-Neo plasmid (constructed by Shinya Yamanaka) was purchased from (Addgene plasmid#13461, Addgene, Cambridge, MA, USA) and the pREV-TRE retroviral plasmid and pTeton advanced plasmid were purchased from Clontech (Mountain View, CA, USA). Human TGFβ2 and CTNNB1 cDNA were purchased from Life Technologies and then subcloned into CAG-IRES-Neo vector respectively. The Top-Flash plasmid was a gift from Dr. Wei Wu’s lab at Tsinghua University. The pSIN-DEST51-d2EGFP was a gift from Dr. Yangming Wang at Peking University for construct microRNA sponge. All the following shRNAs were from the Biological Resource Center at NIBS, purchased from the Sigma Mission shRNA Library:

shGATA4#1(TRCN0000020424), shGATA4#2(TRCN0000329713),

shTGFB2(TRCN0000196326), shWnt7b(TRCN0000061877),

shTGFBR1(TRCN0000195626), shTGFBR2(TRCN0000040010),

shSMAD2(TRCN0000010477), shSMAD4(TRCN0000010323),

shBTBD11(TRCN0000136604), shDLX4(TRCN0000013776),

shEOMES (TRCN0000013175), shCDYL (TRCN0000276335)

shNkx2.5#1(TRCN0000013733), shNkx2.5#2(TRCN0000013734),

shSmarcd3#1(TRNC0000147806), shSmarcd3#2(TRCN0000179523),

shRelA#83 (TRCN0000014683), shRelA#86(TRCN0000014686),

shMMP7#1(TRCN0000304140), shMMP7#2(TRCN0000051843),

shCTNNB1#1(TRCN0000314991), shCTNNB1#2(TRCN0000314920),

shCDKN2B#1(TRCN0000038155), shCDKN2B#2(TRCN0000038156)

shCDKN2B#3(TRCN0000038157) and Luciferase shRNA (shLuc, SHC007) as a control shRNA.

2.3 siRNA screen assay

Dharmacon siRNA reagent (# G-005805-01) was delivered into H23 cell through Reverse transfection method (Erfle et al., 2007). Briefly, siRNA and lipid (Lipofectamine® 2000 Reagent) were mixed and added to 96-wells plates. Then, H23 cells were added to the plates (10,000 cells per well). Cell proliferation rate was measured on day 3 by CCK8 assay. During screening, siRNAs targeting EGFP (5'- CGTGATCTTCACCGACAAGAT-3') are included in the upper- and lower- most rows of each plate, which served as negative control. The screening was conducted once.

2.4 Cell Proliferation Assay and Cell Titer-Glo Assay

For cell proliferation assays, 50,000 A549i cells were seeded in each well of 12-well plates and cultured overnight. Dox was then added to a final concentration of 2 µg per mL. The cells were counted every other day.

For the Cell Titer-Glo assays, 5,000 cells were seeded into each well of 96-well plates and cultured overnight, and the cells were cultured for another 48h after adding Dox. Luminescence was measured using Cell Titer-Glo reagent (Promega, Madison, WI, USA) according to the manufacturer's instructions. Doxycycline hyclate (Sigma, St. Louis, MO, USA) was dissolved in ddH_2O (2 mg per mL) and stored at -80 °C.

2.5 Soft-agar Colony Formation Assay

Colony formation assays were performed in soft agar (0.6% lower gel and 0.35% upper gel) in 6-well plates. A549 (10,000 cells), PC-9 (10,000 cells), H460 (10,000 cells), Beas-2b (10,000 Cells), NCI-H226 (10,000 cells), or NCI-H23 (10,000 cells) cells were seeded into each well of 6-well plates. 2 µg per mL Dox was initially mixed with the upper gel, and 0.5 mL 1 x fluid media containing Dox was added to the surface of the upper gel every week. After 3~4 weeks, colonies were stained with 0.05% crystal violet. Colonies with the diameter over 200 µm were counted. The results are presented as mean values of triplicates.

2.6 Virus Packaging and Concentration

Lentiviral plasmids were co-transfected with helping plasmids, psPAX2 and PMD2.G (deposited by Didier Trono, Addgene plasmid #12260 and #12259) into HEK293T cells using transfection reagent VigoFect (Vigorous Biotechnology, Beijing, China). The cells were washed with fresh medium 6~8h post transfection and cultured in fresh media. After 48h of culture, virus containing supernatant was collected. To package retrovirus, viral plasmid and helping plasmid pCL-Eco (deposited by Inder Verma, Addgene plasmid#12371) were co-transfected into Phoenix cells, and virus containing medium was collected as described above. All the retroviruses used in the mouse experiments were condensed by the following procedure: virus containing medium was

centrifuged at 27,500rpm for 2h. The supernatant was carefully removed and the virus particles were resuspended with 100 μL Opti-medium. Shaking gently at 4°C overnight, the virus was prepared for use.

2.7 RNA extraction and Reverse-transcription PCR

Total RNA was extracted using Trizol reagent. 2 μg total RNA was reverse-transcribed to cDNA using the Takara M-MLV Reverse Transcriptase Kit (Takara, Dalian, China). Human GAPDH gene was used as an internal control. Real-Time PCR was done by using an ABI 7500 Fast Real-Time PCR machine (Applied Biosystems, Life Technologies) and SYBR Premix Ex Taq II reagent (Takara). The following primers were used in the experiments:

TGFβ2-Forward: 5’- CTGTCTACCTGCAGCACACT-3’,

TGFβ2-Reverse: 5’- TGGGACTGTCTGGAGCACAA -3’,

Wnt7b-Forward: 5’- CGCAGCTATCAGAAGCCCAT-3’,

Wnt7b-Reverse: 5’- CAGGTGTTGCACTTGACGA-3’

TGFBR1-Forward: 5’- CACAGAGTGGGAACAAAAAGGT-3’

TGFBR1-Reverse: 5’- CCAATGGAACATCGTCGAGCA-3’

TGFBR2-Forward: 5’- GTAGCTCTGATGAGTGCAATGAC-3’

TGFBR2-Reverse: 5’- CAGATATGGCAACTCCCAGTG-3’

SMAD4-Forward: 5’- ACGAACGAGTTGTATCACCTGG-3’

SMAD4-Reverse: 5’- TGCACGATTACTTGGTGGATG-3’

MMP7-Forward: 5’- ATGTGGAGTGCCAGATGTTGC-3’

MMP7-Reverse: 5’- AGCAGTTCCCCATACAACTTTC-3’

Gapdh-Forward:5’-GAAGGTGAAGGTCGGAGTC-3’

Gapdh-Reverse:5’-GAAGATGGTGATGGGATTTC-3’

2.8 Protein extraction and immunoblotting

Whole cell lysates were extracted by using the lysis buffer: 50 mM Tris pH7.4, 150 mM NaCl, 1 mM EDTA, 1% Triton, and 10% Glycerol along with protease and phosphatase inhibitor cocktail (Roche, Basel, Switzerland) protein concentrations were determined by the Bradford assay. Soluble proteins (30~40 μg) were subjected to SDS-polyacrylamide gel electrophoresis. Separated proteins were electrophoretically transferred onto polyvinylidene difluoride (PVDF) membranes (Millipore, Billerica, MA,USA) and immunoblotted with anti-GATA4 (Epitomics, Burlingame, CA, USA), -FLAG (Sigma), -CyclinD1 (Cell Signaling Technology, CST, Danvers, MA, USA), -KRAS (Proteintech, Chicago, IL, USA), -Phos-ERK1/2 (CST), -Phos-AKT1 (CST), -ERK1/2 (CST), -AKT1 (CST), -PTEN (CST), -c-MYC (Sigma), -p21(CST), -p27(CST), -p53(CST), -p14/ARF(CST), -p16/Ink4a (Epitomics), -p15 (ImmunoWay, Newark, DE, USA) or -β-actin (Sigma) antibody. Immunoreactive proteins were visualized using ECL Western Blotting Substrate（PREGENE, Beijing, China）and X-ray films.

2.9 Mouse studies

The animal study was performed following internationally recognized animal care and use guidelines and approved by the Jinan University, Guangdong, China. Mouse lines of CC10-rtTA, TRE-EGFR-T790M/Del19 (referred to as TRE-EGFR-TD) were kept in lab. The CC10-rtTA; TRE-KrasG12C mice were sacrificed after 1 month of doxycycline diet feeding, while the CC10-rtTA; TRE-EGFR-TD mice were sacrificed after 3 months of feeding when the mice showed obvious panting phenotype. For immunohistochemistry, mouse lung was inflated with 10% neutral buffered formalin solution (Sigma, St. Louis, MO, USA) and incubated overnight. Paraffin embedded lung tissue was cut into 5 μm slices for immunohistochemical

staining.

For the single-dose virus delivery experiment, all the littermates of double transgenic mice were randomly divided into 2 groups of 3-4 mice. GATA4 or mCherry retroviruses were delivered to mouse lung via nostril inhalation. After 1 week of recovery, all the experimental mice were fed Dox containing food until the day of sacrifice. Mouse lungs were collected for hematoxylin & eosin (H&E) staining. For quantification of tumor burden in the CC10-rtTA; TetO- KrasG12C mice, we calculated the total size (mm^2) of all the tumor regions in H&E sections under a microscope. For the CC10-rtTA; TetO-EGFR-TD mice, we counted the number of visible tumor nodules in the whole lung.

2.10 Senescence associated β-Galactosidase (SA-β-Gal) staining assay

SA-β-Gal staining was performed using the Cell Signaling Senescence β-Galactosidase Staining Kit (CST #9860). Briefly, 20,0000~30,0000 cells were seeded in each well of 6-well plates and cultured until the time of staining. For Dox treatment, a final concentration of 2 μg per mL was used and Dox containing medium was changed every other day. Wnt pathway inhibitors, ICG-001, XAV939, were purchased from Selleckchem (Houston, TX, USA) and were all dissolved in DMSO. Chemicals were added to the A549 cells (DMSO<0.1%) for 4 days treatment and then underwent SA-β-Gal staining to see their effect in senescence.

For quantification of SA-β-Gal positive cells elicited by GATA4 expression, blue positive cells in at least six randomly selected fields at 200× magnification under an inverted microscope were counted. For shRNA knockdown cells, we calculated the percentage of SA-β-Gal cells in the three picked observed fields. The β-Gal staining for each group was experimentally repeated three times.

For SA-β-Gal staining of lungs tissues, kras^{G12D};GATA4-/- mice were treated with inhibitors as indicated were fixed, wash with PBS/NP-40, and incubated in staining solution for β-galactosidase. Stained lungs were fixed 4% paraformaldehyde and embedded in paraffin. Section of 5 microns were stained with fast nuclear red. Senescence signaling was counted on cells stained blue.

2.11 Top-Flash Assay

500ng Top-Flash plasmid mixed with 200ng CAG-CTNNB1-IRES-Neo plasmid and 500ng pCMV6-GATA4 plasmid or 500ng Empty Vector was transfected to A549 cell seeded in 12-well plate via VigoFect reagent. Both the treatment had three replicates. After 48h-culture, A549 cells were trypsinized and counted. 100μL culture medium within 20,000 A549 cells was mixed with 100μL Firefly Luciferase reagent (Promega, Madison, WI, USA) to measure the value of luminescence. For evaluation the effect of Wnt pathway inhibitors in Top-Flash assay, three inhibitors were added to the A549 cells post 24h-transfection for another 48h incubation until the day of measurement.

2.12 microRNA Assay

Total RNA of A549 cell was extracted by Trizol as described previously. 1 μg total RNA was reverse transcribed microRNAs via one step polyA tailed assay in the following 25μL mixture: 25mM dNTP(2.5μL), 100mM ATP(2.5μL), 2.5mM $MnCl_2$ (2.5μL), 10×E.coil PolyA Polymerase buffer(2.5μL), QmiR-RT primer(5’-GCGAGCACAGAATTAATACGACTCACTATAGGTTTTTTTTTTTTTTTTTTVN-3’,500ng),

RNase Inhibitor(0.5μL), M-MLV Reverse Transcriptase (1μL) (Takara) and E.coil PolyA Polymerase (1μL) (NEB, Ipswich, MA, USA). The reaction was run at 37℃ for 90min and inactivated at 95℃ for 10min. The following microRNAs primers were used in the Real-Time PCR and human U6 was an internal control.

qmiR-Reverse: 5’-GCGAGCACAGAATTAATACGAC-3’

hsa-miR-590-Forward: 5’-CGGCGGTAATTTTATGTATAAGCTAG-3’

hsa-miR-320c-1-Forward: 5’-GCGAAAAGCTGGGTTGAGAGGG-3’

hsa-miR-320a-Forward: 5’-TCGGAAAAGCTGGGTTGAGAGGGC-3’

hsa-miR-32-Forward: 5’-CGGCG TATTGCACATTACTAAGT-3’

hsa-miR-301b-Forward: 5’-CGGCGGCAGTGCAATGATATTGTC-3’

hsa-miR-29c-Forward: 5’-CGCCGTAGCACCATTTGAAATCGG-3’

hsa-miR-29b-1-Forward: 5’-CGGCGGTAGCACCATTTGAAATC-3’

hsa-miR-29a-Forward: 5’-TCGGTAGCACCATCTGAAATCGG-3’

hsa-miR-21-Forward: 5’-GCCGCTAGCTTATCAGACTGATG-3’

hsa-miR-199b-Forward: 5’-TCGGCCCAGTGTTTAGACTATCTG-3’

hsa-miR-193b-Forward: 5’-CGGGGTTTTGAGGGCGAGATGA-3’

hsa-miR-103b-2-Forward: 5’-TCATAGCCCTGTACAATGCTGC-3’

U6-Forward: 5’-CTCGCTTCGGCAGCACA-3’

U6-Reverse: 5’-AACGCTTCACGAATTTGCGT-3’

Four microRNA precursors were subcloned into the pLenti6 lenti-vrial vector (a gift from Dr. Zhiqian Zhang’s lab at Beijing Cancer Hospital) and 10μg per mL blasticidin (Invitrogen) was used to select microRNA expressing A549 cells. The following pre-sequences of four microRNAs

were used in the experiment.

hsa-miR-590:

5'-TAGCCAGTCAGAAATGAGCTTATTCATAAAAGTGCAGTATGGTGAAGTCAATCTGT

AATTTTATGTATAAGCTAGTCTCTGATTGAAACATGCAGCA-3'

hsa-miR-320a:

5'-GCTTCGCTCCCCTCCGCCTTCTCTTCCCGGTTCTTCCCGGAGTCGGGAAAAGCTGG

GTTGAGAGGGCGAAAAAGGATAGGT-3'

hsa-miR-32:

5'-GGAGATATTGCACATTACTAAGTTGCATGTTGTCACGGCCTCAATGCAATTTAGTG

TGTGTGATATTTTC-3'

hsa-miR-301b:

5'-GCCGCAGGTGCTCTGACGAGGTTGCACTACTGTGCTCTGAGAAGCAGTGCAATG

ATATTGTCAAAGCATCTGGGACCA-3'

To block mature microRNAs, miRNA sponges targeting hsa-miR-590, hsa-miR-320a, hsa-miR-32 and hsa-miR-301b was inserted into pSIN-DEST51-d2EGFP. The reverse and forward DNA (detailed below) was annealed:

miRNA-Sponge-F:

TCGCCCTCTCGGTCAGCTTTTCCAGTGCAACTTAGCGCGTGCAATACCAGCTGCACTTT

TCCCATAAGCTCCCAGGCTTTGACAAGAGATTGCACTGCCAGATCG

miRNA-Sponge-R:

CTGGCAGTGCAATCTCTTGTCAAAGCCTGGGAGCTTATGGGAAAAGTGCAGCTGGTAT

TGCACGCGCTAAGTTGCACTGGAAAAGCTGACCGAGAGGGCGACGAT

2.13 Chromatin immunoprecipitation (ChIP)-Seq

ChIP was performed based on the protocol described previously (**Barski et al**.). Briefly, A549i cells were seeded in eight 150 mm plates, Dox was added to a final concentration of 2 µg per mL when the cells reached a confluence of 30%~40%. After 4 days of culture, the cells were crosslinked with 1% formaldehyde (final concentration) and sonicated using the following parameters: 5s on, 10s off, 15 cycles at the 25% set power (VCX500, SONICS, CT, USA). The total cell lysate was divided into two aliquots, one was mixed with 40 µL 1 mg per mL GATA4 antibody (6H10, Thermo Fisher Scientific, Waltham, MA, USA) and 40 µL Protein A/G Agarose beads (Pierce, Thermo Scientific), and the other was mixed with 40 µL 1 mg per mL normal mouse IgG (Sigma) and 40 µL Agarose beads. After overnight incubation, the beads were washed with ChIP washing buffer. Chromatin was eluted and the crosslink was reversed and DNA was extracted with Phenol/Chloroform. Finally, the eluted DNA was resolved in ddH_2O and processed to NIBS Sequencing Center for further high through-put seq. The result can be downloaded at http://www.ncbi.nlm.nih.gov/geo/query/acc.cgi?acc=GSE85003

2.14 Mouse treatment

GATA4 gRNAs were designed, validated and inserted in pSECC vector. Lentivirus virus was packaged from pSECC-sgGATA4 infected 293T cells, validated through cell infection and administered nasally into lsl-Kras^{G12D} mice. After tumor burden was confirmed by MRI imaging 12 weeks after virus infection, mice were treated with vehicle solution, SB525334 (Selleckchem) 10 mg per kg per day, Trametinib (a gifted from Qingsong Liu at High Magnetic Field Laboratory,

Chinese Academy of Sciences) 10 mg per kg per day and the combination of two drug. Lung tumor was documented again 2 weeks after drug dosing. The ratio of tumor size to chest size was calculated to quantify tumor burden. The tumor burden is calculated by area of tumors per area of lung.

For PDX assay, SH3166 or SH3a PDX was embedded to nude mice. Mice were treated with TGFBR inhibitor (SB525334, 10 mg per kg per day), Cisplatin (5 mg per kg, once a week) or combination as the size of xenograft between 100~200 mm^3. Two weeks after treatment, mice sacrificed. The tumor volume is calculated by the formula: Volume = 0.5 * $(width)^2$* length.

2.15 RNA-Sequencing

We used 4 tumor/paratumoral tissue pairs of lung cancer patients: a 57-year-old female, a 60 year-old female, a 51-year-old male, and a 47-year-old male adenocarcinoma patients. Tumor and adjacent tissues were ground into powder in liquid nitrogen; total RNA was harvested using RNeasy Mini Kit (QIAGEN); then total RNA samples were treated with DNase I and the mRNA is enriched by using the oligo(dT) magnetic beads.

RNA libraries were prepared for sequencing using standard Illumina protocols. Illumina Casava1.8 software used for base calling. Sequenced clean reads were mapped to Homo sapiens (hg19) whole genome using tophat v1.4.1 with parameters -i 10 -I 80000 --solexa1.3-quals --min-coverage-intron 20 --max-coverage-intron 11000 --min-segment-intron 10 --max-segment-intron 81000 --segment-length 20.

Gene annotation and calculation of FPKM values was carried out using Cufflinks (v2.0.2) with the provision of a GTF annotation file (hg19). Gene expression differences were assessed by Cuffdiff

use upper-quartile normalization, with false discovery rate correction for multiple testing. The sequencing result can be downloaded at https://www.ncbi.nlm.nih.gov/geo/query/acc.cgi?acc=GSE84852.

2.16 ARMS-PCR

Genomic DNAs were extracted from SH3166 and SH3a. ARMS-PCR was conducted on DNA samples with the primers listed below: **G12A:** ATA TAA ACT TGT GGT AGT TGG AGC TTC; **G12C:** AAT ATA AAC TTG TGG TAG TTG GAG CCT; **G12D:** ATA TAA ACT TGT GGT AGT TGG AGC GGA; **G12R:** AAT ATA AAC TTG TGG TAG TTG GAG CTC; **G12S:** AAT ATA AAC TTG TGG TAG TTG GAG CG A: **G12V:** ATA TAA ACT TGT GGT AGT TGG AGC AGT; **Reverse:** ATG CAC AGA GAG TGA ACA TCA TGG AC; **WT:** AAT ATA AAC TTG TGG TAG TTG GAG CTG.
