# Supplementary material for: Lung cancer deficient in the tumor suppressor GATA4 is sensitive to TGFBR1 inhibition
Source: Nat Commun. 2019 Apr 10;10:1665. doi: 10.1038/s41467-019-09295-7 (PMC6458308; doi:10.1038/s41467-019-09295-7)
Supplement: Supplementary file 2 — Description of Additional Supplementary Files [file 41467_2019_9295_MOESM2_ESM.docx]

**Description of Additional Supplementary Files**

File Name: Supplementary Data 1

Description: **siRNA screen of transcription factors in H23 for lung cancer tumor suppressor genes.** 5,000 H23 cells seeded into individual wells with siRNA sets targeting a specific transcription factor. CCK8 value of a well 3 days post siRNA transfection was normalized by that of a well with siRNA targeting EGFP.

File Name: Supplementary Data 2

Description: **RNA-seq of cancer versus normal lung tissues.** Tumor/para-tumoral tissue pairs from 4 lung cancer patients (a 57 year-old female (A126), a 60 year-old female (A236), a 51 year-old male (A401), and a 47 year-old male (A448) with adenocarcinoma) were used for RNA sequencing to get relative gene expression profile. Tumor and adjacent tissues were ground into powder in liquid nitrogen; total RNA was harvested using RNeasy Plant Mini Kit (QIAGEN) and treated with DNase I. mRNA is enriched by using the oligo(dT) magnetic beads. RNA libraries were prepared for sequencing using standard Illumina protocols. Log2 value of ratio of value of reads per kilo base (RPKB) of a gene in tumor sample against that in para-tumoral tissue (fold_change) is shown for 4 individual patients in 4 worksheets. The average of the log2 value is then used as relative expression value for transcription factors.

File Name: Supplementary Data 3

Description: **List of genes down-regulated in lung cancer and whose knockdown promoted H23 cell growth.**

File Name: Supplementary Data 4

Description: **GATA4 ChIP-Seq result.** A549i cells were treated with 2 µg/mL of Dox for 4 days. Cells were crosslinked with 1% formaldehyde (final concentration) and sonicated. The total cell lysate was treated with GATA4 antibody and Protein A/G Agarose beads. In the control group, total cell lysate was treated with mouse IgG and Agarose beads. After overnight incubation, the beads were washed. Chromatin was eluted and the crosslink was reversed. DNA was extracted with Phenol/Chloroform and dissolved in ddH2O and sequenced.

File Name: Supplementary Data 5

Description: **Number of DNA sequences bound by GATA4 as detected in ChIP-seq.** Number of DNA sequence bound by GATA4 calculated from Supplementary Table 4.

File Name: Supplementary Data 6

Description: **KEGG analysis of genes whose promoter were bond by GATA4.**

File Name: Supplementary Data 7

Description: **Supplementary Data 7. genes upregulated by ectopic expression of GATA4 in A549 cells.** RNA sequencing of Dox treated A549i and control treated A549i cells were used to derive the relative expression value for a particular gene. A ratio of above 2 (Dox treated against control treated) is regarded as upregulated.

File Name: Supplementary Data 8

Description: **Genes downregulated by ectopic expression of GATA4 in A549 cells.** RNA sequencing of Dox treated A549i and control treated A549i cells were used to derive the relative expression value for a particular gene. A ratio of below 0.5 (Dox treated against control treated) is regarded as downregulated.

File Name: Supplementary Data 9

Description: **KEGG analysis of upregulated genes.**

File Name: Supplementary Data 10

Description: **KEGG analysis of down-regulated genes.**

File Name: Supplementary Data 11

Description: **GO analysis of up-regulated genes.**

File Name: Supplementary Data 12

Description: **GO analysis of down-regulated genes.**

File Name: Supplementary Data 13

Description: **Detailed information of the seed regions of 14 miRNAs targeting TGFB2 mRNA.**
