# Supplementary material for: Lung cancer deficient in the tumor suppressor GATA4 is sensitive to TGFBR1 inhibition
Source: Nat Commun. 2019 Apr 10;10:1665. doi: 10.1038/s41467-019-09295-7 (PMC6458308; doi:10.1038/s41467-019-09295-7)
Supplement: Supplementary file 16 — Reporting Summary [file 41467_2019_9295_MOESM16_ESM.pdf]

nature research

Corresponding author(s): Liang Chen

Last updated by author(s): Jan, 11, 2019

Reporting Summary

Nature Research wishes to improve the reproducibility of the work that we publish. This form provides structure for consistency and transparency in reporting. For further information on Nature Research policies, see Authors & Referees and the Editorial Policy Checklist.

Statistics

For all statistical analyses, confirm that the following items are present in the figure legend, table legend, main text, or Methods section.

n/a	Confirmed	
☐	☒	The exact sample size (*n*) for each experimental group/condition, given as a discrete number and unit of measurement
☐	☒	A statement on whether measurements were taken from distinct samples or whether the same sample was measured repeatedly
☐	☒	The statistical test(s) used AND whether they are one- or two-sided<br>*Only common tests should be described solely by name; describe more complex techniques in the Methods section.*
☐	☒	A description of all covariates tested
☒	☐	A description of any assumptions or corrections, such as tests of normality and adjustment for multiple comparisons
☐	☒	A full description of the statistical parameters including central tendency (e.g. means) or other basic estimates (e.g. regression coefficient) AND variation (e.g. standard deviation) or associated estimates of uncertainty (e.g. confidence intervals)
☐	☒	For null hypothesis testing, the test statistic (e.g. *F*, *t*, *r*) with confidence intervals, effect sizes, degrees of freedom and *P* value noted<br>*Give P values as exact values whenever suitable.*
☐	☒	For Bayesian analysis, information on the choice of priors and Markov chain Monte Carlo settings
☒	☐	For hierarchical and complex designs, identification of the appropriate level for tests and full reporting of outcomes
☐	☒	Estimates of effect sizes (e.g. Cohen's *d*, Pearson's *r*), indicating how they were calculated

Our web collection on statistics for biologists contains articles on many of the points above.

Software and code

Policy information about availability of computer code

Data collection	For RNA-sequencing, Illumina Casava1.8 software used for base calling. Sequenced clean reads were mapped to Homo sapiens (hg19) whole genome using tophat v1.4.1 with parameters -i 10 -I 80000 --solexa1.3-quals --min-coverage-intron 20 --max-coverage-intron 11000 --min-segment-intron 10 --max-segment-intron 81000 --segment-length 20.
Data analysis	Gene annotation and calculation of FPKM values was carried out using Cufflinks (v2.0.2) with the provision of a GTF annotation file (hg19). Gene expression differences were assessed by Cuffdiff use upper-quartile normalization, with false discovery rate correction for multiple testing.

For manuscripts utilizing custom algorithms or software that are central to the research but not yet described in published literature, software must be made available to editors/reviewers. We strongly encourage code deposition in a community repository (e.g. GitHub). See the Nature Research guidelines for submitting code & software for further information.

Data

Policy information about availability of data

All manuscripts must include a data availability statement. This statement should provide the following information, where applicable:

- Accession codes, unique identifiers, or web links for publicly available datasets
- A list of figures that have associated raw data
- A description of any restrictions on data availability

RNA-sequencing data can be downloaded at https://www.ncbi.nlm.nih.gov/geo/query/acc.cgi?acc=GSE84852.

Field-specific reporting

Please select the one below that is the best fit for your research. If you are not sure, read the appropriate sections before making your selection.

☒ Life sciences ☐ Behavioural & social sciences ☐ Ecological, evolutionary & environmental sciences

For a reference copy of the document with all sections, see nature.com/documents/nr-reporting-summary-flat.pdf

Life sciences study design

All studies must disclose on these points even when the disclosure is negative.

Sample size	sample size was designed on the basis that statistical significance can be reached.
Data exclusions	no data were excluded during collection of our results
Replication	each experiment was repeated 3 times. Figures are shown as representative of 3 repeats.
Randomization	For mouse treatment study, the mice are MRI-imaged to confirm tumor tumor burden of lung cancer. Tumor-bearing mice are then randomized for treatment study.
Blinding	For pathological analysis of lung tumor and analysis of lung cancer shrinkage based on CT image, analyzer were blinded to to treatment information. The scored report are made into figures and the corresponding treatment information was filled in by researcher who did the treatment.

Behavioural & social sciences study design

All studies must disclose on these points even when the disclosure is negative.

Study description	not applicable
Research sample	not applicable
Sampling strategy	not applicable
Data collection	not applicable
Timing	not applicable
Data exclusions	not applicable
Non-participation	not applicable
Randomization	not applicable

Ecological, evolutionary & environmental sciences study design

All studies must disclose on these points even when the disclosure is negative.

Study description	not applicable
Research sample	not applicable
Sampling strategy	not applicable
Data collection	not applicable
Timing and spatial scale	not applicable
Data exclusions	not applicable
Reproducibility	not applicable

Randomization	not applicable
Blinding	not applicable

Did the study involve field work? ☐ Yes ☒ No

Field work, collection and transport

Field conditions	not applicable
Location	not applicable
Access and import/export	not applicable
Disturbance	not applicable

Reporting for specific materials, systems and methods

We require information from authors about some types of materials, experimental systems and methods used in many studies. Here, indicate whether each material, system or method listed is relevant to your study. If you are not sure if a list item applies to your research, read the appropriate section before selecting a response.

Materials & experimental systems

n/a	Involved in the study	
☐	☒	Antibodies
☐	☒	Eukaryotic cell lines
☒	☐	Palaeontology
☐	☒	Animals and other organisms
☒	☐	Human research participants
☒	☐	Clinical data

Methods

n/a	Involved in the study	
☐	☒	ChIP-seq
☐	☒	Flow cytometry
☒	☐	MRI-based neuroimaging

Antibodies

Antibodies used	All antibodies used are commercially available. We have provided catalog numbers and provider. Dilutions were indicated in manuscript.
Validation	antibodies are verified by blotting of 293T cell line transfected with vector control or vector for overexpressing the appropriate cDNA encoding the corresponding protein.

Eukaryotic cell lines

Policy information about cell lines

Cell line source(s)	A549, NCI-H460, NCI-H226, NCI-H23, EKVX, Hop62 were purchased from ATCC as part of NCI-60 (American Typical Culture Collection, Manassas, VA, USA). Beas-2b, PC-9, HEK293T and Phoenix cells were kindly provided by Dr. Kwok-Kin Wong (The Helen and Martin Kimmel Center for Stem Cell Biology, NYU). HBEC (human bronchial epithelial cells) and HSAEC (human small airway epithelial cells) cell lines were kindly gifted by Dr. John Minna from the University of Texas Southwestern Medical Center.
Authentication	The lung cancer cell lines were authenticated by China Center for Type Culture Collection.
Mycoplasma contamination	All cell lines were maintained in mycoplasma-free environment by adding MYCO-3 (A5240,0020, AppliChem GmbH) and verified through PCR analysis (F: 5’-GGGAGCAAACAGGATTAGATACCCT-3’ R: 5’-TGCACCATCTGTCACTCTGTTAACCTC-3’).
Commonly misidentified lines (See ICLAC register)	none of the cells used in our work was on the Version 9 Release Notes.

Palaeontology

Specimen provenance	not applicable
Specimen deposition	not applicable

Dating methods | not applicable

☐ Tick this box to confirm that the raw and calibrated dates are available in the paper or in Supplementary Information.

Animals and other organisms

Policy information about studies involving animals; ARRIVE guidelines recommended for reporting animal research

Laboratory animals	lsl-Kras G12D transgenic mice were on C57BL/6 background. male and female of 6-8 week old mice were used for inducing autochthonous lung cancers. Nude mice were purchased at 6-8 weeks for inoculating cell lines or patient-derived-tumors.
Wild animals	not applicable
Field-collected samples	not applicable
Ethics oversight	All experimental protocols were approved by the Institutional Committee for Animal Care and Use at Jinan University respectively and Shanghai Institutes for Biological Sciences respectively.

Note that full information on the approval of the study protocol must also be provided in the manuscript.

Human research participants

Policy information about studies involving human research participants

Population characteristics	Chinese male and female
Recruitment	Written informed consent was obtained from patients who are going to have their lung cancers surgically removed.
Ethics oversight	The protocol for human research was approved by Fudan University Shanghai Cancer Center.

Note that full information on the approval of the study protocol must also be provided in the manuscript.

Clinical data

Policy information about clinical studies

All manuscripts should comply with the ICMJE guidelines for publication of clinical research and a completed CONSORT checklist must be included with all submissions.

Clinical trial registration	not applicable
Study protocol	not applicable
Data collection	not applicable
Outcomes	not applicable

ChIP-seq

Data deposition

☒ Confirm that both raw and final processed data have been deposited in a public database such as GEO.

☒ Confirm that you have deposited or provided access to graph files (e.g. BED files) for the called peaks.

Data access links<br>*May remain private before publication.*	https://www.ncbi.nlm.nih.gov/geo/query/acc.cgi?acc=GSE84852.
Files in database submission	not applicable
Genome browser session<br>(e.g. UCSC)	not applicable

Methodology

Replicates	each experiment is repeated 3 times. a representative experiment was shown for each figure.
Sequencing depth	total reads 38752613, uniquely mapped reads 36635701, uniquely mapped ratio 94.54%, reads length 51bp, single-end.
Antibodies	GATA4 antibody (6H10, Thermo Fisher Scientific, Waltham, MA, USA)
Peak calling parameters	There are 6542 peaks with p-adj <0.01 and fold enrichment >5. The density of reads in each region was normalized to the

Peak calling parameters	total number of 10 million mapped reads. BigWig files were generated for visualization using Homer package.
Data quality	There are 6542 peaks with p-adj <0.01 and fold enrichment >5.
Software	ChIP-seq reads were aligned using Bowtie (version 1.1.2) to build version Hg19 of the Homo sapiens genome. MACS2 (version 2.0.10) was used to identify regions of ChIP-seq enrichment.

Flow Cytometry

Plots

Confirm that:

☒ The axis labels state the marker and fluorochrome used (e.g. CD4-FITC).

☒ The axis scales are clearly visible. Include numbers along axes only for bottom left plot of group (a 'group' is an analysis of identical markers).

☒ All plots are contour plots with outliers or pseudocolor plots.

☒ A numerical value for number of cells or percentage (with statistics) is provided.

Methodology

Sample preparation	Wash A549 cell line twice with cold PBS and then resuspend cells with PBS at a concentration of 10^6/ml. Transfer 100 µL cells to 1.5 ml tube. Add staining solution and incubate for 30min in dark. Analyze by flow cytometry.
Instrument	BD FACS Aria II
Software	FlowJo_X.0.7
Cell population abundance	10^6 cells per ml
Gating strategy	A549 cell without staining as negative control.

☒ Tick this box to confirm that a figure exemplifying the gating strategy is provided in the Supplementary Information.

Magnetic resonance imaging

Experimental design

Design type	Imaging the lung cancer in transgenic mouse models for treatment response evaluation
Design specifications	mice were MRI imaged to confirm lung cancer. Mice were then randomized into 4 groups (4 mice per group) for treatment with vehicle, MEK1/2 inhibitor, SB525334, and combination of MEK1/2 inhibitor and SB525334. Tumor burden were evaluated after 2 weeks' treatment.
Behavioral performance measures	not applicable

Acquisition

Imaging type(s)	structural
Field strength	1 Tesla
Sequence & imaging parameters	Sequence type: T1-weighted spin echo; Imaging type: EPI;<br>Coronal:<br>TR = 1000 ms; TE = 15 ms; Hor.Fov = 30 mm; Vert.Fov = 90 mm; Matrix = 171X512<br>Thickness = 0.8 mm; Inter-slice Gap = 0.1 mm; Flip angle = 90°<br>Axial:<br>TR = 1000 ms; TE = 15 ms; Hor.FOV = 30 mm; Ver.FOV = 30 mm; Matrix = 256X256<br>Thickness = 0.8; Inter-slice Gap = 0.1 mm; Flip angle = 90°
Area of acquisition	the whole mouse chest and abdomen

Diffusion MRI ☐ Used ☒ Not used

Preprocessing

Preprocessing software	Aspectimaging system software and VivoQuant
Normalization	Not needed. We only measured the lung tumor size in each object, and all acquisitions used the same sequence, the

Normalization	imaging parameters, and followed the same protocol. Only structural information was obtained for analysis, so there was no need for normalization in preprocessing.
Normalization template	N/A
Noise and artifact removal	N/A
Volume censoring	We used VivoQuant to segment lung cancers and measure their sizes

Statistical modeling & inference

Model type and settings	student T-test
Effect(s) tested	Evaluate the anti-tumor treatment response by measuring the lung cancer size variation in days in different groups

Specify type of analysis: ☐ Whole brain ☒ ROI-based ☐ Both

Statistic type for inference (See Eklund et al. 2016)	N/A
Correction	N/A

Models & analysis

n/a	Involved in the study
☒	☐ Functional and/or effective connectivity
☒	☐ Graph analysis
☒	☐ Multivariate modeling or predictive analysis

Functional and/or effective connectivity	*Report the measures of dependence used and the model details (e.g. Pearson correlation, partial correlation, mutual information).*
Graph analysis	*Report the dependent variable and connectivity measure, specifying weighted graph or binarized graph, subject- or group-level, and the global and/or node summaries used (e.g. clustering coefficient, efficiency, etc.).*
Multivariate modeling and predictive analysis	*Specify independent variables, features extraction and dimension reduction, model, training and evaluation metrics.*
