# Supplementary material for: Lung cancer deficient in the tumor suppressor GATA4 is sensitive to TGFBR1 inhibition
Source: Nat Commun. 2019 Apr 10;10:1665. doi: 10.1038/s41467-019-09295-7 (PMC6458308; doi:10.1038/s41467-019-09295-7)
Supplement: Supplementary file 17 — Source Data [file 41467_2019_9295_MOESM17_ESM.zip › Source Data.pptx]

## Slide 1
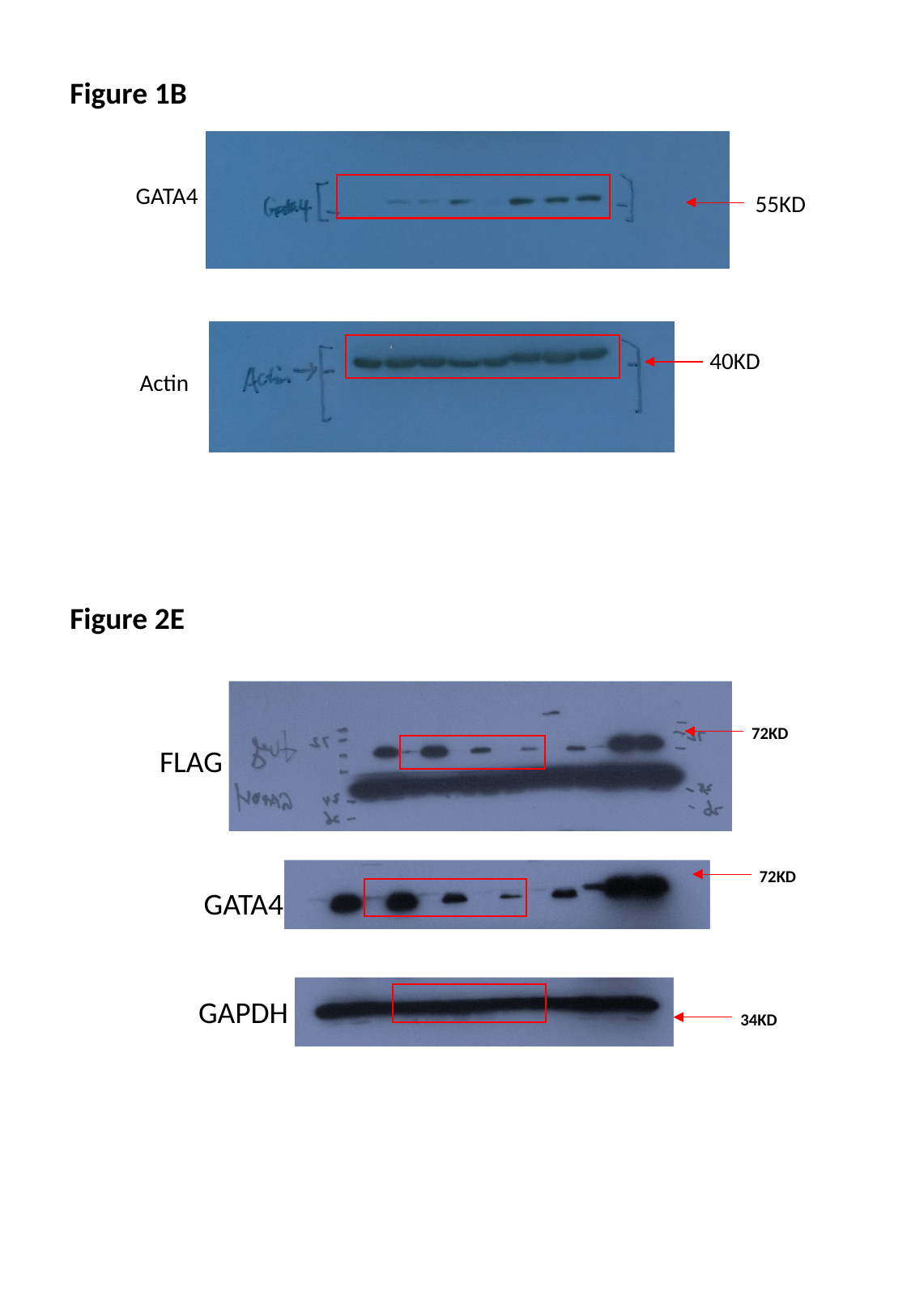

Figure 1B
GATA4
55KD
40KD
Actin
Figure 2E
72KD
FLAG
72KD
GATA4
GAPDH
34KD

## Slide 2
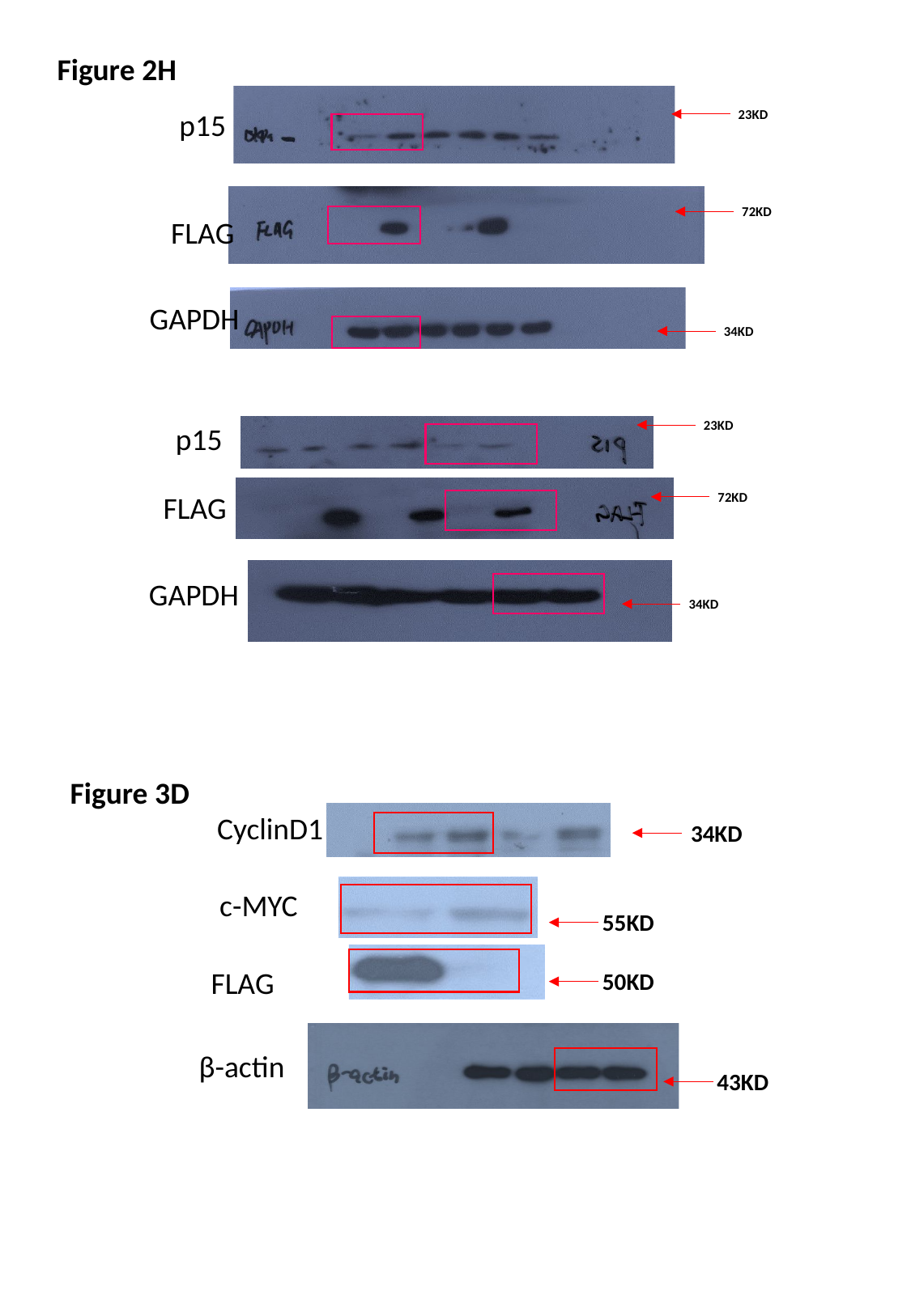

Figure 2H
23KD
p15
72KD
FLAG
GAPDH
34KD
23KD
p15
72KD
FLAG
GAPDH
34KD
Figure 3D
CyclinD1
34KD
c-MYC
55KD
FLAG
50KD
β-actin
43KD

## Slide 3
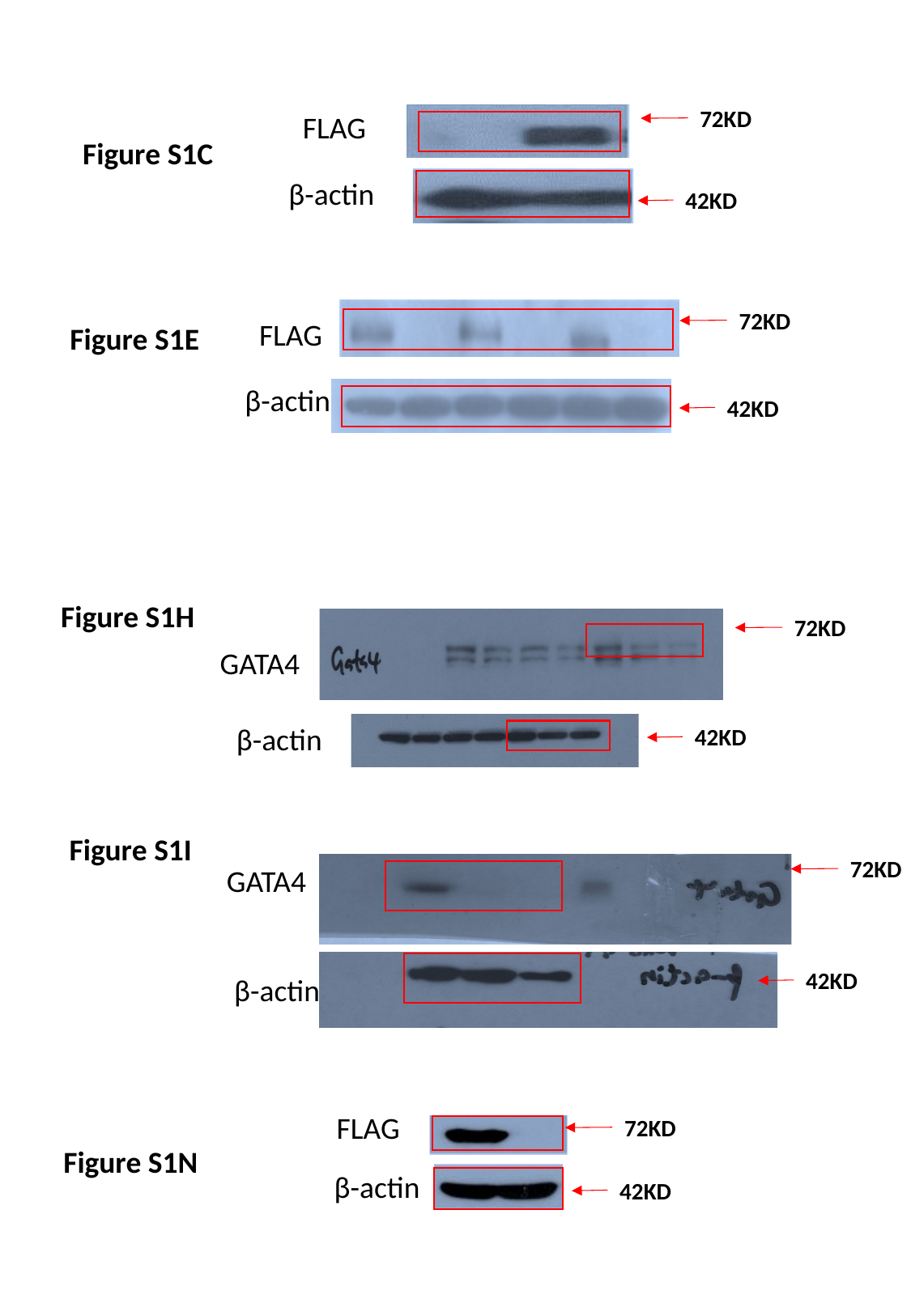

72KD
FLAG
Figure S1C
β-actin
42KD
72KD
FLAG
Figure S1E
β-actin
42KD
Figure S1H
72KD
GATA4
β-actin
42KD
Figure S1I
72KD
GATA4
42KD
β-actin
FLAG
72KD
Figure S1N
β-actin
42KD

## Slide 4
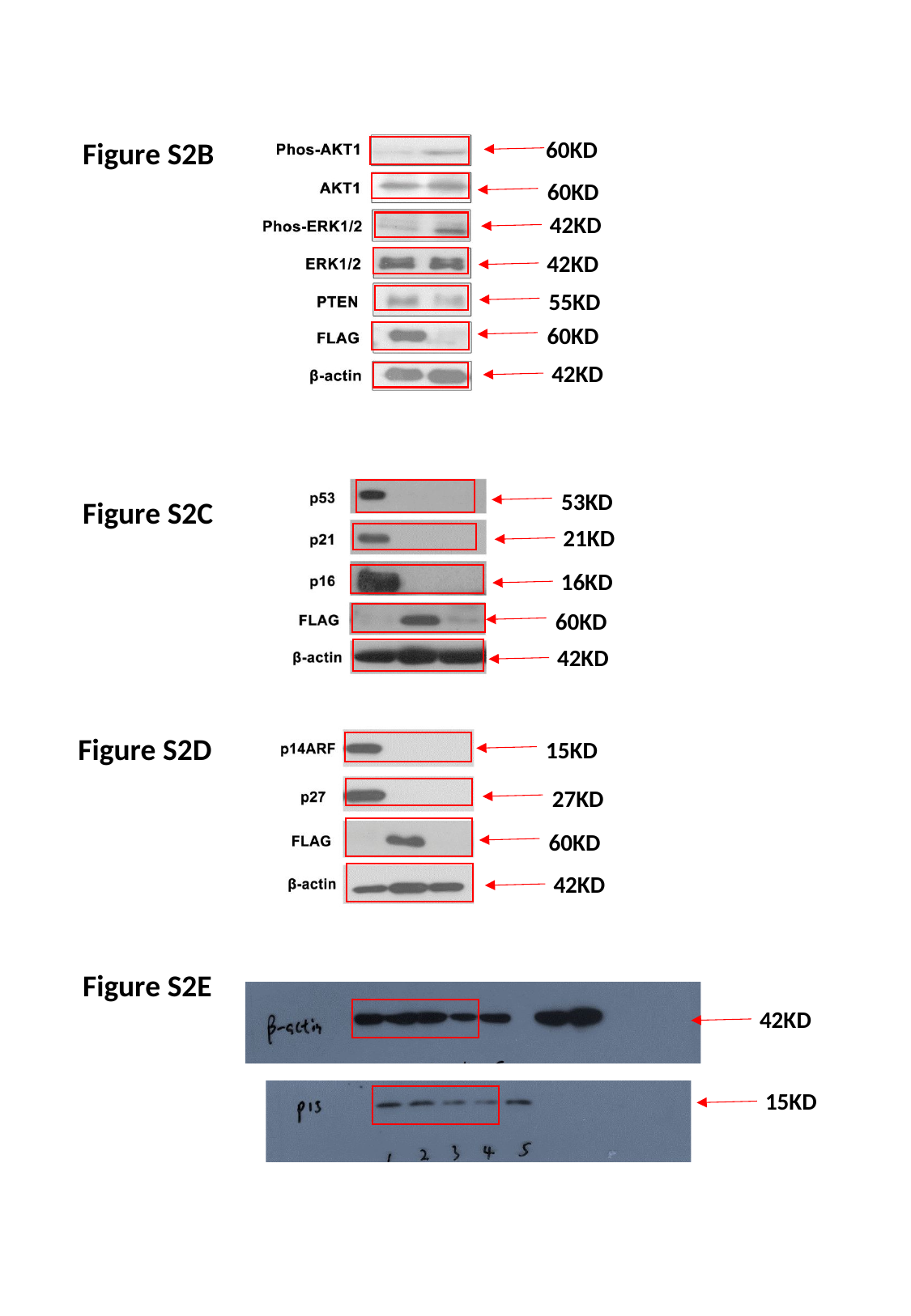

Figure S2B
60KD
60KD
42KD
42KD
55KD
60KD
42KD
53KD
Figure S2C
21KD
16KD
60KD
42KD
Figure S2D
15KD
27KD
60KD
42KD
Figure S2E
42KD
15KD

## Slide 5
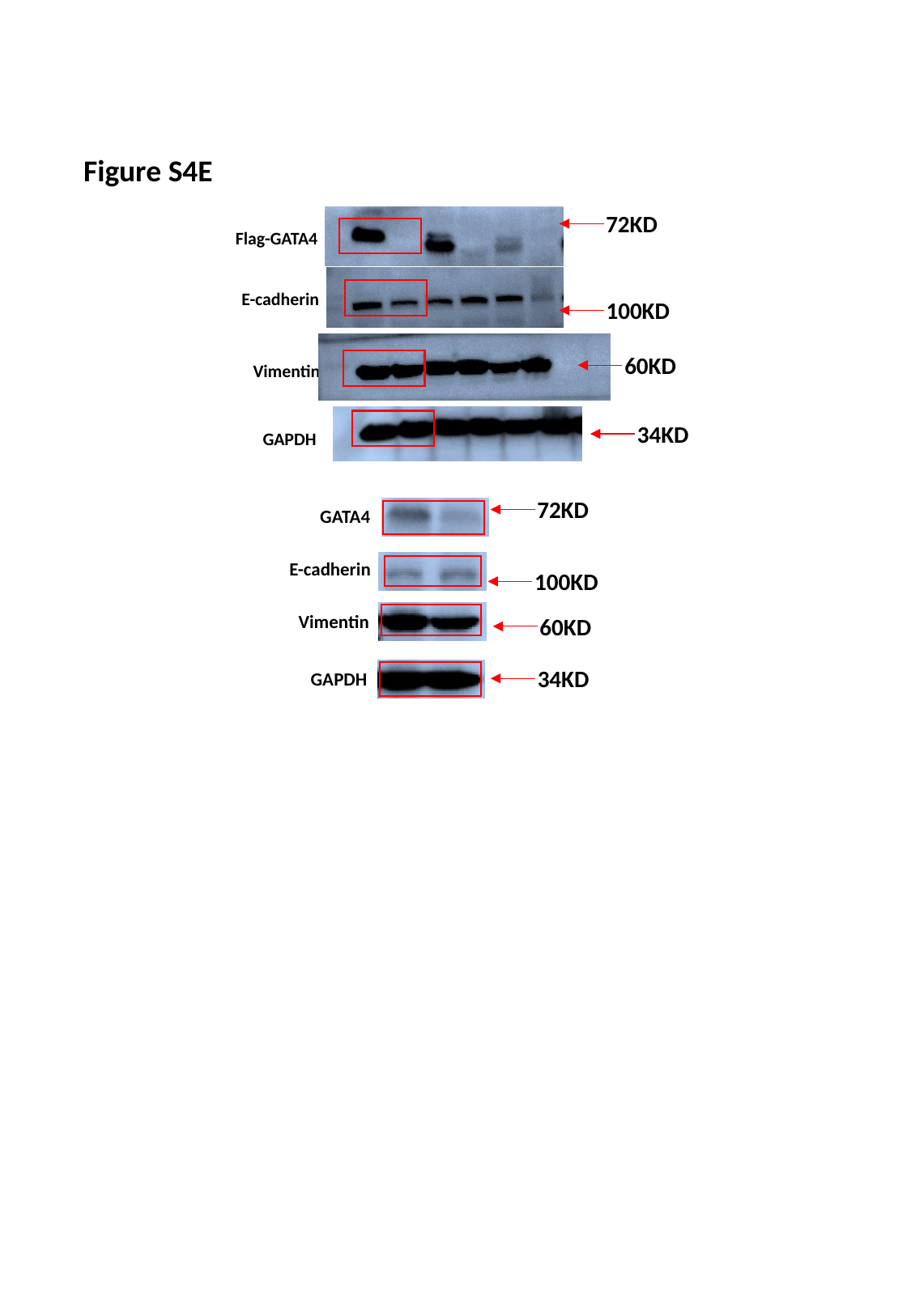

Figure S4E
72KD
Flag-GATA4
E-cadherin
100KD
60KD
Vimentin
34KD
GAPDH
72KD
GATA4
E-cadherin
100KD
Vimentin
60KD
34KD
GAPDH

## Slide 6
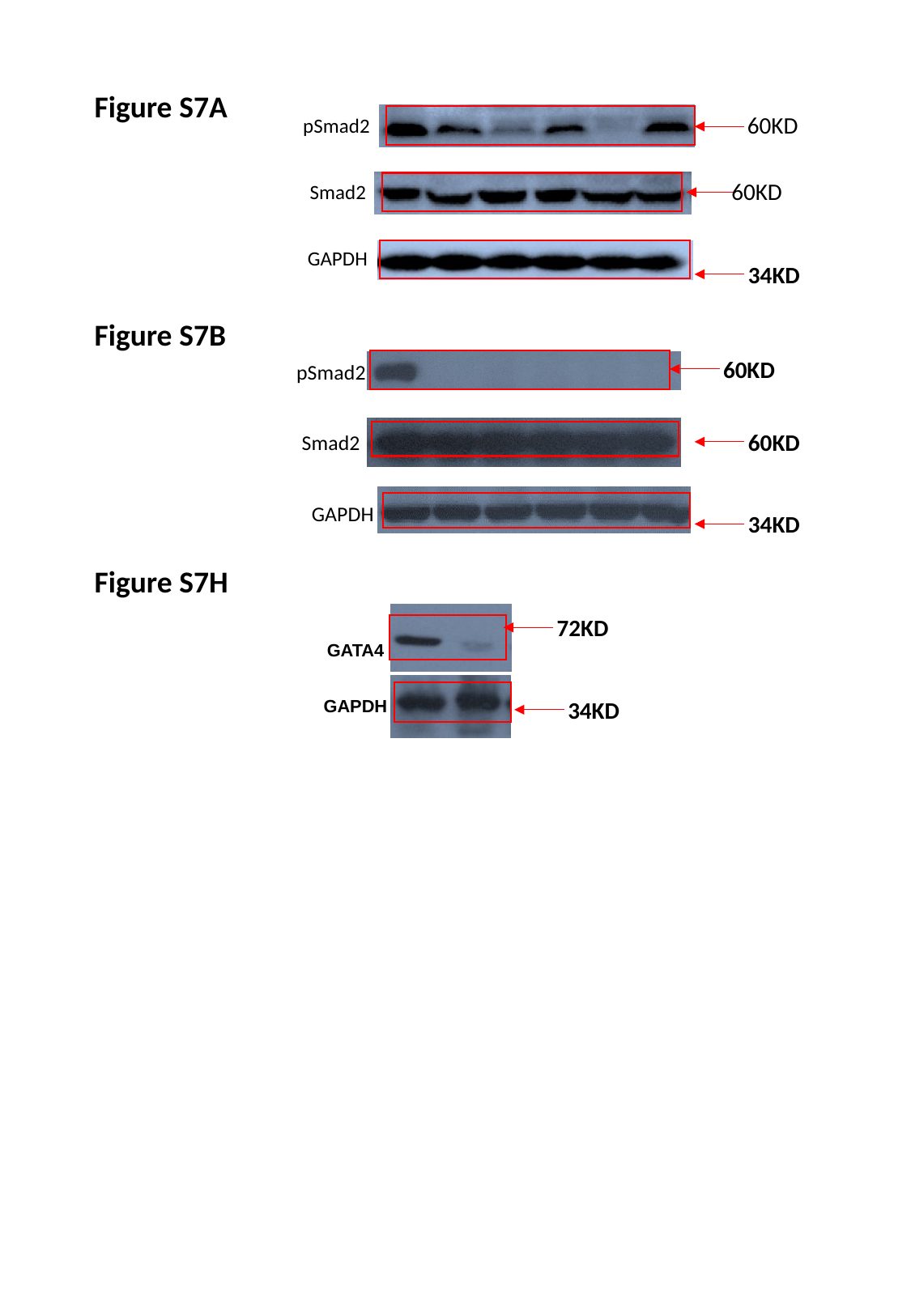

Figure S7A
60KD
pSmad2
60KD
Smad2
GAPDH
34KD
Figure S7B
60KD
pSmad2
60KD
 Smad2
GAPDH
34KD
Figure S7H
72KD
GATA4
GAPDH
34KD
