# Supplementary figures and images for: Author Correction: Lung cancer deficient in the tumor suppressor GATA4 is sensitive to TGFBR1 inhibition
Source: Nat Commun. 2025 Sep 18;16:8316. doi: 10.1038/s41467-025-63723-5 (PMC12446457; doi:10.1038/s41467-025-63723-5)

Figure 4c

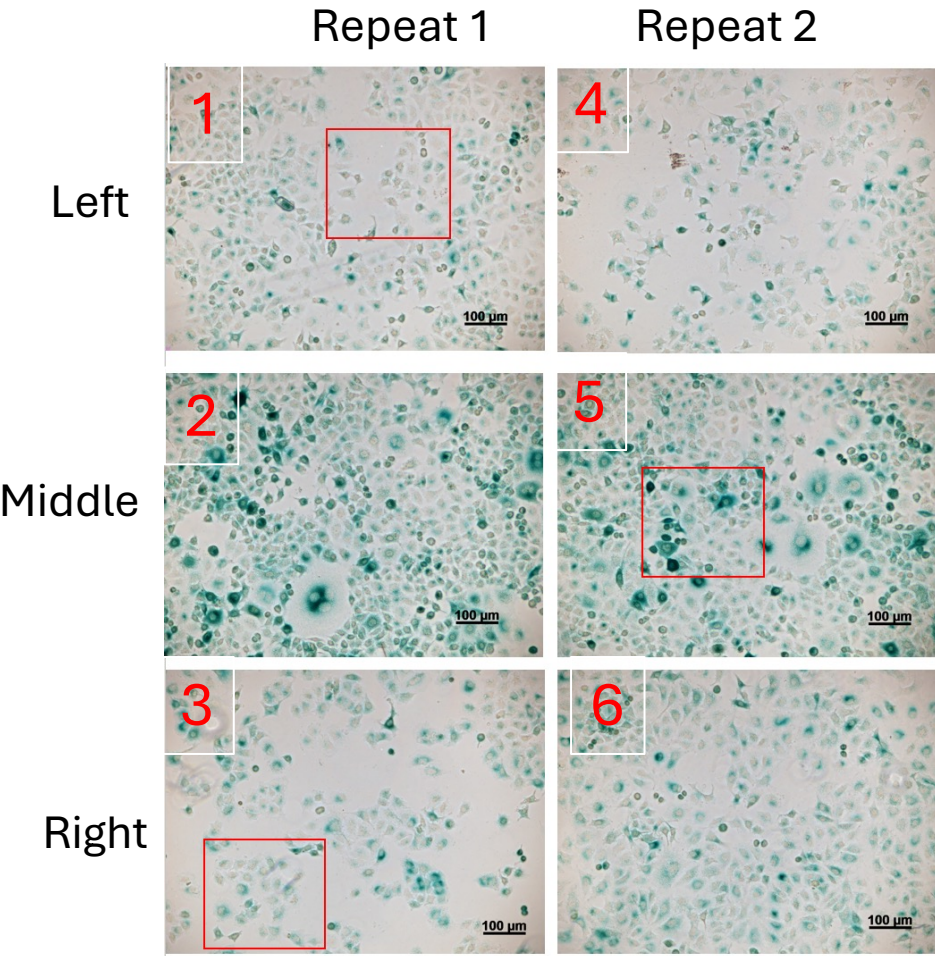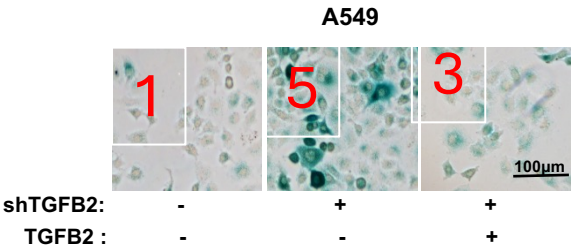

Figure 4i figures

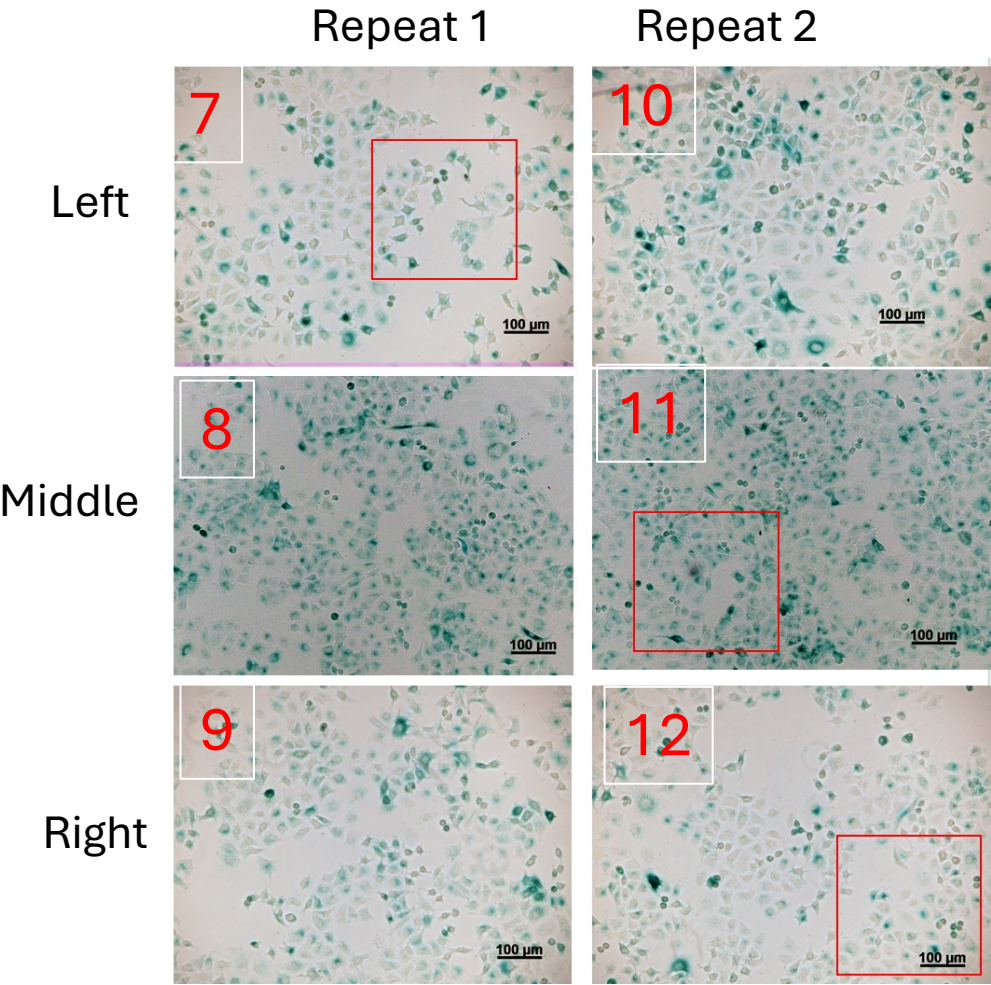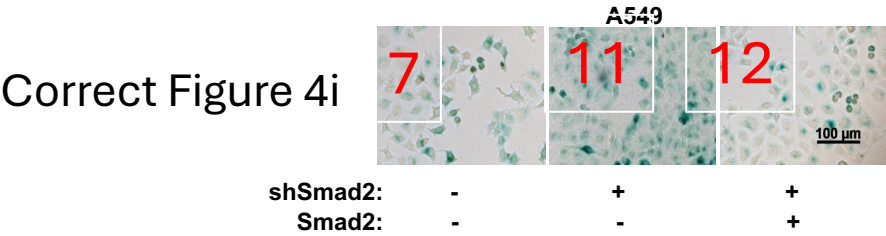

Published incorrect Figure 4i

i

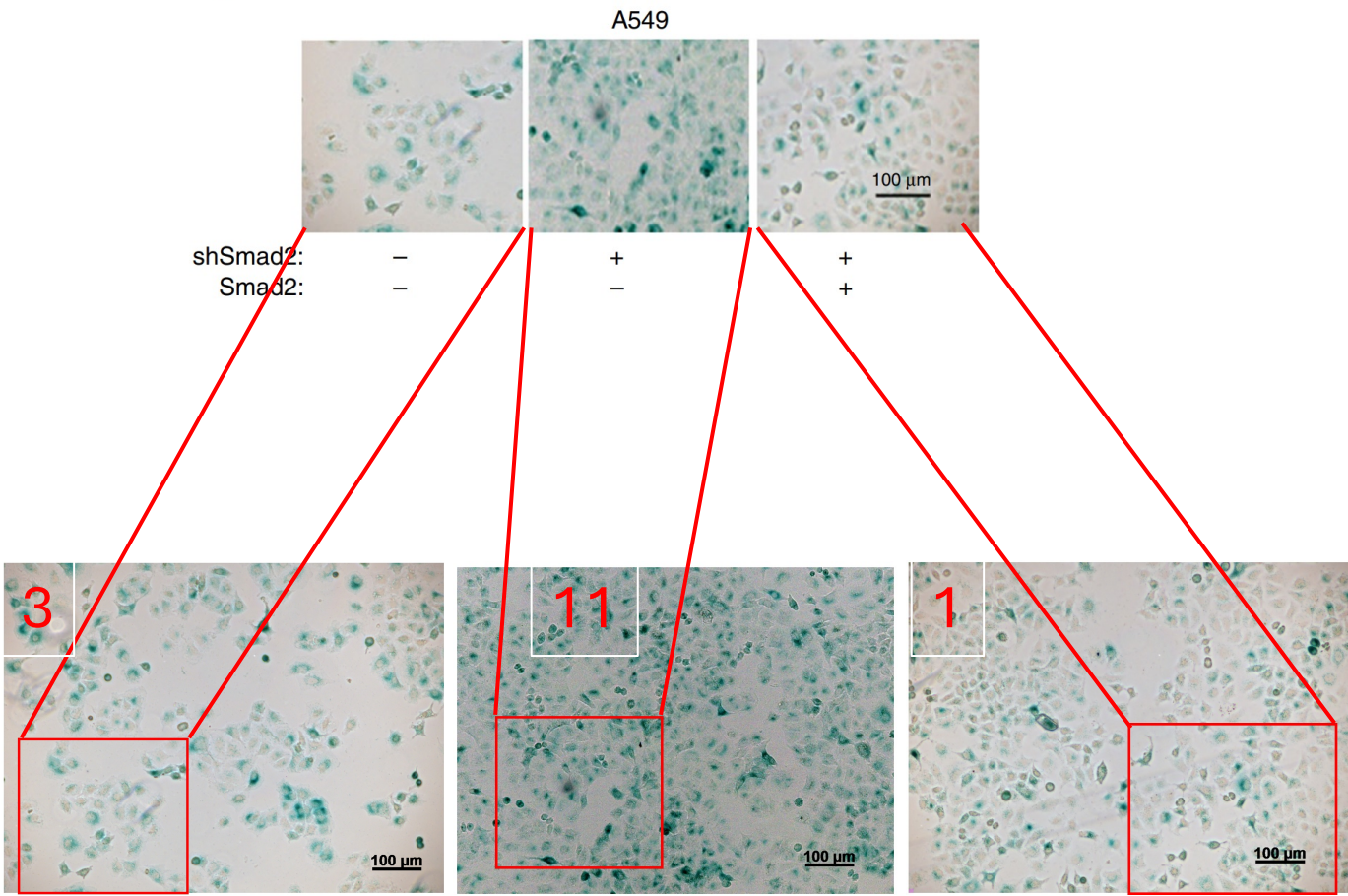

Uncropped figures

Supplement: Supplementary file 1 — Figure 4c,i raw data [file 41467_2025_63723_MOESM1_ESM.pdf]
